# miRNA profiling of B16F10 melanoma cell exosomes reveals melanin synthesis-related genes

**DOI:** 10.1016/j.heliyon.2024.e30474

**Published:** 2024-04-29

**Authors:** Gyeongchan Jeon, Ae Rim Hwang, Dae-Young Park, Ji-Hun Kim, Yang-Hoon Kim, Byung-Kwan Cho, Jiho Min

**Affiliations:** a Graduate School of Semiconductor and Chemical Engineering, Jeonbuk National University, 567 Baekje-daero, Deokjin-gu, Jeonju-si, Jeollabuk-do, 54896, Republic of Korea; b Department of Microbiology, Chungbuk National University, Chungdae-Ro, Seowon-Gu, Cheongju, 28644, Republic of Korea; c Department of Biological Sciences, Korea Advanced Institute of Science and Technology, 291 Daehak-ro, Yuseong-Gu, Daejeon, 34141, Republic of Korea

**Keywords:** Melanocyte exosomes, Melanogenesis regulation, MicroRNA profiling, Intercellular communication, Skin health

## Abstract

This study investigates the communication between skin cells, specifically melanocytes, keratinocytes, and fibroblasts, which is crucial for the process of melanin production known as melanogenesis. We aimed to understand the role of melanocyte exosomes in regulating melanogenesis and to uncover the microRNAs influencing this process. We isolated exosomes and characterized them using advanced microscopy and protein analysis to achieve this. We conducted experiments on melanoma cells to study melanin production regulation and examined how exosomes influenced gene expression related to melanogenesis. The results revealed that melanocyte exosomes increased certain types of tyrosinases, thereby enhancing melanin production. Furthermore, we acquired the miRNA profile of exosomes and hypothesized that specific siRNAs, such as miR-21a-5p, could potentially facilitate melanin synthesis. Our findings shed light on the importance of exosomes in skin health and provide valuable insights into intercellular communication mechanisms. Understanding these processes can pave the way for innovative therapies to treat melanin-related disorders and maintain healthy skin.

## Highlights

• Melanocyte exosomes regulate melanogenesis via miRNAs.
• Exosomes upregulate TYRP1 and downregulate MITF.
• B16F10 derived exosomes contain numerous melanogenesis-related genes.
• Exosomes of skin cells play an important role in intercellular communication

## 1 Introduction

Melanin is a natural pigment found in many organisms and is synthesized by tyrosine oxidation in melanocytes located in the dermis of the skin. The synthesized melanin is then transferred to the basal layer of the epidermis, where it is received by keratinocytes [1]. The intercellular communication between keratinocytes and melanocytes is stimulated by ultraviolet radiation, which induces the production of α-melanocyte-stimulating hormone (α-MSH) in keratinocytes [2]. Intercellular communication can occur using extracellular vesicles such as exosomes [3,4]. Studies are currently ongoing to investigate the role of exosomes in intercellular communication [5]. Exosomes are secreted by most eukaryotic cells, and they are derived from the endosomal compartment [6]. Recent studies have revealed the important roles played by exosomes in normal skin physiology, including the regulation of inflammatory cytokines [7], collagen synthesis [8], proliferation, differentiation of skin cells [9], and melanogenesis [10]. It has been reported that exosomes secreted by keratinocytes increase the expression and activity of proteins related to melanosome synthesis, thereby inducing melanin synthesis [10]. Exosomes have also been shown to be involved in the pathological processes associated with several skin diseases, including psoriasis, melanoma, and scleroderma [[11], [12], [13]]. The use of exosomes as biomarkers or therapeutic agents is an area of increasing interest [14].

To better understand the biological processes occurring in the skin, additional studies are necessary to investigate the communication between exosomes and various skin cell types such as melanocytes, keratinocytes, and fibroblasts. The content of exosomes varies depending on their cellular origin and may include various biologically active substances such as proteins, mRNAs, and miRNAs [15]. Given that exosomes act as intercellular mediators, the analysis of the mRNA and miRNA content within exosomes is essential. To this end, large-scale transcriptomic and proteomic analyses have been conducted [16]. Recently, a study found that exosomal miRNA derived from keratinocytes regulates melanogenesis in melanocytes [17].

In our study, we isolated exosomes from B16F10 melanoma cells and characterized the morphology and protein expression within the exosomes using various methods, including field-emission scanning electron microscopy (FE-SEM), bio-transmission electron microscopy (Bio-TEM), an exosome quantification kit, and western blotting. We investigated the effect of melanocyte-derived exosomes on melanogenesis in B16F10 cells and confirmed that the exosomes promote melanin synthesis. We assessed the expression of mRNA related to melanin production in B16F10 cells after co-incubation with exosomes and found that the exosomes promote melanin production by increasing tyrosinase-related protein 1 (TYRP1) expression and decreasing microphthalmia-associated transcription factor (MITF) expression in the cells. Our finding is of particular interest since MITF is known to upregulate the expression of proteins involved in melanin synthesis, such as tyrosinase and TYRP1 [18]. We also conducted miRNA profiling of the exosomes and found the expression of many melanogenesis-related genes in the exosomes isolated from B16F10 cells. Our study provides initial insights into the mechanisms of exosomal regulation of melanin production in melanocytes and miRNA profiling data of the exosomes. Further studies are necessary to understand the intercellular communication between various cell types in skin tissue and the potential use of exosomes as a novel therapeutic option to improve melanin production disorders or leukoplakia.

## 2 Materials and methods

### 2.1 Cell culture and exosome isolation

The mouse melanoma cell line B16F10 (KCLB No: 80008) was obtained from the Korean Cell Line Bank (KCLB). The standard culture medium for B16F10 cells was Dulbecco's modified Eagle medium (DMEM) supplemented with 10 % fetal bovine serum (FBS), 20 mM hydroxyethyl piperazine ethane sulfonic acid (HEPES), and 1 % penicillin-streptomycin, and the cells were maintained at 37 °C in 5 % CO_2_. Exosomes were isolated from the cell culture medium by seeding 5 × 10^6^ cells into a 150 mm dish and incubating for 24 h, followed by incubation in exosome-depleted growth medium for 48 h. The exosome-depleted medium was then centrifuged at 110,000×*g* for 70 min to remove serum exosomes. To isolate exosomes from the cell culture medium, the medium was collected and centrifuged at 1000×*g* for 5 min to remove whole cells, and the supernatant was transferred to high-speed centrifuge tubes on ice and centrifuged at 13,000×*g* for 30 min, and filtered with a 0.2 μm pore size filter. The filtered medium was then centrifuged at 110,000×*g* for 70 min in an ultracentrifuge with a 70 Ti rotor to isolate the exosomes, which were subsequently washed with PBS and resuspended in PBS.

### 2.2 Exosome concentration and protein isolation

The exosomes were concentrated using Amicon® Ultra 0.5 mL centrifugal filters with a 100 kDa molecular weight cut-off (Merck Millipore, USA). 0.5 mL exosome solution was added to the filter assembled in a micro tube and centrifuged at 10,000×*g* for 10 min. To recover the filtered exosome solution, the filter was assembled upside down and centrifuged at 10,000×*g* for 2 min. The protein within the exosomes was isolated using RIPA lysis buffer containing 150 mM NaCl, 50 mM Tris (pH 8.0), 1.0 % NP-40, 0.5 % sodium deoxycholate, and 0.1 % SDS. The exosome sample was mixed with RIPA lysis buffer, protease inhibitor cocktail (PIC) at a 10 : 10: 1 ratio, and phenylmethanesulfonyl fluoride (PMSF) at a 1 : 1 ratio. The mixture was incubated at room temperature for 5 min, followed by centrifugation at 10,000×*g* for 5 min, and the supernatant was transferred into new tubes. The lysed exosome proteins were further concentrated using Amicon® Ultra centrifugal filters with a 10 kDa molecular weight cut-off.

### 2.3 Field emission scanning electron microscopy (FE-SEM)

The exosome samples were prepared using a dry method. All samples were washed with filtered distilled water (DW) and resuspended to remove foreign substances such as salt. Then, 10 μL of exosomes suspended in solution was dropped onto clean aluminum foil or a cover glass and dried at room temperature overnight while protected from dust exposure. Prepared samples were examined under a field emission scanning electron microscope (SUPRA40VP, Carl Zeiss, Germany) installed at the Center of University-Wide Research Facilities (CURF) at Jeonbuk National University.

### 2.4 Bio-transmission electron microscopy (Bio-TEM)

A diluted exosome solution was dropped onto 400 mesh electron microscopy grids (Electron Microscopy Sciences, USA) and left for 20 min for adsorption and fixation. The grid was blotted with filter paper and dried, after which 10 μL of 2 % uranyl acetate was added to the grid. The uranyl acetate was then removed, and 10 μL of DW was added for 1 min. The water droplet was removed, and the grid was left to air dry for 15 min. The prepared samples were examined under a bio-transmission electron microscope (H-7650, Hitachi, Japan) installed at the Center of University-Wide Research Facilities (CURF) at Jeonbuk National University.

### 2.5 Exosome quantification

An EXOCET exosome quantitation kit (System Biosciences, USA) was used for exosome quantification. This step was performed according to the manufacturer's instructions. The isolated exosomes were mixed with lysis buffer and incubated at 37 °C for 5 min. After 5 min, the samples were vortexed for 15 s and then centrifuged at 1500×*g* for 5 min. The supernatant was transferred to a new test tube on ice. Next, 50 μL of lysed exosome protein sample was mixed with 50 μL of premix solution (prepared by mixing buffer A and B from the kit), per well. Then, the mixture-containing 96-well plate was incubated at room temperature for 10 min, and the absorbance was measured at 405 nm. The amount of exosome particles was calculated by comparing absorbance results to a standard curve. Bradford and BCA protein assays were performed to quantify the protein concentration of the exosomes. Briefly, we prepared a standard curve using dilutions of bovine serum albumin (BSA). The Bradford reagent was reacted with exosome protein samples for 5 min in darkness. Then, the absorbance was measured at 595 nm and calculated using the standard curve.

### 2.6 Western blotting

The exosome pellets in ultracentrifugation tubes were lysed by the direct addition of RIPA buffer. After lysis, the protein concentration was quantified, and then we prepared western blotting samples with 20 μg of exosome protein. To detect proteins, the lysates were subjected to SDS-PAGE on 10 % acrylamide gels, with electrophoretic transfer to nitrocellulose membranes. The membranes were incubated with primary antibodies (1 : 1000) and secondary antibodies (1 : 5000), and proteins were visualized using an ECL kit (Elpis Biotech, South Korea). The following antibodies were used: mouse anti-TSG101 antibody (Abcam, UK), rabbit anti-CD81 (Abcam, UK), mouse anti-histone H2B (Abcam, UK), and horseradish peroxidase (HRP)-conjugated goat anti-mouse IgG (heavy and light chain [H/L]; Invitrogen, USA) and goat anti-rabbit IgG (H/L; Abcam, UK).

### 2.7 Cell viability test

The cytotoxicity of the exosomes and reagents was assessed using MTT assay, a general method used to determine the effect of a sample on the viability of cells. First, 10^4^ cells were seeded into each well of a 96-well plate and cultured for 24 h with the appropriate medium for each cell type. The cells were starved for 24 h before treatment by culturing them in serum-free media. Then, the exosome solution and reagents were added at the appropriate concentration and maintained for 24 h at 37 °C in a humidified atmosphere with 5 % CO_2_. All wells were then washed, and 200 μL of 0.5 mg/mL thiazolyl blue tetrazolium bromide (MTT, Sigma, USA) solution dissolved in the culture medium was added. After incubating for 2 h, the MTT solution was removed, and 200 μL of dimethyl sulfoxide (DMSO, Sigma, USA) was added to each well. Finally, the absorbance was measured at 570 nm with an ELISA microplate spectrophotometer (Multiskan SkyHigh, Thermo Scientific, USA).

### 2.8 Melanin synthesis regulation assay

The melanin assay was based on the 2015 National Institute of Food and Drug Safety Evaluation Guidelines for evaluating the effectiveness of functional cosmetics. For the assay, 2 × 10^6^ B16F10 cells per well were seeded into a 100-mm cell culture dish and incubated for 24 h to adhere. The medium in each well was washed and exchanged with medium containing various concentrations of exosomes and 10 nM alpha-melanocyte-stimulating hormone (α-MSH, Sigma, USA), and the plates were then incubated for 72 h. After that, to measure the extracellular melanin concentration, 1 mL of cell culture medium was collected into microtubes and centrifuged at 10,000×*g* for 10 min to remove cells and cell debris. Then, 200 μL of supernatant was loaded into 96-well plates, and the absorbance was measured at 490 nm. To measure the intracellular melanin concentration, cells were detached with Tris-EDTA buffer solution (Gibco, USA) and centrifuged at 1000×*g* for 5 min to harvest the cells. Pelleted cells were heated with 1 M NaOH containing DMSO at 80 °C for 2 h to lyse melanin compounds. Finally, the absorbance of the isolated melanin was measured at 490 nm. To quantify the extracellular and intracellular melanin concentration, a standard curve was prepared using a synthetic melanin standard (Sigma, USA).

### 2.9 Reverse transcription–polymerase chain reaction (RT-PCR)

Total RNA was isolated from cells using an RNA isolation kit (Ribospin™ vRD II, GeneAll, Korea). Reagents were prepared as needed according to the manufacturer's instructions. After preparation, denaturing solution was added to the sample and mixed thoroughly. The mixture was placed on ice for 5 min, and acid-phenol and chloroform were added to the sample and mixed for 1 min. Finally, the samples were centrifuged at 10,000×*g* for 5 min, and the aqueous phase was carefully transferred into a fresh tube without disturbing the lower phase or interphase. The isolated RNA was quantified using a Qubit™ fluorometer (Invitrogen™, USA). 25 μg of RNA was used to perform reverse-transcription step. After reverse-transcription, the cDNA sample was amplified through a PCR step using pre-designed mRNA primers. The primers used for RT-PCR are listed in Table 1. Amplified cDNA was loaded onto Tris-borate-EDTA (TBE) 1 % agarose gel with ethidium bromide (ETBR, Thermo Fisher Scientific, USA) and electrophoresed at 100 V for 15 min. The bands on the gels were scanned under UV light, and the intensity was quantified using Image J software. The intensity of the target genes was compared with that of a housekeeping gene (GAPDH) for visualization and data analysis. The data were log-transformed using log_2_ to express the fold change.

**Table 1:** List of primers used for the reverse transcription polymerase chain reaction.

Genes	Forward primer sequence	Reverse primer sequence	PCR product size (base pairs)
*Mus musculus* glyceraldehyde-3-phsphate dehydrogenase (GAPDH)	5′-gtgtttcctcgtcccgtaga-3′	5′-ccttccacaatgccaaagtt-3′	536
*Mus musculus* CD81 antigen (CD81)	5′-cgtcatgatccacagaccac-3′	5′-gcaccatgctcagaatcatc-3′	571
*Mus musculus* CD9 antigen (CD9)	5′-gactatggctccgattcgac-3′	5′-gcacaggatcatgctgaaga-3′	554
*Mus musculus* topoisomerase (DNA) 1 (TOP1)	5′-gaaggagagacggcagacac-3′	5′-cccttcgagcatctgctaac-3′	508
*Mus musculus* melanogenesis-associated transcription factor (MITF)	5′-tcgacctctacagcaaccag-3′	5′-atggtaccgtccgtgagatc-3′	580
*Mus musculus* mRNA for tyrosinase	5′-ccattttcctcgagcctgtg-3′	5′-gaaaccctggtgcttcatgg-3′	569
*Mus musculus* tyrosinase-related protein 1 (TYRP1)	5′-tcatcaggaagaggcaggtg-3′	5′-atgtctctctccagctgcag-3′	530

### 2.10 MicroRNA analyses

The analysis of microRNAs present in the exosomes isolated from B16F10 melanoma cells was performed through e-biogen's Affymetrix microarray service (e-biogen Inc, South Korea). The transcriptome profiling was conducted using a GeneChip® Scanner (ThermoFisher Scientific, USA) and mouse expression profiling arrays & assays reagent. Expression microarrays simultaneously measure the expression of thousands of RNA transcripts. The number of reads of each miRNA was received as raw data, and we normalized the data to log_2_. The normalized data were classified according to gene function and the expression levels were compared. In this process, we used ExDEGA software, and the gene category was selected using the miRWalk 2.0 website.

### 2.11 Data analyses

All data were obtained from three simultaneous independent experiments. The standard deviation was also calculated and considered. The image data were analyzed by Image J, ZEN software and all data were visualized using SigmaPlot software.

## 3 Results

### 3.1 Exosome isolation from B16F10 and characterization

#### 3.1.1 Exosomes isolation from B16F10 and quantification

To isolate cell-derived exosomes with high purity, we used exosome-depleted serum that removed small inner vesicles. As previously reported in many studies, we found that FBS contained many vesicles [19]. Therefore, exosome depletion was performed for our experiments. The exosome isolation method was based on various previously published methods [[12], [13], [14]]. After concentration and filtration steps, we successfully isolated exosomes from the growth medium and characterized them using an exosome quantitation kit, FE-SEM, Bio-TEM, and western blotting. The EXOCET exosome quantification kit can quickly quantify exosome numbers by measuring the activity of acetylcholinesterase (AChE), an enzyme that has been shown to be highly enriched in exosomes [20,21]. The presence of AChE in exosomes from serum, stem cells, and cancer cell lines has been verified using mass spectrometry and western blotting. In our system, we were able to recover 8 × 10^8^ exosome particles from 20 mL of growth medium when cultured in a 150-mm cell culture dish.

#### 3.1.2 FE-SEM and Bio-TEM analysis of B16F10-derived exosomes

**Fig. 1:**
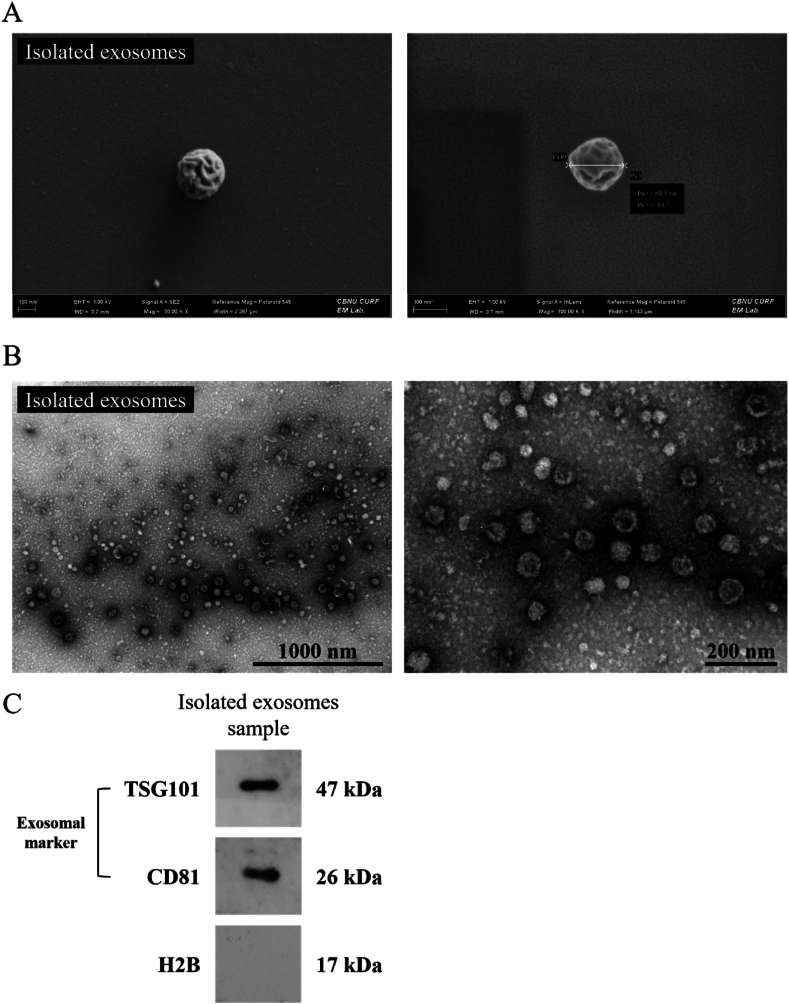
Characterization of exosomes derived from B16F10 cells. To confirm the morphology of exosomes, 20 μg of exosomes were used. (A) Field emission scanning electron microscope images (100 nm scale bar). (B) Bio-transmission electron microscope images (1000 nm and 200 nm scale bar). (C) Western blotting of 20 μg of exosomes using antibodies against exosome (ant-TSG101 and -CD81) and nucleosome (anti-histone-H2B) markers.

FE-SEM and Bio-TEM were used for morphological analysis of the isolated exosomes. Exosomes were enriched and pretreated for SEM and TEM analysis, as described in the methods section. Under FE-SEM, the exosomes appeared rounded and slightly distorted, which is a natural phenomenon that may occur during the drying process. The observed exosome particles had sizes of approximately 53.59 nm, 103.8 nm, and 185.3 nm, and some vesicles over 200 nm were also observed (Fig. 1A). When viewed under TEM, cup-shaped exosomes were identified with clearer negative staining, similar to the images of exosomes presented in published papers. The size of the exosomes was determined to be approximately 30–150 nm (Fig. 1B).

#### 3.1.3 Western blotting analysis of B16F10-derived exosomes

Western blotting experiments were conducted to qualitatively characterize the isolated exosomes at the protein level. Among the cluster of differentiation series (CD series), CD81, a tetraspanin protein present in the exosome membrane, and TSG101, a protein involved in the formation of multi-vesicular bodies (MVB), were used as exosome markers [4,22]. In addition, histone H2B, a nucleosomal protein found in essentially all eukaryotic cells, was used as a negative control [23]. The western blotting results are presented in Fig. 1C. The exosome marker bands TSG101 and CD81 were clearly identified on the western blotting membranes, with the TSG101 band at 47 kDa and the CD81 band at 26 kDa. The histone protein marker H2B, which should have appeared at 17 kDa, was not identified on membranes containing exosome proteins.

### 3.2 Functional evaluation of B16F10-derived exosomes

**Fig. 2:**
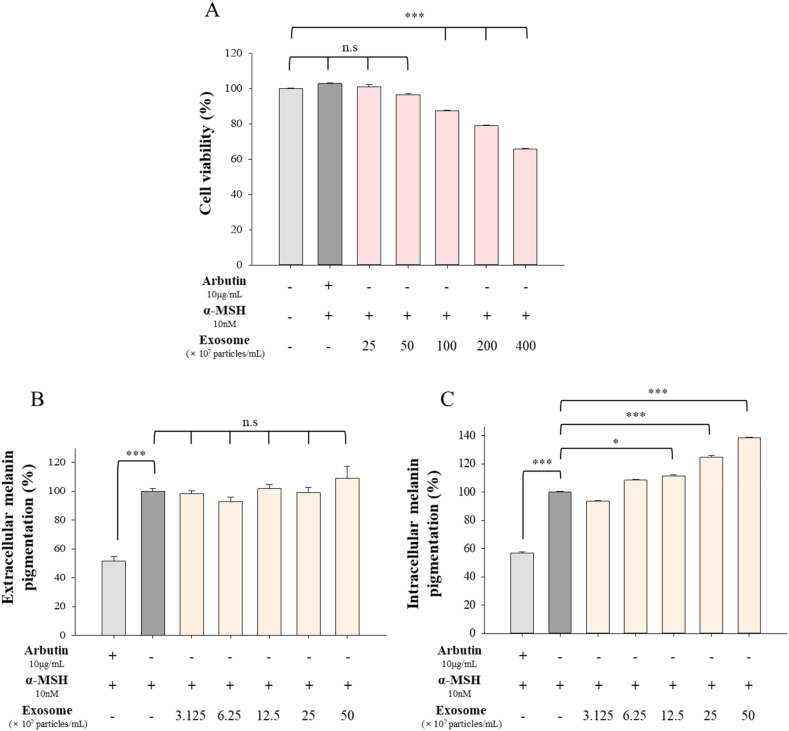
Melanin synthesis regulation test. (A) The cytotoxicity of the exosomes derived from B16F10 cells was confirmed using MTT assay in B16F10 cells. (B) After treatment with exosomes for 72 h, extracellular melanin content was measured in the cell culture medium. (C) Intracellular melanin content was measured in the cell lysates. The melanin concentration was quantified by using a standard curve prepared from a synthetic melanin standard. Data are presented as the means ± SD (n = 3). *P < 0.05, **P < 0.01 and ***P < 0.001.

The exosomes isolated from B16F10 melanoma cells were quantified and added to B16F10 cell cultures at concentrations of 3.125, 6.25, 12.5, 25, or 50 × 10^7^ particles/mL to evaluate their effects on melanin synthesis. Before proceeding with the melanogenesis regulation test, a cell viability assay was completed to assess the cytotoxicity of the exosomes and assay reagents. All subsequent experiments were conducted within a concentration range that did not produce cytotoxicity (Fig. 2A). Melanin content was assessed in two ways: extracellular melanin (the melanin pigment released from the cells) and intracellular melanin produced within the cell (Fig. 2B and C). Arbutin, known as a melanogenesis inhibitor, was treated as a positive control. Arbutin was added to the cultures at 10 μg/mL and was not cytotoxic. Fig. 2C shows that the amount of melanin in cells increased depending on the particle concentration of the added exosomes, while extracellular melanin production was similar regardless of the treatment conditions. Specifically, at concentrations of 12.5 × 10^7^ particles/mL or higher, exosomes notably enhanced melanin synthesis, particularly increasing intracellular melanin content by over 30 % at a concentration of 50 × 10^7^ particles/mL. These results suggest that exosomes isolated from melanocytes promote melanogenesis.

### 3.3 Distinct expression of melanin-synthesis-related genes in B16F10-derived exosomes

**Fig. 3:**
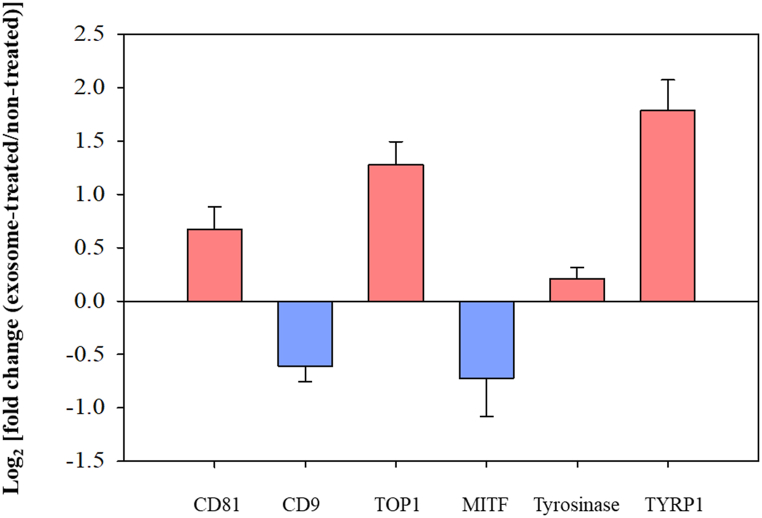
Changes in mRNA expression (fold change) in exosome-treated cells and untreated control cells. The mRNA expression was quantified using a reverse transcription polymerase chain reaction (RT-PCR). Each gene was transcribed using the indicated primers and quantified using electrophoresis images. For visualization, we normalized the data to log_2_. Data are presented as the means ± SD (n = 3).

To investigate the mechanism of melanin synthesis promotion by exosomes isolated from melanocytes, we performed RT-PCR to determine the expression of melanin-synthesis-related mRNA in exosome-treated and untreated control cells (Fig. 3). RNA was extracted from the B16F10 cells, and reverse transcription was performed to obtain cDNA. We focused on mRNAs related to the melanogenesis process, including TYRP1, MITF, and tyrosinase. We also examined the expression of a factor related to cell proliferation, DNA topoisomerase 1 (TOP1), and exosome-regulation factors, CD81 and CD9. A log2-fold-change value greater than 1 or less than −1 indicates up- or down-regulation of mRNA expression, respectively. TYRP1 expression increased by more than 4-fold in exosome-treated B16F10 cells, while MITF was down-regulated, indicating that the melanin-synthesis-inducing function of exosomes was correlated with tyrosinase activity. Additionally, TOP1 expression was induced by exosomes. Our results suggest that a relatively larger amount of mRNA related to cell proliferation is contained in the exosomes secreted from B16F10.

### 3.4 MicroRNA profiling of in B16F10 derived exosomes

**Fig. 4:**
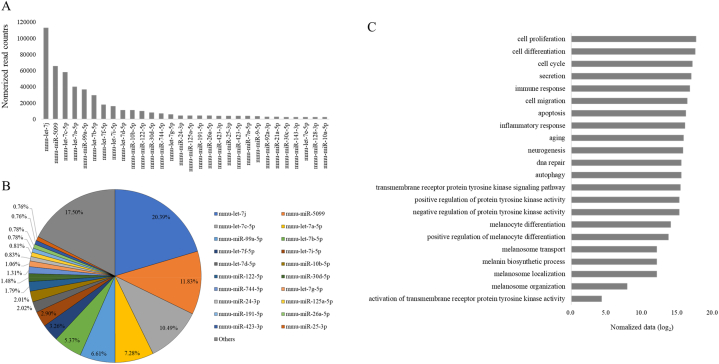
The microRNA profile of B16F10-derived exosomes. The Affymetrix microarray platform was used for miRNA sequencing, and the data was analyzed with ExDEGA software. (A) Total reads of the top 30 miRNAs are shown. (B) The top 20 miRNAs accounted for 82.5 % of total miRNAs present in B16F10-derived exosomes. (C) Processes targeted by the miRNAs in B16F10 exosomes were ranked based on significance from high to low. All miRNAs contained within B16F10 exosomes were analyzed using miRwalk miRNA database to determine the target gene landscape. For visualization, we normalized the data to log_2_.

Exosomes contain various miRNAs involved in functions such as tissue regeneration [[24], [25], [26]]. There is growing interest in the use of exosomal miRNA as biomarkers of disease for diagnosis and to monitor disease recurrence [27]. Here, we used miRNA profiling to determine the role of miRNA in melanocyte exosomes in the melanin synthesis process. The miRNAs of melanocyte exosomes were profiled using GeneChip® miRNA arrays and rank ordered according to read counts after normalization to total read counts (Fig. 4A). Fig. 4B shows that the top 20 miRNAs accounted for 82.5 % of all the miRNA content and the remaining 757 miRNAs made up a very low percentage of the total reads (0.01 %–0.71 %). B16F10-derived exosome sample information is described inSupplementary Table 1. All identified miRNA data are listed in Supplementary Table 2. The miRwalk miRNA database was used to identify the biological processes and pathways impacted by the exosomal miRNAs (Fig. 4C) [28]. We classified the data by gene category and log-transformed the read counts to visualize the data. Most genes contributed substantially to specific processes or functions essential for cell survival such as cell proliferation, differentiation, cell cycling, migration, inflammatory response, and DNA repair. However, there are considerable amounts of melanogenesis-related genes such as melanosome transport, localization, organization, melanin biosynthesis, and tyrosine kinase activity-related processes in the exosomes. All miRNA sequences are provided in the Supplementary Table 2.

## 4 Discussion

Here, we have verified that the size of the exosomes isolated from B16F10 melanoma cells is similar to the reported size of exosomes from other animal cells/cell lines, such as stem cells and cancer cell lines [29,30]. Additionally, we have observed clear expression of the exosome markers TSG101 and CD81 in the B16F10-derived exosomes. The TSG101 protein participates in the endosomal sorting complex required for transport (ESCRT) pathway, which facilitates budding and formation of multivesicular bodies (MVB), the parent of exosomes [4,22,29]. The CD81 protein, a member of the tetraspanin family, is highly enriched in extracellular vesicles, as indicated by recent proteomic data from the ExoCarta and EVPedia databases [[31], [32], [33]]. Moreover, we have confirmed the absence of H2B, a cytoskeletal protein predominantly found in the nucleus, in the exosomes [23]. This finding suggests that the purified exosomes were obtained without contamination from other biological organelles, such as nucleosomes.

In this study, we found that melanocyte exosomes were non-cytotoxic and promoted melanin accumulation in melanocytes. In RT-PCR analyses of melanocyte co-cultures with exosomes, we noted a significant increase in the expression of TOP1 and TYRP1, alongside a slight decrease in MITF expression in the cells. Tyrosinase plays a critical role in the synthesis of eumelanin, the most photoprotective black-brown melanin [34]. MITF protein plays a pivotal role in melanocyte lineage development and serves as a key transcription factor regulating the expression of tyrosinase and TYRP1 genes [18]. However, direct involvement of MITF in regulating melanin synthesis or the expression of endogenous tyrosinase or TYRP1 remains unproven. According to recent studies, MITF is required for melanin synthesis, but MITF is unable to induce the expression of tyrosinase and TYRP1 in B16 mouse melanoma cells or human melanocytes [35]. Moreover, it has been reported that the expression of tyrosinase is increased by cAMP induced through stimulation of MITF expression [36,37]. Additionally, exosomes did not upregulate all tyrosinases but specifically upregulated TYRP1. Consequently, we acknowledge that unknown regulatory pathways likely exist, which may regulate melanogenic gene expression and melanogenesis independently of MITF. Our findings suggest that exosomes regulate melanogenesis through a different pathway than melanin-synthesis-modulating drugs such as arbutin, which regulate MITF, tyrosinase, and TYRP1 [38]. Furthermore, exosomes increased TOP1 mRNA expression in RT-PCR results. TOP1 is a protein involved in DNA replication, crucial for preventing DNA damage, and has recently emerged as a potential target for anticancer chemotherapy in cancer research [39]. While this suggests a potential activation of cancer cells by tumor-derived exosomes, the observed effect on cell proliferation appears to be minimal, necessitating further research.

To determine the microRNA content of B16F10 cells, we conducted miRNA sequencing using miRNA array. We identified the presence of 777 miRNAs and categorized them based on gene function (Fig. 3). The functions of mmu-let-7j and mmu-mir-5099 miRNAs, which were the most highly expressed, have not been specifically identified at present. However, mmu-let-7c-5p, the next most expressed miRNA, has been reported to participate in over 50 processes, including DNA biosynthesis, cell proliferation, cell division, and apoptosis [[40], [41], [42]]. Furthermore, we analyzed the gene regulatory roles of exosome-composing miRNAs by consulting TagetScan and miRDB, online databases for miRNA target prediction and functional annotation. Consequently, we confirmed that several miRNAs, including miR-21a-5p and miR-145, can promote melanin production by targeting SOX (SRY-related HMG-box) genes or downregulating β-catenin and CDK2 protein expression [43,44]. However, additional experiments confirming the impact of individual miRNAs are necessary to establish a more accurate hypothesis.

Additionally, we found that the isolated exosome preparations in this study also contained various extracellular vesicles such as microvesicles and apoptotic bodies. Extracellular vesicles can be subdivided into exosomes, microvesicles, and apoptotic bodies according to their origin [45]: exosomes are derived from intracellular endosomal compartments, while microvesicles are particles released from the surface of cells, and apoptotic bodies are released by cells undergoing apoptosis [46]. Since each of these types of vesicles has a wide size distribution, it is difficult to separate them using only centrifugation [47].

Exosomes are the subject of investigations in various fields such as intercellular communication, biomarkers of specific diseases, and drug delivery systems [9,48,49]. However, their biological function is not yet clearly understood. In this study, we confirmed that B16F10-derived exosomes contained many melanin-synthesis-related miRNAs and contributed to melanin synthesis in melanocytes through a different pathway from other melanin synthesis regulators. These findings define the microRNA landscape in the melanocyte exosomes and identify a novel potential therapeutic strategy to improve melanin production disorders or leukoplakia. However, as the exosomes utilized in this study were derived from melanoma cells, they possess characteristics inherent to cancer cells. Consequently, numerous challenges exist in elucidating their specific effects on normal skin. To consider their application as a treatment for vitiligo, it is imperative to verify their impact on primary melanocytes obtained from healthy individuals and conduct additional clinical trials.

## 5 Conclusion

In this study, we successfully isolated and characterized exosomes derived from B16F10 melanoma cells. Additionally, it showed that the exosomes promoted melanogenesis in B16F10 melanoma cells, as evidenced by increased intracellular melanin content. Gene expression analysis demonstrated upregulation of TYRP1 and downregulation of MITF, suggesting the involvement of exosomes in modulating melanin synthesis-related genes. Furthermore, miRNA profiling revealed the presence of various miRNAs in the melanocyte-derived exosomes, with a significant impact on processes related to melanogenesis.

Overall, our findings contribute to a better understanding of the role of exosomes in melanogenesis and highlight their potential as mediators of intercellular communication in melanocytes. Further investigations are warranted to elucidate the precise mechanisms by which exosomes regulate melanogenesis and to explore their potential applications in therapeutic approaches targeting pigmentation disorders or skin-related conditions.

## Data availability statement

Data will be made available on request.

## Ethical issue

This article does not contain any studies with human participants or animals performed by any of the authors.

## CRediT authorship contribution statement

**Gyeongchan Jeon:** Writing – original draft, Visualization, Methodology, Investigation. **Ae Rim Hwang:** Visualization, Methodology, Investigation. **Dae-Young Park:** Methodology, Investigation. **Ji-Hun Kim:** Methodology, Investigation, Data curation. **Yang-Hoon Kim:** Writing – review & editing, Supervision, Project administration. **Byung-Kwan Cho:** Supervision, Project administration, Data curation, Conceptualization. **Jiho Min:** Writing – review & editing, Supervision, Project administration, Conceptualization.

## Declaration of competing interest

The authors declare that they have no known competing financial interests or personal relationships that could have appeared to influence the work reported in this paper.
