# Supplementary material for: miRNA profiling of B16F10 melanoma cell exosomes reveals melanin synthesis-related genes
Source: Heliyon. 2024 Apr 29;10(9):e30474. doi: 10.1016/j.heliyon.2024.e30474 (PMC11070906; doi:10.1016/j.heliyon.2024.e30474)
Supplement: Multimedia component 1 [file mmc1.pdf]

Supplementary Figure 1. Agarose gel electrophoresis of reverse transcription-PCR products for Tyrosinase, TYRP1 and GAPDH genes.

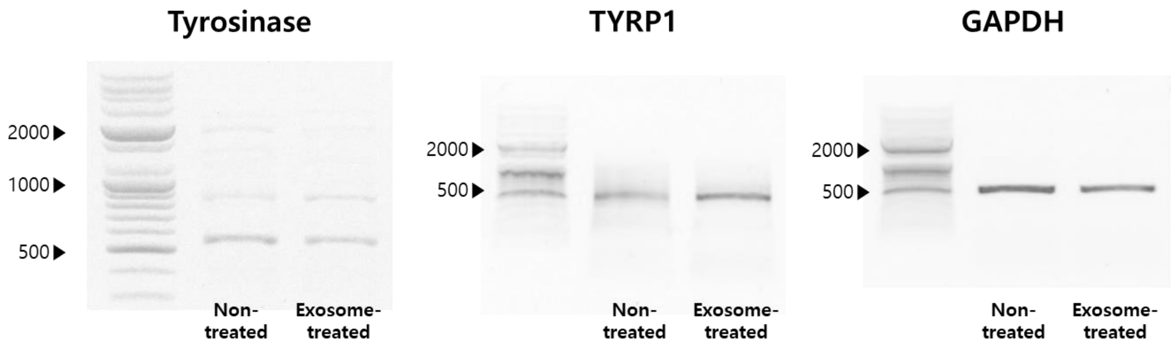

Supplementary Figure 2. Western blots original image files. (A) Figure 1C, western blotting images in main text. Western blots original images of (B) TSG101, (C) CD81 and (D) H2B. The selected sections indicated by the red square dotted line were used.

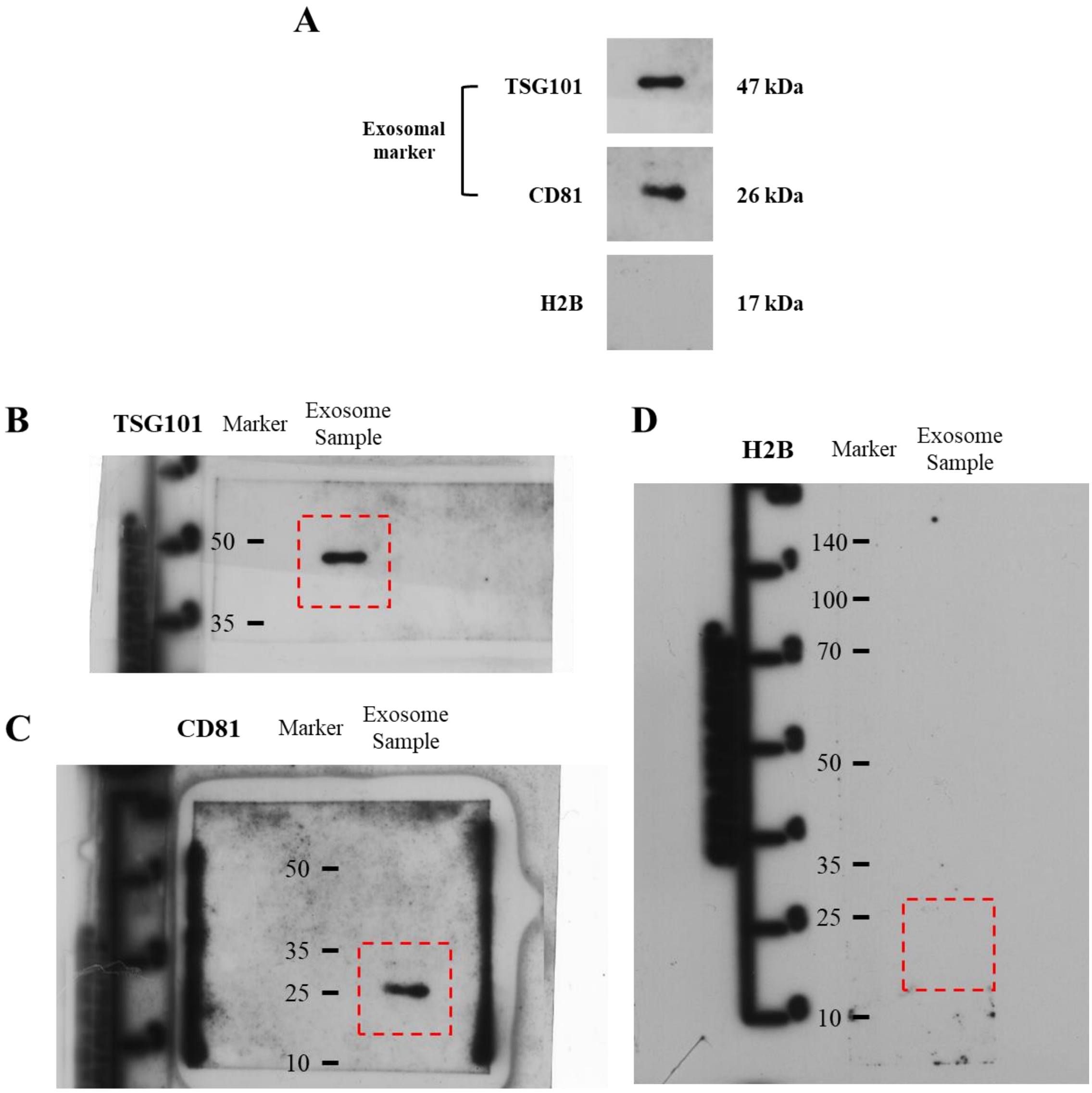

Supplementary Table 1. B16F10-derived exosome sample information for transcriptome analysis

Species: Mouse
Reference genome: mmu
Genome database: miRbase

No.	Sample	Nano-drop (ND 2000)				Bionanlyzer (pico)	
		ng/μl	Total (μg)	OD* 260/280	OD** 260/230	Ratio (28s/18s)	RIN***
1	B16F10 exosomes	70.7	0.848	1.90	0.90	0.0	2.5

* OD (260/230) is a metric indicating the degree of contamination from compounds like organic solvents and salts. A higher value suggests RNA of higher purity with minimal contamination.

** OD (260/280) is a metric reflecting the level of protein contamination. A higher value indicates RNA of higher purity with minimal protein contamination.

*** RIN (RNA Integrity Number) is a measure indicating the extent of RNA degradation. A higher RIN value suggests higher-quality RNA with minimal degradation.

Supplementary Table 2. B16F10 exosome miRNA Sequencing data. The Gene sequences were read by GeneChip® microarray and the annotation of each gene was confirmed using the ExDEGA analysis tool provided by e-biogen Inc. (Seoul, South Korea).

Total: 777	Gene symbol	Normalized data (log_2)	Raw data	Annotation									Target predicition
**ID**				**matual_id**	**chr**	**start**	**end**	**strand**	**type**	**Alias**	**Derives_from**	**sequence**	**miRBase search**
1	mmu-let-7g-5p	12.514	5848	mmu-let-7g-5p	chr9	106178846	106178867	+	miRNA	MIMAT0000121	MI0000137	UGAGGUAGUAGUUUGUACAGU	mmu-let-7g-5p
2	mmu-let-7i-5p	13.973	16079	mmu-let-7i-5p	chr10	122985698	122985719	-	miRNA	MIMAT0000122	MI0000138	UGAGGUAGUAGUUUGUGCUGU	mmu-let-7i-5p
3	mmu-miR-15b-5p	6.443	87	mmu-miR-15b-5p	chr3	69009775	69009796	+	miRNA	MIMAT0000124	MI0000140	UAGCAGCACAUCAUGGUUUAC	mmu-miR-15b-5p
4	mmu-miR-23b-5p	3.322	10	mmu-miR-23b-5p	chr13	63300492	63300512	+	miRNA	MIMAT0016980	MI0000141	GGGUUCCUGGCAUGCUGAUUU	mmu-miR-23b-5p
5	mmu-miR-27b-5p	1.000	2	mmu-miR-27b-5p	chr13	63300718	63300739	+	miRNA	MIMAT0004522	MI0000142	AGAGCUUAGCUGAUUGGUGAA	mmu-miR-27b-5p
6	mmu-miR-29b-1-5p	0.000	1	mmu-miR-29b-1-5p	chr6	31063067	31063088	-	miRNA	MIMAT0004523	MI0000143	GCUGGUUUCAUAUGGUGGUUU	mmu-miR-29b-1-5p
7	mmu-miR-30a-5p	10.713	1679	mmu-miR-30a-5p	chr1	23272274	23272295	+	miRNA	MIMAT0000128	MI0000144	UGUAAACAUCCUCGACUGGAA	mmu-miR-30a-5p
8	mmu-miR-30b-5p	7.140	141	mmu-miR-30b-5p	chr15	68337469	68337490	-	miRNA	MIMAT0000130	MI0000145	UGUAAACAUCCUACACUCAGC	mmu-miR-30b-5p
9	mmu-miR-99a-5p	15.160	36618	mmu-miR-99a-5p	chr16	77598940	77598961	+	miRNA	MIMAT0000131	MI0000146	AACCCGUAGAUCCGAUCUUGU	mmu-miR-99a-5p
10	mmu-miR-99b-5p	10.794	1776	mmu-miR-99b-5p	chr17	17830194	17830215	+	miRNA	MIMAT0000132	MI0000147	CACCCGUAGAACCGACCUUGC	mmu-miR-99b-5p
11	mmu-miR-124-5p	0.000	1	mmu-miR-124-5p	chr14	64590670	64590691	+	miRNA	MIMAT0004527	MI0000716	CGUGUUCACAGCGGACCUUGA	mmu-miR-124-5p
12	mmu-miR-125a-5p	12.128	4477	mmu-miR-125a-5p	chr17	17830817	17830840	+	miRNA	MIMAT0000135	MI0000151	UCCCUGAGACCCUUUAACCUG	mmu-miR-125a-5p
13	mmu-miR-125b-5p	10.388	1340	mmu-miR-125b-5p	chr16	77646279	77646300	+	miRNA	MIMAT0000136	MI0000152	UCCCUGAGACCCUAACUUGUG	mmu-miR-125b-5p
14	mmu-miR-126a-5p	3.459	11	mmu-miR-126a-5p	chr2	26591365	26591385	+	miRNA	MIMAT0000137	MI0000153	CAUUAUUACUUUUGGUACGCG	mmu-miR-126a-5p
15	mmu-miR-127-5p	0.000	1	mmu-miR-127-5p	chr12	109592854	109592875	+	miRNA	MIMAT0004530	MI0000154	CUGAAGCUCAGAGGGCUCUGA	mmu-miR-127-5p
16	mmu-miR-9-5p	11.773	3499	mmu-miR-9-5p	chr13	83738821	83738843	+	miRNA	MIMAT0000142	MI0000157	UCUUUGGUUAUCUAGCUGUAU	mmu-miR-9-5p
17	mmu-miR-132-5p	3.907	15	mmu-miR-132-5p	chr11	75173686	75173707	+	miRNA	MIMAT0016984	MI0000158	AACCGUGGCUUUCGAUUGUUA	mmu-miR-132-5p
18	mmu-miR-138-5p	5.000	32	mmu-miR-138-5p	chr8	94324312	94324334	+	miRNA	MIMAT0000150	MI0000164	AGCUGGUGUUGUGAAUCAGGC	mmu-miR-138-5p
19	mmu-miR-140-5p	5.248	38	mmu-miR-140-5p	chr8	107551249	107551270	+	miRNA	MIMAT0000151	MI0000165	CAGUGGUUUUACCCUAUGGUA	mmu-miR-140-5p
20	mmu-miR-142a-5p	5.426	43	mmu-miR-142a-5p	chr11	87756867	87756887	+	miRNA	MIMAT0000154	MI0000167	CAUAAAGUAGAAAGCACUACU	mmu-miR-142a-5p
21	mmu-miR-144-5p	3.907	15	mmu-miR-144-5p	chr11	78073010	78073032	+	miRNA	MIMAT0016988	MI0000168	GGAUAUCAUCAUAUACUGUAA	mmu-miR-144-5p
22	mmu-miR-145a-5p	3.170	9	mmu-miR-145a-5p	chr18	61647866	61647888	-	miRNA	MIMAT0000157	MI0000169	GUCCAGUUUUCCCAGGAAUCC	mmu-miR-145a-5p
23	mmu-miR-146a-5p	8.033	262	mmu-miR-146a-5p	chr11	43374435	43374456	-	miRNA	MIMAT0000158	MI0000170	UGAGAACUGAAUUCCAUGGGU	mmu-miR-146a-5p

24	mmu-miR-149-5p	9.455	702	mmu-miR-149-5p	chr1	92850381	92850403	+	miRNA	MIMAT0000159	MI0000171	UCUGGCUCCGUGUCUUCACUC	mmu-miR-149-5p
25	mmu-miR-150-5p	8.290	313	mmu-miR-150-5p	chr7	45121762	45121783	+	miRNA	MIMAT0000160	MI0000172	UCUCCCAACCCUUGUACCAGU	mmu-miR-150-5p
26	mmu-miR-151-5p	3.170	9	mmu-miR-151-5p	chr15	73254855	73254875	-	miRNA	MIMAT0004536	MI0000173	UCGAGGAGCUCACAGUCUAGU	mmu-miR-151-5p
27	mmu-miR-152-5p	5.000	32	mmu-miR-152-5p	chr11	96850400	96850423	+	miRNA	MIMAT0016991	MI0000174	UAGGUUCUGUGAUACACUCCG	mmu-miR-152-5p
28	mmu-miR-153-5p	0.000	1	mmu-miR-153-5p	chr12	117250821	117250843	+	miRNA	MIMAT0016992	MI0000175	GUCAUUUUUGUGACGUUGCAG	mmu-miR-153-5p
29	mmu-miR-154-5p	1.000	2	mmu-miR-154-5p	chr12	109738438	109738459	+	miRNA	MIMAT0000164	MI0000176	UAGGUUAUCCGUGUUGCCUUC	mmu-miR-154-5p
30	mmu-miR-155-5p	5.615	49	mmu-miR-155-5p	chr16	84714143	84714165	+	miRNA	MIMAT0000165	MI0000177	UUAAUGCUAAUUGUGAUAGGG	mmu-miR-155-5p
31	mmu-miR-10b-5p	13.442	11131	mmu-miR-10b-5p	chr2	74726074	74726096	+	miRNA	MIMAT0000208	MI0000221	UACCCUGUAGAACCGAAUUUG	mmu-miR-10b-5p
32	mmu-miR-129-5p	8.804	447	mmu-miR-129-5p	chr2	94241419	94241439	-	miRNA	MIMAT0000209	MI0000585	CUUUUUGCGGUCUGGGCUUGC	mmu-miR-129-5p
33	mmu-miR-181a-5p	9.205	590	mmu-miR-181a-5p	chr1	137966468	137966490	+	miRNA	MIMAT0000210	MI0000697	AACAUUCAACGCUGUCGGUGA	mmu-miR-181a-5p
34	mmu-miR-182-5p	7.238	151	mmu-miR-182-5p	chr6	30165962	30165986	-	miRNA	MIMAT0000211	MI0000224	UUUGGCAAUGGUAGAACUCAC	mmu-miR-182-5p
35	mmu-miR-183-5p	5.954	62	mmu-miR-183-5p	chr6	30169711	30169732	-	miRNA	MIMAT0000212	MI0000225	UAUGGCACUGGUAGAAUUCAC	mmu-miR-183-5p
36	mmu-miR-185-5p	6.833	114	mmu-miR-185-5p	chr16	18327438	18327459	-	miRNA	MIMAT0000214	MI0000227	UGGAGAGAAAGGCAGUUCCUG	mmu-miR-185-5p
37	mmu-miR-186-5p	7.845	230	mmu-miR-186-5p	chr3	157544285	157544306	+	miRNA	MIMAT0000215	MI0000228	CAAAGAAUUCUCCUUUUGGGC	mmu-miR-186-5p
38	mmu-miR-187-5p	2.585	6	mmu-miR-187-5p	chr18	24429147	24429168	-	miRNA	MIMAT0016997	MI0000229	AGGCUACAACACAGGACCCGG	mmu-miR-187-5p
39	mmu-miR-188-5p	0.000	1	mmu-miR-188-5p	chrX	7248031	7248051	-	miRNA	MIMAT0000217	MI0000230	CAUCCCUUGCAUGGUGGAGGG	mmu-miR-188-5p
40	mmu-miR-24-1-5p	4.644	25	mmu-miR-24-1-5p	chr13	63301213	63301235	+	miRNA	MIMAT0000218	MI0000231	GUGCCUACUGAGCUGAUAUCA	mmu-miR-24-1-5p
41	mmu-miR-191-5p	12.075	4315	mmu-miR-191-5p	chr9	108568325	108568347	+	miRNA	MIMAT0000221	MI0000233	CAACGGAAUCCCAAAAGCAGC	mmu-miR-191-5p
42	mmu-miR-193a-5p	3.459	11	mmu-miR-193a-5p	chr11	79711975	79711996	+	miRNA	MIMAT0004544	MI0000235	UGGGUCUUUGCGGGCAAGAUG	mmu-miR-193a-5p
43	mmu-miR-194-5p	5.807	56	mmu-miR-194-5p	chr1	185313325	185313346	+	miRNA	MIMAT0000224	MI0000236	UGUAACAGCAACUCCAUGUGG	mmu-miR-194-5p
44	mmu-miR-195a-5p	3.322	10	mmu-miR-195a-5p	chr11	70235062	70235082	+	miRNA	MIMAT0000225	MI0000237	UAGCAGCACAGAAAUAUUGGC	mmu-miR-195a-5p
45	mmu-miR-199a-5p	2.585	6	mmu-miR-199a-5p	chr1	162217844	162217866	+	miRNA	MIMAT0000229	MI0000713	CCCAGUGUUCAGACUACCUGU	mmu-miR-199a-5p
46	mmu-miR-203-5p	0.000	1	mmu-miR-203-5p	chr12	112130889	112130910	+	miRNA	MIMAT0004547	MI0000246	AGUGGUUCUUGACAGUUCAAC	mmu-miR-203-5p
47	mmu-miR-204-5p	5.392	42	mmu-miR-204-5p	chr19	22750610	22750631	+	miRNA	MIMAT0000237	MI0000247	UUCCCUUUGUCAUCCUAUGCC	mmu-miR-204-5p
48	mmu-miR-205-5p	2.322	5	mmu-miR-205-5p	chr1	193507503	193507524	-	miRNA	MIMAT0000238	MI0000248	UCCUUCAUUCCACCGGAGUCU	mmu-miR-205-5p
49	mmu-miR-122-5p	13.279	9942	mmu-miR-122-5p	chr18	65248866	65248887	+	miRNA	MIMAT0000246	MI0000256	UGGAGUGUGACAAUGGUGUUU	mmu-miR-122-5p
50	mmu-miR-143-5p	2.000	4	mmu-miR-143-5p	chr18	61649233	61649253	-	miRNA	MIMAT0017006	MI0000257	GGUGCAGUGCUGCAUCUCUGG	mmu-miR-143-5p
51	mmu-miR-30e-5p	7.665	203	mmu-miR-30e-5p	chr4	120772660	120772681	-	miRNA	MIMAT0000248	MI0000259	UGUAAACAUCCUUGACUGGAA	mmu-miR-30e-5p
52	mmu-miR-297a-5p	3.170	9	mmu-miR-297a-5p	chr2	10472264	10472285	+	miRNA	MIMAT0000375	MI0000397	AUGUAUGUGUGCAUGUGCAUG	mmu-miR-297a-5p

53	mmu-miR-301a-5p	7.066	134	mmu-miR-301a-5p	chr11	87113017	87113038	+	miRNA	MIMAT0017008	MI0000401	GCUCUGACUUUAUUGCACUAC	mmu-miR-301a-5p
54	mmu-miR-34c-5p	6.807	112	mmu-miR-34c-5p	chr9	51103076	51103098	-	miRNA	MIMAT0000381	MI0000403	AGGCAGUGUAGUUAGCUGAUU	mmu-miR-34c-5p
55	mmu-miR-34b-5p	2.322	5	mmu-miR-34b-5p	chr9	51103610	51103632	-	miRNA	MIMAT0000382	MI0000404	AGGCAGUGUAAUUAGCUGAUU	mmu-miR-34b-5p
56	mmu-let-7d-5p	13.451	11201	mmu-let-7d-5p	chr13	48536078	48536099	-	miRNA	MIMAT0000383	MI0000405	AGAGGUAGUAGGUUGCAUAGU	mmu-let-7d-5p
57	mmu-miR-106b-5p	5.781	55	mmu-miR-106b-5p	chr5	138165787	138165807	-	miRNA	MIMAT0000386	MI0000407	UAAAGUGCUGACAGUGCAGAU	mmu-miR-106b-5p
58	mmu-miR-130b-5p	8.175	289	mmu-miR-130b-5p	chr16	17124109	17124130	-	miRNA	MIMAT0004583	MI0000408	ACUCUUUCCCUGUUGCACUAC	mmu-miR-130b-5p
59	mmu-miR-30c-5p	11.341	2594	mmu-miR-30c-5p	chr1	23291714	23291736	+	miRNA	MIMAT0000514	MI0000548	UGUAAACAUCCUACACUCUCA	mmu-miR-30c-5p
60	mmu-miR-30d-5p	13.000	8192	mmu-miR-30d-5p	chr15	68341257	68341278	-	miRNA	MIMAT0000515	MI0000549	UGUAAACAUCCCCGACUGGAA	mmu-miR-30d-5p
61	mmu-miR-192-5p	6.476	89	mmu-miR-192-5p	chr19	6264857	6264877	+	miRNA	MIMAT0000517	MI0000551	CUGACCUAUGAAUUGACAGCC	mmu-miR-192-5p
62	mmu-miR-196a-5p	2.322	5	mmu-miR-196a-5p	chr11	96265187	96265208	+	miRNA	MIMAT0000518	MI0000552	UAGGUAGUUUCAUGUUGUUGG	mmu-miR-196a-5p
63	mmu-let-7a-5p	15.299	40314	mmu-let-7a-5p	chr13	48538239	48538260	-	miRNA	MIMAT0000521	MI0000556	UGAGGUAGUAGGUUGUAUAGU	mmu-let-7a-5p
64	mmu-let-7b-5p	14.860	29729	mmu-let-7b-5p	chr15	85707325	85707346	+	miRNA	MIMAT0000522	MI0000558	UGAGGUAGUAGGUUGUGUGGU	mmu-let-7b-5p
65	mmu-let-7c-5p	15.827	58137	mmu-let-7c-5p	chr15	85706616	85706637	+	miRNA	MIMAT0000523	MI0000560	UGAGGUAGUAGGUUGUAUGGU	mmu-let-7c-5p
66	mmu-let-7e-5p	11.328	2570	mmu-let-7e-5p	chr17	17830366	17830387	+	miRNA	MIMAT0000524	MI0000561	UGAGGUAGGAGGUUGUAUAGU	mmu-let-7e-5p
67	mmu-let-7f-5p	14.139	18038	mmu-let-7f-5p	chr13	48537889	48537910	-	miRNA	MIMAT0000525	MI0000562	UGAGGUAGUAGAUUGUAUAGU	mmu-let-7f-5p
68	mmu-miR-15a-5p	4.322	20	mmu-miR-15a-5p	chr14	61632075	61632096	-	miRNA	MIMAT0000526	MI0000564	UAGCAGCACAUAAUGGUUUGU	mmu-miR-15a-5p
69	mmu-miR-16-5p	7.180	145	mmu-miR-16-5p	chr14	61631936	61631957	-	miRNA	MIMAT0000527	MI0000565	UAGCAGCACGUAAAUAUUGGC	mmu-miR-16-5p
70	mmu-miR-18a-5p	5.977	63	mmu-miR-18a-5p	chr14	115043867	115043889	+	miRNA	MIMAT0000528	MI0000567	UAAGGUGCAUCUAGUGCAGAU	mmu-miR-18a-5p
71	mmu-miR-20a-5p	7.600	194	mmu-miR-20a-5p	chr14	115044183	115044205	+	miRNA	MIMAT0000529	MI0000568	UAAAGUGCUUAUAGUGCAGGU	mmu-miR-20a-5p
72	mmu-miR-21a-5p	11.429	2757	mmu-miR-21a-5p	chr11	86584120	86584141	-	miRNA	MIMAT0000530	MI0000569	UAGCUUAUCAGACUGAUGUUG	mmu-miR-21a-5p
73	mmu-miR-22-5p	8.271	309	mmu-miR-22-5p	chr11	75463734	75463755	+	miRNA	MIMAT0004629	MI0000570	AGUUCUUCAGUGGCAAGCUUU	mmu-miR-22-5p
74	mmu-miR-23a-5p	5.585	48	mmu-miR-23a-5p	chr8	84208527	84208548	+	miRNA	MIMAT0017019	MI0000571	GGGGUUCCUGGGGAUGGGAUU	mmu-miR-23a-5p
75	mmu-miR-24-2-5p	8.711	419	mmu-miR-24-2-5p	chr8	84208839	84208860	+	miRNA	MIMAT0005440	MI0000572	GUGCCUACUGAGCUGAAACAG	mmu-miR-24-2-5p
76	mmu-miR-26a-5p	12.074	4311	mmu-miR-26a-5p	chr10	126995543	126995564	+	miRNA	MIMAT0000533	MI0000706	UUCAAGUAAUCCAGGAUAGGC	mmu-miR-26a-5p
77	mmu-miR-26b-5p	9.728	848	mmu-miR-26b-5p	chr1	74394324	74394344	+	miRNA	MIMAT0000534	MI0000575	UUCAAGUAAUUCAGGAUAGGU	mmu-miR-26b-5p
78	mmu-miR-27a-5p	6.229	75	mmu-miR-27a-5p	chr8	84208685	84208706	+	miRNA	MIMAT0004633	MI0000578	AGGGCUUAGCUGCUUGUGAGC	mmu-miR-27a-5p
79	mmu-miR-31-5p	7.948	247	mmu-miR-31-5p	chr4	88910614	88910635	-	miRNA	MIMAT0000538	MI0000579	AGGCAAGAUGCUGGCAUAGCU	mmu-miR-31-5p
80	mmu-miR-92a-2-5p	0.000	1	mmu-miR-92a-2-5p	chrX	52741892	52741913	-	miRNA	MIMAT0004635	MI0000580	AGGUGGGGAUUGGUGGCAUUA	mmu-miR-92a-2-5p
81	mmu-miR-93-5p	7.788	221	mmu-miR-93-5p	chr5	138165574	138165596	-	miRNA	MIMAT0000540	MI0000581	CAAAGUGCUGUUCGUGCAGGU	mmu-miR-93-5p

82	mmu-miR-96-5p	2.585	6	mmu-miR-96-5p	chr6	30169506	30169528	-	miRNA	MIMAT0000541	MI0000583	UUUGGCACUAGCACAUUUUUG	mmu-miR-96-5p
83	mmu-miR-98-5p	8.836	457	mmu-miR-98-5p	chrX	151913229	151913250	+	miRNA	MIMAT0000545	MI0000586	UGAGGUAGUAAGUUGUAUUGU	mmu-miR-98-5p
84	mmu-miR-325-5p	2.807	7	mmu-miR-325-5p	chrX	105379142	105379164	-	miRNA	MIMAT0000558	MI0000597	CCUAGUAGGUGCUCAGUAAGU	mmu-miR-325-5p
85	mmu-miR-329-5p	1.000	2	mmu-miR-329-5p	chr12	109713503	109713524	+	miRNA	MIMAT0017032	MI0000605	AGAGGUUUUCUGGGUCUCUGU	mmu-miR-329-5p
86	mmu-miR-330-5p	5.728	53	mmu-miR-330-5p	chr7	19181486	19181507	+	miRNA	MIMAT0004642	MI0000607	UCUCUGGGCCUGUGUCUUAGG	mmu-miR-330-5p
87	mmu-miR-337-5p	2.585	6	mmu-miR-337-5p	chr12	109585818	109585839	+	miRNA	MIMAT0004644	MI0000615	CGGCGUCAUGCAGGAGUUGAU	mmu-miR-337-5p
88	mmu-miR-148b-5p	5.392	42	mmu-miR-148b-5p	chr15	103285148	103285171	+	miRNA	MIMAT0017036	MI0000617	GAAGUUCUGUUAUACACUCAG	mmu-miR-148b-5p
89	mmu-miR-338-5p	7.637	199	mmu-miR-338-5p	chr11	120014816	120014837	-	miRNA	MIMAT0004647	MI0000619	AACAAUAUCCUGGUGCUGAGU	mmu-miR-338-5p
90	mmu-miR-339-5p	7.358	164	mmu-miR-339-5p	chr5	139369708	139369730	-	miRNA	MIMAT0000584	MI0000621	UCCCUGUCCUCCAGGAGCUCA	mmu-miR-339-5p
91	mmu-miR-340-5p	7.672	204	mmu-miR-340-5p	chr11	50069720	50069741	+	miRNA	MIMAT0004651	MI0000623	UUAUAAAGCAAUGAGACUGAU	mmu-miR-340-5p
92	mmu-miR-342-5p	7.827	227	mmu-miR-342-5p	chr12	108658638	108658659	+	miRNA	MIMAT0004653	MI0000627	AGGGGUGCUAUCUGUGAUUGA	mmu-miR-342-5p
93	mmu-miR-345-5p	3.585	12	mmu-miR-345-5p	chr12	108836989	108837010	+	miRNA	MIMAT0000595	MI0000632	GCUGACCCCUAGUCCAGUGCU	mmu-miR-345-5p
94	mmu-miR-350-5p	3.000	8	mmu-miR-350-5p	chr1	176772382	176772400	-	miRNA	MIMAT0017040	MI0000640	AAAGUGCAUGCGCUUUGGG	mmu-miR-350-5p
95	mmu-miR-351-5p	8.741	428	mmu-miR-351-5p	chrX	53053315	53053338	-	miRNA	MIMAT0000609	MI0000643	UCCCUGAGGAGCCCUUUGAGC	mmu-miR-351-5p
96	mmu-miR-101b-5p	1.585	3	mmu-miR-101b-5p	chr19	29135302	29135324	+	miRNA	MIMAT0017046	MI0000649	UCGGUUAUCAUGGUACCGAUG	mmu-miR-101b-5p
97	mmu-miR-10a-5p	11.243	2423	mmu-miR-10a-5p	chr11	96317186	96317208	+	miRNA	MIMAT0000648	MI0000685	UACCCUGUAGAUCCGAAUUUG	mmu-miR-10a-5p
98	mmu-miR-17-5p	8.109	276	mmu-miR-17-5p	chr14	115043684	115043706	+	miRNA	MIMAT0000649	MI0000687	CAAAGUGCUUACAGUGCAGGU	mmu-miR-17-5p
99	mmu-miR-25-5p	6.229	75	mmu-miR-25-5p	chr5	138165370	138165391	-	miRNA	MIMAT0017049	MI0000689	AGGCGGAGACUUGGGCAAUUG	mmu-miR-25-5p
100	mmu-miR-28a-5p	6.304	79	mmu-miR-28a-5p	chr16	24827868	24827889	+	miRNA	MIMAT0000653	MI0000690	AAGGAGCUCACAGUCUAUUGA	mmu-miR-28a-5p
101	mmu-miR-32-5p	5.358	41	mmu-miR-32-5p	chr4	56895272	56895293	-	miRNA	MIMAT0000654	MI0000691	UAUUGCACAUUACUAAGUUGC	mmu-miR-32-5p
102	mmu-miR-100-5p	8.794	444	mmu-miR-100-5p	chr9	41531437	41531458	+	miRNA	MIMAT0000655	MI0000692	AACCCGUAGAUCCGAACUUGU	mmu-miR-100-5p
103	mmu-miR-139-5p	7.011	129	mmu-miR-139-5p	chr7	101475382	101475403	+	miRNA	MIMAT0000656	MI0000693	UCUACAGUGCACGUGUCUCCA	mmu-miR-139-5p
104	mmu-miR-212-5p	5.833	57	mmu-miR-212-5p	chr11	75173403	75173425	+	miRNA	MIMAT0017053	MI0000696	ACCUUGGCUCUAGACUGCUUA	mmu-miR-212-5p
105	mmu-miR-214-5p	0.000	1	mmu-miR-214-5p	chr1	162223397	162223418	+	miRNA	MIMAT0004664	MI0000698	UGCCUGUCUACACUUGCUGUG	mmu-miR-214-5p
106	mmu-miR-216a-5p	4.170	18	mmu-miR-216a-5p	chr11	28757018	28757039	+	miRNA	MIMAT0000662	MI0000699	UAAUCUCAGCUGGCAACUGUG	mmu-miR-216a-5p
107	mmu-miR-218-5p	4.954	31	mmu-miR-218-5p	chr11	35616840	35616860	+	miRNA	MIMAT0000663	MI0000701	UUGUGCUUGAUCUAACCAUGU	mmu-miR-218-5p
108	mmu-miR-223-5p	2.322	5	mmu-miR-223-5p	chrX	96242842	96242864	+	miRNA	MIMAT0017056	MI0000703	CGUGUAUUUGACAAGCUGAGU	mmu-miR-223-5p
109	mmu-miR-211-5p	8.791	443	mmu-miR-211-5p	chr7	64205831	64205852	+	miRNA	MIMAT0000668	MI0000708	UUCCCUUUGUCAUCCUUUGCC	mmu-miR-211-5p
110	mmu-miR-221-5p	6.322	80	mmu-miR-221-5p	chrX	19146344	19146369	-	miRNA	MIMAT0017060	MI0000709	ACCUGGCAUACAAUGUAGAUU	mmu-miR-221-5p

111	mmu-miR-224-5p	6.629	99	mmu-miR-224-5p	chrX	72261085	72261105	-	miRNA	MIMAT0000671	MI0000711	UAAGUCACUAGUGGUUCCGUU	mmu-miR-224-5p
112	mmu-miR-199b-5p	5.087	34	mmu-miR-199b-5p	chr2	32318485	32318507	+	miRNA	MIMAT0000672	MI0000714	CCCAGUGUUUAGACUACCUGU	mmu-miR-199b-5p
113	mmu-miR-92a-1-5p	3.807	14	mmu-miR-92a-1-5p	chr14	115044437	115044459	+	miRNA	MIMAT0017066	MI0000719	AGGUUGGGAUUUGUCGCAAUG	mmu-miR-92a-1-5p
114	mmu-miR-181b-5p	10.840	1833	mmu-miR-181b-5p	chr1	137966650	137966673	+	miRNA	MIMAT0000673	MI0000723	AACAUUCAUUGCUGUCGGUGG	mmu-miR-181b-5p
115	mmu-miR-7a-5p	11.940	3928	mmu-miR-7a-5p	chr13	58392841	58392863	-	miRNA	MIMAT0000677	MI0000728	UGGAAGACUAGUGAUUUUGUU	mmu-miR-7a-5p
116	mmu-miR-7b-5p	10.625	1579	mmu-miR-7b-5p	chr17	56243017	56243040	+	miRNA	MIMAT0000678	MI0000730	UGGAAGACUUGUGAUUUUGUU	mmu-miR-7b-5p
117	mmu-miR-361-5p	5.492	45	mmu-miR-361-5p	chrX	113074867	113074888	-	miRNA	MIMAT0000704	MI0000761	UUAUCAGAAUCUCCAGGGGUA	mmu-miR-361-5p
118	mmu-miR-362-5p	0.000	1	mmu-miR-362-5p	chrX	7242019	7242042	-	miRNA	MIMAT0000706	MI0000763	AAUCCUUGGAACCUAGGUGUG	mmu-miR-362-5p
119	mmu-miR-365-1-5p	4.248	19	mmu-miR-365-1-5p	chr16	13453855	13453877	+	miRNA	MIMAT0017077	MI0000768	AGGGACUUUUGGGGGCAGAUG	mmu-miR-365-1-5p
120	mmu-miR-378a-5p	3.585	12	mmu-miR-378a-5p	chr18	61397875	61397896	-	miRNA	MIMAT0000742	MI0000795	CUCCUGACUCCAGGUCCUGUG	mmu-miR-378a-5p
121	mmu-miR-379-5p	5.555	47	mmu-miR-379-5p	chr12	109709065	109709085	+	miRNA	MIMAT0000743	MI0000796	UGGUAGACUAUGGAACGUAGG	mmu-miR-379-5p
122	mmu-miR-380-5p	0.000	1	mmu-miR-380-5p	chr12	109711806	109711827	+	miRNA	MIMAT0000744	MI0000797	AUGGUUGACCAUAGAACAUGC	mmu-miR-380-5p
123	mmu-miR-382-5p	6.044	66	mmu-miR-382-5p	chr12	109733781	109733802	+	miRNA	MIMAT0000747	MI0000799	GAAGUUGUUCGUGGUGGAUUC	mmu-miR-382-5p
124	mmu-miR-383-5p	2.585	6	mmu-miR-383-5p	chr8	38252178	38252199	-	miRNA	MIMAT0000748	MI0000800	AGAUCAGAAGGUGACUGUGGC	mmu-miR-383-5p
125	mmu-miR-335-5p	3.700	13	mmu-miR-335-5p	chr6	30741314	30741336	+	miRNA	MIMAT0000766	MI0000817	UCAAGAGCAAUAACGAAAAAU	mmu-miR-335-5p
126	mmu-miR-384-5p	2.585	6	mmu-miR-384-5p	chrX	105344332	105344354	-	miRNA	MIMAT0004745	MI0001146	UGUAAACAAUUCCUAGGCAAU	mmu-miR-384-5p
127	mmu-miR-196b-5p	7.160	143	mmu-miR-196b-5p	chr6	52230129	52230150	-	miRNA	MIMAT0001081	MI0001151	UAGGUAGUUUCCUGUUGUUGG	mmu-miR-196b-5p
128	mmu-miR-410-5p	0.000	1	mmu-miR-410-5p	chr12	109743729	109743749	+	miRNA	MIMAT0017172	MI0001161	AGGUUGUCUGUGAUGAGUUCG	mmu-miR-410-5p
129	mmu-miR-376b-5p	3.322	10	mmu-miR-376b-5p	chr12	109723471	109723492	+	miRNA	MIMAT0003388	MI0001162	GUGGAUAUUCCUUCUAUGGUU	mmu-miR-376b-5p
130	mmu-miR-411-5p	4.807	28	mmu-miR-411-5p	chr12	109710190	109710210	+	miRNA	MIMAT0004747	MI0001163	UAGUAGACCGUAUAGCGUACG	mmu-miR-411-5p
131	mmu-miR-412-5p	4.954	31	mmu-miR-412-5p	chr12	109743303	109743325	+	miRNA	MIMAT0017173	MI0001164	UGGUCGACCAGCUGGAAAGUA	mmu-miR-412-5p
132	mmu-miR-425-5p	8.033	262	mmu-miR-425-5p	chr9	108568789	108568811	+	miRNA	MIMAT0004750	MI0001447	AAUGACACGAUCACUCCCGUU	mmu-miR-425-5p
133	mmu-miR-434-5p	4.755	27	mmu-miR-434-5p	chr12	109594528	109594549	+	miRNA	MIMAT0001421	MI0001526	GCUCGACUCAUGGUUUGAACC	mmu-miR-434-5p
134	mmu-miR-365-2-5p	3.459	11	mmu-miR-365-2-5p	chr11	79726428	79726450	+	miRNA	MIMAT0017179	MI0001645	AGGGACUUUCAGGGGCAGCUG	mmu-miR-365-2-5p
135	mmu-miR-450a-5p	7.033	131	mmu-miR-450a-5p	chrX	53048206	53048227	-	miRNA	MIMAT0001546	MI0001653	UUUUGCGAUGUGUUCCUAAUA	mmu-miR-450a-5p
136	mmu-miR-451a	11.005	2055	mmu-miR-451a	chr11	78073186	78073207	+	miRNA	MIMAT0001632	MI0001730	AAACCGUUACCAUUACUGAGU	mmu-miR-451a
137	mmu-miR-452-5p	3.700	13	mmu-miR-452-5p	chrX	72262271	72262292	-	miRNA	MIMAT0001637	MI0001734	UGUUUGCAGAGGAAACUGAGA	mmu-miR-452-5p
138	mmu-miR-470-5p	2.585	6	mmu-miR-470-5p	chrX	66813995	66814017	-	miRNA	MIMAT0002111	MI0002405	UUCUUGGACUGGCACUGGUGA	mmu-miR-470-5p
139	mmu-miR-532-5p	7.807	224	mmu-miR-532-5p	chrX	7248456	7248477	-	miRNA	MIMAT0002889	MI0003206	CAUGCCUUGAGUGUAGGACCG	mmu-miR-532-5p

140	mmu-miR-484	8.972	502	mmu-miR-484	chr16	14159630	14159651	+	miRNA	MIMAT0003127	MI0003491	UCAGGCUCAGUCCCCUCCCGAU	mmu-miR-484
141	mmu-miR-485-5p	1.585	3	mmu-miR-485-5p	chr12	109734910	109734931	+	miRNA	MIMAT0003128	MI0003492	AGAGGCUGGCCGUGAUGAAUU	mmu-miR-485-5p
142	mmu-miR-486a-5p	9.905	959	mmu-miR-486a-5p	chr8	23142587	23142608	+	miRNA	MIMAT0003130	MI0003493	UCCUGUACUGAGCUGCCCCGA	mmu-miR-486a-5p
143	mmu-miR-539-5p	0.000	1	mmu-miR-539-5p	chr12	109728136	109728157	+	miRNA	MIMAT0003169	MI0003520	GGAGAAAUUAUCCUUGGUGUG	mmu-miR-539-5p
144	mmu-miR-541-5p	6.524	92	mmu-miR-541-5p	chr12	109742422	109742446	+	miRNA	MIMAT0003170	MI0003521	AAGGGAUUCUGAUGUUGGUCA	mmu-miR-541-5p
145	mmu-miR-542-5p	3.000	8	mmu-miR-542-5p	chrX	53049453	53049474	-	miRNA	MIMAT0003171	MI0003522	CUCGGGGAUCAUCAUGUCACG	mmu-miR-542-5p
146	mmu-miR-487b-5p	1.585	3	mmu-miR-487b-5p	chr12	109727347	109727367	+	miRNA	MIMAT0017216	MI0003534	UGGUUAUCCCUGUCCUCUUCG	mmu-miR-487b-5p
147	mmu-miR-369-5p	1.000	2	mmu-miR-369-5p	chr12	109743431	109743452	+	miRNA	MIMAT0003185	MI0003535	AGAUCGACCGUGUUAUAUUCG	mmu-miR-369-5p
148	mmu-miR-20b-5p	0.000	1	mmu-miR-20b-5p	chrX	52742159	52742181	-	miRNA	MIMAT0003187	MI0003536	CAAAGUGCUCAUAGUGCAGGU	mmu-miR-20b-5p
149	mmu-miR-503-5p	6.340	81	mmu-miR-503-5p	chrX	53054027	53054049	-	miRNA	MIMAT0003188	MI0003538	UAGCAGCGGGAACAGUACUGC	mmu-miR-503-5p
150	mmu-miR-1224-5p	2.000	4	mmu-miR-1224-5p	chr16	20604452	20604472	+	miRNA	MIMAT0005460	MI0004118	GUGAGGACUGGGGAGGUGGAG	mmu-miR-1224-5p
151	mmu-miR-301b-5p	1.000	2	mmu-miR-301b-5p	chr16	17124456	17124477	-	miRNA	MIMAT0017232	MI0004122	GCUCUGACUAGGUUGCACUAC	mmu-miR-301b-5p
152	mmu-miR-744-5p	12.821	7234	mmu-miR-744-5p	chr11	65734799	65734820	-	miRNA	MIMAT0004187	MI0004124	UGCGGGGCUAGGGCUAACAGC	mmu-miR-744-5p
153	mmu-miR-374b-5p	6.570	95	mmu-miR-374b-5p	chrX	103573112	103573133	-	miRNA	MIMAT0003727	MI0004125	AUAUAAUACAACCUGCUAAGU	mmu-miR-374b-5p
154	mmu-miR-1249-5p	5.672	51	mmu-miR-1249-5p	chr15	84951579	84951603	-	miRNA	MIMAT0014804	MI0004132	AGGAGGGAGGGGAUGGGCCAA	mmu-miR-1249-5p
155	mmu-miR-671-5p	3.807	14	mmu-miR-671-5p	chr5	24592132	24592154	+	miRNA	MIMAT0003731	MI0004133	AGGAAGCCCUGGAGGGGCUGG	mmu-miR-671-5p
156	mmu-miR-1843a-5p	5.209	37	mmu-miR-1843a-5p	chr12	80391654	80391674	-	miRNA	MIMAT0014805	MI0004155	UAUGGAGGUCUCUGUCUGACU	mmu-miR-1843a-5p
157	mmu-miR-344d-3-5p	0.000	1	mmu-miR-344d-3-5p	chr7	61726296	61726318	-	miRNA	MIMAT0014807	MI0004227	AGUCAGGCUAGUGGUUAUACU	mmu-miR-344d-3-5p
158	mmu-miR-1298-5p	4.907	30	mmu-miR-1298-5p	chrX	147064918	147064939	+	miRNA	MIMAT0014809	MI0004300	UUCAUUCGGCUGUCCAGAUGU	mmu-miR-1298-5p
159	mmu-miR-3099-5p	1.585	3	mmu-miR-3099-5p	chr7	6803600	6803620	+	miRNA	MIMAT0014815	MI0004485	CCAGCUUCCUUCCAGCCCUUG	mmu-miR-3099-5p
160	mmu-miR-3106-5p	2.000	4	mmu-miR-3106-5p	chr8	16168805	16168825	-	miRNA	MIMAT0014817	MI0004486	UGGCUCAUUUAGAAGCAGCCA	mmu-miR-3106-5p
161	mmu-miR-666-5p	0.000	1	mmu-miR-666-5p	chr12	109717103	109717124	+	miRNA	MIMAT0003737	MI0004553	AGCGGGCACAGCUGUGAGAGC	mmu-miR-666-5p
162	mmu-miR-674-5p	3.322	10	mmu-miR-674-5p	chr2	117185151	117185172	+	miRNA	MIMAT0003740	MI0004611	GCACUGAGAUGGGAGUGGUGU	mmu-miR-674-5p
163	mmu-miR-677-5p	0.000	1	mmu-miR-677-5p	chr10	128085291	128085312	+	miRNA	MIMAT0003451	MI0004634	UUCAGUGAUGAUUAGCUUCUG	mmu-miR-677-5p
164	mmu-miR-678	0.000	1	mmu-miR-678	chr10	76207333	76207354	-	miRNA	MIMAT0003452	MI0004635	GUCUCGGUGCAAGGACUGGAG	mmu-miR-678
165	mmu-miR-423-5p	11.943	3937	mmu-miR-423-5p	chr11	77078122	77078144	-	miRNA	MIMAT0004825	MI0004637	UGAGGGGCAGAGAGCGAGACU	mmu-miR-423-5p
166	mmu-miR-690	6.954	124	mmu-miR-690	chr16	28599940	28599961	-	miRNA	MIMAT0003469	MI0004658	AAAGGCUAGGCUCACAACCAA	mmu-miR-690
167	mmu-miR-692	6.539	93	mmu-miR-692	chr13	74407121	74407141	+	miRNA	MIMAT0003471	MI0023541	AUCUCUUUGAGCGCCUCACUC	mmu-miR-692
168	mmu-miR-146b-5p	7.160	143	mmu-miR-146b-5p	chr19	46342790	46342811	+	miRNA	MIMAT0003475	MI0004665	UGAGAACUGAAUUCCAUAGGC	mmu-miR-146b-5p

169	mmu-miR-669c-5p	0.000	1	mmu-miR-669c-5p	chr2	10509319	10509340	+	miRNA	MIMAT0003479	MI0004673	AUAGUUGUGUGUGGAUGUGUG	mmu-miR-669c-5p
170	mmu-miR-695	1.000	2	mmu-miR-695	chr2	155356831	155356852	+	miRNA	MIMAT0003481	MI0004675	AGAUUGGGCAUAGGUGACUGA	mmu-miR-695
171	mmu-miR-499-5p	0.000	1	mmu-miR-499-5p	chr2	155622893	155622913	+	miRNA	MIMAT0003482	MI0004676	UUAAGACUUGCAGUGAUGUUU	mmu-miR-499-5p
172	mmu-miR-455-5p	2.322	5	mmu-miR-455-5p	chr4	63256867	63256888	+	miRNA	MIMAT0003485	MI0004679	UAUGUGCCUUUGGACUACAUC	mmu-miR-455-5p
173	mmu-miR-698-5p	1.000	2	mmu-miR-698-5p	chr4	124743818	124743838	+	miRNA	MIMAT0022930	MI0004682	UGUGGGUGGGACAGGGAUGUU	mmu-miR-698-5p
174	mmu-miR-700-5p	2.807	7	mmu-miR-700-5p	chr4	135416601	135416622	-	miRNA	MIMAT0017256	MI0004684	UAAGGCUCCUUCCUGUGCUUG	mmu-miR-700-5p
175	mmu-miR-701-5p	0.000	1	mmu-miR-701-5p	chr5	111004170	111004190	+	miRNA	MIMAT0003491	MI0004685	UUAGCCGCUGAAAUAGAUGGA	mmu-miR-701-5p
176	mmu-miR-702-5p	4.392	21	mmu-miR-702-5p	chr5	136991442	136991462	+	miRNA	MIMAT0022931	MI0004686	GUGAGUGGGGUGGUUGGCAUG	mmu-miR-702-5p
177	mmu-miR-704	4.087	17	mmu-miR-704	chr6	47803590	47803610	-	miRNA	MIMAT0003494	MI0004688	AGACAUGUGCUCUGCUCCUAG	mmu-miR-704
178	mmu-miR-705	1.000	2	mmu-miR-705	chr6	85336341	85336360	-	miRNA	MIMAT0003495	MI0004689	GGUGGGAGGUGGGGUGGGCA	mmu-miR-705
179	mmu-miR-706	4.585	24	mmu-miR-706	chr6	120034290	120034311	-	miRNA	MIMAT0003496	MI0004690	AGAGAAACCCUGUCUCAAAAA	mmu-miR-706
180	mmu-miR-708-5p	2.000	4	mmu-miR-708-5p	chr7	96249450	96249472	+	miRNA	MIMAT0004828	MI0004692	AAGGAGCUUACAAUCUAGCUG	mmu-miR-708-5p
181	mmu-miR-709	7.033	131	mmu-miR-709	chr8	84086167	84086185	+	miRNA	MIMAT0003499	MI0004693	GGAGGCAGAGGCAGGAGGA	mmu-miR-709
182	mmu-miR-712-5p	2.322	5	mmu-miR-712-5p	.	.	.	.	.	.	.	CUCCUUCACCCGGGCGGUACC	mmu-miR-712-5p
183	mmu-miR-501-5p	0.000	1	mmu-miR-501-5p	chrX	7241307	7241328	-	miRNA	MIMAT0003508	MI0004703	AAUCCUUUGUCCCUGGGUGAA	mmu-miR-501-5p
184	mmu-miR-450b-5p	4.524	23	mmu-miR-450b-5p	chrX	53048044	53048065	-	miRNA	MIMAT0003511	MI0004705	UUUUGCAGUAUGUUCCUGAAU	mmu-miR-450b-5p
185	mmu-miR-505-5p	6.443	87	mmu-miR-505-5p	chrX	60394451	60394473	-	miRNA	MIMAT0017259	MI0004706	GGGAGCCAGGAAGUAUUGAUG	mmu-miR-505-5p
186	mmu-miR-652-5p	2.000	4	mmu-miR-652-5p	chrX	142739020	142739043	+	miRNA	MIMAT0017260	MI0004965	CAACCCUAGGAGGGGGUGCCA	mmu-miR-652-5p
187	mmu-miR-804	0.000	1	mmu-miR-804	chr11	50357799	50357820	-	miRNA	MIMAT0004210	MI0005203	UGUGAGUUGUUCCUCACCUGG	mmu-miR-804
188	mmu-miR-743a-5p	0.000	1	mmu-miR-743a-5p	chrX	66776794	66776815	-	miRNA	MIMAT0017263	MI0005207	UAUUCAGAUUGGUGCCUGUCA	mmu-miR-743a-5p
189	mmu-miR-181d-5p	5.883	59	mmu-miR-181d-5p	chr8	84178759	84178781	-	miRNA	MIMAT0004324	MI0005450	AACAUUCAUUGUUGUCGGUGG	mmu-miR-181d-5p
190	mmu-miR-743b-5p	2.322	5	mmu-miR-743b-5p	chrX	66777302	66777322	-	miRNA	MIMAT0004839	MI0005470	UGUUCAGACUGGUGUCCAUCA	mmu-miR-743b-5p
191	mmu-miR-871-5p	0.000	1	mmu-miR-871-5p	chrX	66810472	66810494	-	miRNA	MIMAT0004841	MI0005471	UAUUCAGAUUAGUGCCAGUCA	mmu-miR-871-5p
192	mmu-miR-883a-5p	0.000	1	mmu-miR-883a-5p	chrX	66780803	66780824	-	miRNA	MIMAT0004848	MI0005476	UGCUGAGAGAAGUAGCAGUUA	mmu-miR-883a-5p
193	mmu-miR-190b-5p	4.459	22	mmu-miR-190b-5p	chr3	90070030	90070051	+	miRNA	MIMAT0004852	MI0005478	UGAUAUGUUUGAUAUUGGGUU	mmu-miR-190b-5p
194	mmu-miR-18b-5p	1.000	2	mmu-miR-18b-5p	chrX	52742381	52742403	-	miRNA	MIMAT0004858	MI0005483	UAAGGUGCAUCUAGUGCUGUU	mmu-miR-18b-5p
195	mmu-miR-193b-5p	1.585	3	mmu-miR-193b-5p	chr16	13449533	13449554	+	miRNA	MIMAT0017271	MI0005484	CGGGGUUUUGAGGGCGAGAUG	mmu-miR-193b-5p
196	mmu-miR-465b-5p	3.322	10	mmu-miR-465b-5p	chrX	66829249	66829270	-	miRNA	MIMAT0004871	MI0005498	UAUUUAGAAUGGUGCUGAUCU	mmu-miR-465b-5p
197	mmu-miR-465c-5p	2.322	5	mmu-miR-465c-5p	chrX	66826004	66826025	-	miRNA	MIMAT0004873	MI0005500	UAUUUAGAAUGGCGCUGAUCU	mmu-miR-465c-5p

198	mmu-miR-466f-5p	0.000	1	mmu-miR-466f-5p	chr2	10466954	10466975	+	miRNA	MIMAT0004881	MI0005507	UACGUGUGUGUGCAUGUGCAU	mmu-miR-466f-5p
199	mmu-miR-466g	0.000	1	mmu-miR-466g	chr2	10514642	10514662	+	miRNA	MIMAT0004883	MI0005510	AUACAGACACAUGCACACACA	mmu-miR-466g
200	mmu-miR-493-5p	3.807	14	mmu-miR-493-5p	chr12	109580243	109580261	+	miRNA	MIMAT0017276	MI0005514	UUGUACAUGGUAGGCUUUC	mmu-miR-493-5p
201	mmu-miR-504-5p	1.585	3	mmu-miR-504-5p	chrX	59097705	59097726	-	miRNA	MIMAT0004889	MI0005515	AGACCCUGGUCUGCACUCUAU	mmu-miR-504-5p
202	mmu-miR-574-5p	6.392	84	mmu-miR-574-5p	chr5	64970328	64970350	+	miRNA	MIMAT0004893	MI0005518	UGAGUGUGUGUGUGUGAGUGU	mmu-miR-574-5p
203	mmu-miR-92b-5p	2.322	5	mmu-miR-92b-5p	chr3	89227165	89227188	-	miRNA	MIMAT0017278	MI0005521	AGGGACGGGACGUGGUGCAGU	mmu-miR-92b-5p
204	mmu-miR-878-5p	0.000	1	mmu-miR-878-5p	chrX	66801554	66801575	-	miRNA	MIMAT0004932	MI0005548	UAUCUAGUUGGAUGUCAAGAC	mmu-miR-878-5p
205	mmu-miR-872-5p	6.248	76	mmu-miR-872-5p	chr4	94665167	94665187	+	miRNA	MIMAT0004934	MI0005549	AAGGUUACUUGUUAGUUCAGG	mmu-miR-872-5p
206	mmu-miR-877-5p	3.907	15	mmu-miR-877-5p	chr17	35960795	35960814	-	miRNA	MIMAT0004861	MI0005553	GUAGAGGAGAUGGCGCAGGG	mmu-miR-877-5p
207	mmu-miR-582-5p	0.000	1	mmu-miR-582-5p	chr13	109324753	109324774	+	miRNA	MIMAT0005291	MI0006127	AUACAGUUGUUCAACCAGUUA	mmu-miR-582-5p
208	mmu-miR-466i-5p	4.954	31	mmu-miR-466i-5p	chr13	17747525	17747544	+	miRNA	MIMAT0017325	MI0006282	UGUGUGUGUGUGUGUGUG	mmu-miR-466i-5p
209	mmu-miR-1b-5p	3.322	10	mmu-miR-1b-5p	chr18	10785486	10785506	+	miRNA	MIMAT0005835	MI0006283	UACAUACUUCUUUACAUUCCA	mmu-miR-1b-5p
210	mmu-miR-1187	4.087	17	mmu-miR-1187	chr5	82798964	82798986	-	miRNA	MIMAT0005837	MI0006285	UAUGUGUGUGUGUAUGUGUGU	mmu-miR-1187
211	mmu-miR-467f	1.000	2	mmu-miR-467f	chr11	69635419	69635439	-	miRNA	MIMAT0005846	MI0006293	AUAUACACACACACACCUACA	mmu-miR-467f
212	mmu-miR-1190	0.000	1	mmu-miR-1190	chr12	101021692	101021713	-	miRNA	MIMAT0005847	MI0006294	UCAGCUGAGGUUCCCCUCUGU	mmu-miR-1190
213	mmu-miR-1191a	6.858	116	mmu-miR-1191a	chr7	27205615	27205635	+	miRNA	MIMAT0005849	MI0006296	CAGUCUUACUAUGUAGCCCUA	mmu-miR-1191a
214	mmu-miR-1194	1.585	3	mmu-miR-1194	.	.	.	.	.	.	.	GAAUGAGUAACUGCUAGAUCC	mmu-miR-1194
215	mmu-miR-467g	1.000	2	mmu-miR-467g	chr5	34732872	34732892	-	miRNA	MIMAT0005854	MI0006301	UAUACAUACACACACAUAUAU	mmu-miR-467g
216	mmu-miR-1195	7.827	227	mmu-miR-1195	chr17	70860558	70860580	-	miRNA	MIMAT0005856	MI0006303	UGAGUUCGAGGCCAGCCUGCU	mmu-miR-1195
217	mmu-miR-1198-5p	9.533	741	mmu-miR-1198-5p	chrX	7807142	7807163	+	miRNA	MIMAT0005859	MI0006306	UAUGUGUUCCUGGCUGGCUUG	mmu-miR-1198-5p
218	mmu-miR-1902	1.000	2	mmu-miR-1902	chr2	104428871	104428892	-	miRNA	MIMAT0007863	MI0008313	AGAGGUGCAGUAGGCAUGACU	mmu-miR-1902
219	mmu-miR-1895	1.585	3	mmu-miR-1895	chr3	134240515	134240536	-	miRNA	MIMAT0007867	MI0008316	CCCCCGAGGAGGACGAGGAGG	mmu-miR-1895
220	mmu-miR-1903	2.322	5	mmu-miR-1903	chr8	128359251	128359272	+	miRNA	MIMAT0007868	MI0008317	CCUUCUUCUUCUUCCUGAGAC	mmu-miR-1903
221	mmu-miR-1892	2.322	5	mmu-miR-1892	chr12	54645943	54645964	-	miRNA	MIMAT0007871	MI0008320	AUUUGGGGACGGGAGGGAGGA	mmu-miR-1892
222	mmu-miR-1904	0.000	1	mmu-miR-1904	chr13	109903819	109903840	+	miRNA	MIMAT0007874	MI0008323	GUUCUGCUCCUCUGGAGGGAG	mmu-miR-1904
223	mmu-miR-1894-5p	0.000	1	mmu-miR-1894-5p	chr17	35917899	35917920	+	miRNA	MIMAT0007877	MI0008326	CUCUCCCCUACCACCUGCCUCU	mmu-miR-1894-5p
224	mmu-miR-1929-5p	0.000	1	mmu-miR-1929-5p	chr10	44359692	44359714	+	miRNA	MIMAT0009392	MI0009918	UUCUAGGACUUUAUAGAGCAG	mmu-miR-1929-5p
225	mmu-miR-1931	1.585	3	mmu-miR-1931	chr10	93162879	93162900	+	miRNA	MIMAT0009394	MI0009920	AUGCAAGGGCUGGUGCGAUGG	mmu-miR-1931
226	mmu-miR-1933-5p	4.392	21	mmu-miR-1933-5p	chr11	21344640	21344663	-	miRNA	MIMAT0009396	MI0009922	AGUCAUGGUGUUCGGUCUUAG	mmu-miR-1933-5p

227	mmu-miR-1934-5p	1.000	2	mmu-miR-1934-5p	chr11	69663055	69663077	+	miRNA	MIMAT0009398	MI0009923	UCUGGUCCCCUGCUUCGUCCU	mmu-miR-1934-5p
228	mmu-miR-1941-5p	3.459	11	mmu-miR-1941-5p	chr15	101369364	101369387	+	miRNA	MIMAT0009405	MI0009930	AGGGAGAUGCUGGUACAGAGG	mmu-miR-1941-5p
229	mmu-miR-1943-5p	3.170	9	mmu-miR-1943-5p	chr15	79375271	79375293	-	miRNA	MIMAT0009408	MI0009932	AAGGGAGGAUCUGGGCACCUG	mmu-miR-1943-5p
230	mmu-miR-1945	2.322	5	mmu-miR-1945	chr16	11254381	11254402	-	miRNA	MIMAT0009410	MI0009934	UCUUCGCGGGUACUGUCGGGA	mmu-miR-1945
231	mmu-miR-1306-5p	1.585	3	mmu-miR-1306-5p	chr16	18284288	18284310	-	miRNA	MIMAT0019136	MI0009935	CACCACCUCCCCUGCAAACGU	mmu-miR-1306-5p
232	mmu-miR-1946a	5.170	36	mmu-miR-1946a	chr16	32267540	32267566	-	miRNA	MIMAT0009412	MI0009936	AGCCGGGCAGUGGUGGCACAC	mmu-miR-1946a
233	mmu-miR-1947-5p	6.229	75	mmu-miR-1947-5p	chr16	33105375	33105396	+	miRNA	MIMAT0009413	MI0009937	AGGACGAGCUAGCUGAGUGCU	mmu-miR-1947-5p
234	mmu-miR-1951	0.000	1	mmu-miR-1951	chr2	115638777	115638798	+	miRNA	MIMAT0009422	MI0009946	GUAGUGGAGACUGGUGUGGCU	mmu-miR-1951
235	mmu-miR-1953	1.585	3	mmu-miR-1953	chr2	151967581	151967602	-	miRNA	MIMAT0009424	MI0009948	UGGGAAAGUUCUCAGGCUUCU	mmu-miR-1953
236	mmu-miR-1960	2.000	4	mmu-miR-1960	chr5	30170751	30170772	+	miRNA	MIMAT0009433	MI0009957	CCAGUGCUGUUAGAAGAGGGC	mmu-miR-1960
237	mmu-miR-1961	4.954	31	mmu-miR-1961	chr5	92788465	92788481	-	miRNA	MIMAT0009434	MI0009958	UGAGGUAGUAGUUAGAA	mmu-miR-1961
238	mmu-miR-1969	2.585	6	mmu-miR-1969	chr8	70925541	70925563	+	miRNA	MIMAT0009442	MI0009966	AAGAUGGAGACUUUAACAUGG	mmu-miR-1969
239	mmu-miR-1946b	3.459	11	mmu-miR-1946b	chr9	21613452	21613477	-	miRNA	MIMAT0009443	MI0009967	GCCGGGCAGUGGUGGCACAUG	mmu-miR-1946b
240	mmu-miR-1971	1.585	3	mmu-miR-1971	chr14	78191451	78191468	-	miRNA	MIMAT0009446	MI0009970	GUAAAGGCUGGGCUGAGA	mmu-miR-1971
241	mmu-miR-1983	4.248	19	mmu-miR-1983	chr13	21896930	21896950	-	miRNA	MIMAT0009455	MI0009990	CUCACCUGGAGCAUGUUUUCU	mmu-miR-1983
242	mmu-miR-1839-5p	7.468	177	mmu-miR-1839-5p	chr7	81529919	81529940	+	miRNA	MIMAT0009456	MI0009991	AAGGUAGAUAGAACAGGUCUU	mmu-miR-1839-5p
243	mmu-miR-1981-5p	9.516	732	mmu-miR-1981-5p	chr1	184822465	184822488	-	miRNA	MIMAT0009458	MI0009992	GUAAAGGCUGGGCUUAGACGU	mmu-miR-1981-5p
244	mmu-miR-2137	5.883	59	mmu-miR-2137	chrX	72992113	72992133	+	miRNA	MIMAT0011213	MI0010750	GCCGGCGGGAGCCCCAGGGAG	mmu-miR-2137
245	mmu-miR-2183	1.585	3	mmu-miR-2183	.	.	.	.	.	.	.	UUGAACCCCUGACCUCCU	mmu-miR-2183
246	mmu-miR-664-5p	2.807	7	mmu-miR-664-5p	chr1	185242975	185242995	+	miRNA	MIMAT0017353	MI0012531	CUGGCUGGGGAAAAUGACUGG	mmu-miR-664-5p
247	mmu-miR-3057-5p	6.476	89	mmu-miR-3057-5p	chr10	81271610	81271633	+	miRNA	MIMAT0014822	MI0014020	AUUGGAGCUGAGAUUCUGCGG	mmu-miR-3057-5p
248	mmu-miR-3061-5p	4.700	26	mmu-miR-3061-5p	chr11	52126759	52126780	+	miRNA	MIMAT0014828	MI0014023	CAGUGGGCCGUGAAAGGUAGC	mmu-miR-3061-5p
249	mmu-miR-3062-5p	0.000	1	mmu-miR-3062-5p	chr11	68990638	68990659	-	miRNA	MIMAT0014830	MI0014024	GGAGAAUGUAGUGUUACCGUG	mmu-miR-3062-5p
250	mmu-miR-3064-5p	2.807	7	mmu-miR-3064-5p	chr11	106782736	106782757	-	miRNA	MIMAT0014834	MI0014026	UCUGGCUGUUGUGGUGUGCAA	mmu-miR-3064-5p
251	mmu-miR-3066-5p	4.700	26	mmu-miR-3066-5p	chr12	17355404	17355425	+	miRNA	MIMAT0014838	MI0014028	UUGGUUGCUGUAGAUUAAGUA	mmu-miR-3066-5p
252	mmu-miR-3068-5p	3.000	8	mmu-miR-3068-5p	chr12	87437724	87437747	-	miRNA	MIMAT0014842	MI0014030	UUGGAGUUCAUGCAAGUUCUA	mmu-miR-3068-5p
253	mmu-miR-3074-5p	7.852	231	mmu-miR-3074-5p	chr13	63301249	63301270	-	miRNA	MIMAT0014856	MI0014037	GUUCCUGCUGAACUGAGCCAG	mmu-miR-3074-5p
254	mmu-miR-3079-5p	2.000	4	mmu-miR-3079-5p	chr15	76290932	76290954	-	miRNA	MIMAT0014866	MI0014042	UUUGAUCUGAUGAGCUAAGCU	mmu-miR-3079-5p
255	mmu-miR-3081-5p	0.000	1	mmu-miR-3081-5p	chr16	44558092	44558114	-	miRNA	MIMAT0014870	MI0014044	GACUGGAGCUUGGAGCGGUGA	mmu-miR-3081-5p

256	mmu-miR-3082-5p	4.322	20	mmu-miR-3082-5p	chr17	25831406	25831427	-	miRNA	MIMAT0014872	MI0014045	GACAGAGUGUGUGUGUCUGUG	mmu-miR-3082-5p
257	mmu-miR-3090-5p	1.000	2	mmu-miR-3090-5p	chr2	133564722	133564742	+	miRNA	MIMAT0014901	MI0014083	GUCUGGGUGGGGCCUGAGAUC	mmu-miR-3090-5p
258	mmu-miR-3094-5p	1.000	2	mmu-miR-3094-5p	chr4	40993696	40993716	+	miRNA	MIMAT0014909	MI0014087	UGUUGGGGACAUUUUUAAAGC	mmu-miR-3094-5p
259	mmu-miR-3100-5p	3.459	11	mmu-miR-3100-5p	chr7	19086828	19086850	+	miRNA	MIMAT0014919	MI0014092	UUGGGAACGGGGUGUCUUUGG	mmu-miR-3100-5p
260	mmu-miR-344c-5p	3.170	9	mmu-miR-344c-5p	chr7	61837363	61837385	-	miRNA	MIMAT0014927	MI0014096	AGUCAGGCUGCUGGCUAGAGU	mmu-miR-344c-5p
261	mmu-miR-486b-5p	10.010	1031	mmu-miR-486b-5p	chr8	23142627	23142648	-	miRNA	MIMAT0014943	MI0014103	UCCUGUACUGAGCUGCCCCGA	mmu-miR-486b-5p
262	mmu-miR-374c-5p	1.000	2	mmu-miR-374c-5p	chrX	103573086	103573105	+	miRNA	MIMAT0014953	MI0014108	AUAAUACAACCUGCUAAGUG	mmu-miR-374c-5p
263	mmu-miR-3470a	5.700	52	mmu-miR-3470a	chr6	83090360	83090380	-	miRNA	MIMAT0015640	MI0014696	UCACUUUGUAGACCAGGCUGG	mmu-miR-3470a
264	mmu-miR-3470b	5.954	62	mmu-miR-3470b	chr16	44013885	44013905	+	miRNA	MIMAT0015641	MI0014697	UCACUCUGUAGACCAGGCUGG	mmu-miR-3470b
265	mmu-miR-3472	0.000	1	mmu-miR-3472	chrX	145039390	145039414	+	miRNA	MIMAT0015643	MI0014704	UAAUAGCCAGAAGCUGGAAGG	mmu-miR-3472
266	mmu-miR-3473a	6.358	82	mmu-miR-3473a	chrX	162874920	162874937	-	miRNA	MIMAT0015645	MI0014706	UGGAGAGAUGGCUCAGCA	mmu-miR-3473a
267	mmu-miR-3960	2.000	4	mmu-miR-3960	chr2	32712907	32712926	-	miRNA	MIMAT0019336	MI0016963	GGCGGCGGCGGAGGCGGGGG	mmu-miR-3960
268	mmu-miR-28c	1.000	2	mmu-miR-28c	chr15	53614216	53614235	-	miRNA	MIMAT0019339	MI0016966	AGGAGCUCACAGUCUAUUGA	mmu-miR-28c
269	mmu-miR-3962	6.644	100	mmu-miR-3962	chr1	194822035	194822054	+	miRNA	MIMAT0019340	MI0016967	AGGUAGUAGUUUGUACAUUU	mmu-miR-3962
270	mmu-miR-3963	6.768	109	mmu-miR-3963	chr3	151023839	151023857	-	miRNA	MIMAT0019341	MI0016968	UGUAUCCCACUUCUGACAC	mmu-miR-3963
271	mmu-miR-3964	1.000	2	mmu-miR-3964	chr15	29713337	29713356	-	miRNA	MIMAT0019344	MI0016970	AUAAGGUAGAAAGCACUAAA	mmu-miR-3964
272	mmu-miR-1843b-5p	5.954	62	mmu-miR-1843b-5p	chr1	159340362	159340382	+	miRNA	MIMAT0019345	MI0016971	AUGGAGGUCUCUGUCUGACUU	mmu-miR-1843b-5p
273	mmu-miR-3965	1.000	2	mmu-miR-3965	chr5	134326494	134326512	+	miRNA	MIMAT0019347	MI0016972	UGCUUAUCAGCCUGAUGUU	mmu-miR-3965
274	mmu-miR-378b	6.392	84	mmu-miR-378b	chr11	88352839	88352858	+	miRNA	MIMAT0019348	MI0016973	CUGGACUUGGAGUCAGAAGA	mmu-miR-378b
275	mmu-miR-101c	1.585	3	mmu-miR-101c	chr9	3038680	3038698	-	miRNA	MIMAT0019349	MI0016974	ACAGUACUGUGAUAACUGA	mmu-miR-101c
276	mmu-miR-3968	6.570	95	mmu-miR-3968	chr11	115447969	115447989	-	miRNA	MIMAT0019352	MI0016977	CGAAUCCCACUCCAGACACCA	mmu-miR-3968
277	mmu-miR-3969	0.000	1	mmu-miR-3969	chr5	87595963	87595982	-	miRNA	MIMAT0019353	MI0016978	CCCUAAAGUAGAAAUCACUA	mmu-miR-3969
278	mmu-miR-3970	2.000	4	mmu-miR-3970	chr19	33157122	33157141	+	miRNA	MIMAT0019355	MI0016980	GAGGUUGUAGUUUGUGCUUU	mmu-miR-3970
279	mmu-miR-3473b	10.262	1228	mmu-miR-3473b	chr10	41670774	41670793	+	miRNA	MIMAT0020367	MI0016997	GGGCUGGAGAGAUGGCUCAG	mmu-miR-3473b
280	mmu-miR-5099	16.000	65554	mmu-miR-5099	chr12	36816250	36816269	+	miRNA	MIMAT0020606	MI0018007	UUAGAUCGAUGUGGUGCUCC	mmu-miR-5099
281	mmu-miR-5100	4.858	29	mmu-miR-5100	chr11	60728698	60728718	+	miRNA	MIMAT0020607	MI0018008	UCGAAUCCCAGCGGUGCCUCU	mmu-miR-5100
282	mmu-miR-5106	7.476	178	mmu-miR-5106	chr4	44221197	44221219	-	miRNA	MIMAT0020613	MI0018014	AGGUCUGUAGCUCAGUUGGCA	mmu-miR-5106
283	mmu-miR-5107-5p	0.000	1	mmu-miR-5107-5p	chr18	60812086	60812106	+	miRNA	MIMAT0020615	MI0018016	UGGGCAGAGGAGGCAGGGACA	mmu-miR-5107-5p
284	mmu-miR-5108	4.954	31	mmu-miR-5108	chr10	61774795	61774813	+	miRNA	MIMAT0020616	MI0018017	GUAGAGCACUGGAUGGUUU	mmu-miR-5108

285	mmu-miR-5110	3.700	13	mmu-miR-5110	chr11	85760675	85760699	-	miRNA	MIMAT0020618	MI0018019	GGAGGAGGUAGAGGGUGGUGG	mmu-miR-5110
286	mmu-miR-5112	3.459	11	mmu-miR-5112	chr18	82720291	82720309	+	miRNA	MIMAT0020620	MI0018021	UAGCUCAGCGGGAGAGCAC	mmu-miR-5112
287	mmu-miR-5114	3.170	9	mmu-miR-5114	chr19	44303171	44303192	+	miRNA	MIMAT0020622	MI0018023	ACUGGAGACGGAAGCUGCAAG	mmu-miR-5114
288	mmu-miR-5118	3.000	8	mmu-miR-5118	chr16	55494770	55494788	+	miRNA	MIMAT0020626	MI0018027	AAGGUUAGGCCAGCCUGGU	mmu-miR-5118
289	mmu-miR-5119	6.409	85	mmu-miR-5119	chr11	98262638	98262656	+	miRNA	MIMAT0020627	MI0018028	CAUCUCAUCCUGGGGCUGG	mmu-miR-5119
290	mmu-miR-5120	2.585	6	mmu-miR-5120	chr4	44607536	44607558	-	miRNA	MIMAT0020628	MI0018029	UUUGGGGCUGUGGUGCCACCA	mmu-miR-5120
291	mmu-miR-5121	6.728	106	mmu-miR-5121	chr7	45126925	45126945	-	miRNA	MIMAT0020629	MI0018030	AGCUUGUGAUGAGACAUCUCC	mmu-miR-5121
292	mmu-miR-466q	1.585	3	mmu-miR-466q	chr3	28419988	28420007	+	miRNA	MIMAT0020631	MI0018032	GUGCACACACACACAUACGU	mmu-miR-466q
293	mmu-miR-3473d	3.807	14	mmu-miR-3473d	chr8	111016498	111016521	-	miRNA	MIMAT0020632	MI0018033	CCACUGAGCCACUUUCCAGCC	mmu-miR-3473d
294	mmu-miR-5124a	2.322	5	mmu-miR-5124a	chr13	40865801	40865820	+	miRNA	MIMAT0020634	MI0018035	GGUCCAGUGACUAAGAGCAU	mmu-miR-5124a
295	mmu-miR-3572-5p	0.000	1	mmu-miR-3572-5p	chr7	3655970	3655991	+	miRNA	MIMAT0022986	MI0018037	UGGGGAACAGGGCAAGGUGGA	mmu-miR-3572-5p
296	mmu-miR-5126	4.585	24	mmu-miR-5126	chr1	84695890	84695911	+	miRNA	MIMAT0020637	MI0018038	GCGGGCGGGGCCGGGGGCGGG	mmu-miR-5126
297	mmu-miR-5127	0.000	1	mmu-miR-5127	chr18	81992020	81992038	-	miRNA	MIMAT0020638	MI0018039	UCUCCCAACCCUUUUCCCA	mmu-miR-5127
298	mmu-miR-5128	5.615	49	mmu-miR-5128	chr2	37688212	37688234	-	miRNA	MIMAT0020639	MI0018040	CAAUUGGGGCUGGCGAGAUGG	mmu-miR-5128
299	mmu-miR-5129-5p	2.585	6	mmu-miR-5129-5p	chr2	45023146	45023167	-	miRNA	MIMAT0020640	MI0018041	AUGUGGGGGCAUUGGUAUUUU	mmu-miR-5129-5p
300	mmu-miR-5130	3.807	14	mmu-miR-5130	chr14	102982608	102982631	-	miRNA	MIMAT0020641	MI0018042	CUGGAGCGCGCGGGCGAGGCA	mmu-miR-5130
301	mmu-miR-1231-5p	0.000	1	mmu-miR-1231-5p	chr1	135454661	135454683	-	miRNA	MIMAT0022357	MI0019183	UCUGGGCAGAGCUGCAGGAGA	mmu-miR-1231-5p
302	mmu-miR-5622-5p	0.000	1	mmu-miR-5622-5p	chr2	152865067	152865088	+	miRNA	MIMAT0022371	MI0019190	UUCACCACACCCAGCUUAAAG	mmu-miR-5622-5p
303	mmu-miR-6236	8.672	408	mmu-miR-6236	chr9	110281353	110281371	+	miRNA	MIMAT0024857	MI0021583	GCCGUCGCCGGCAGUCAGG	mmu-miR-6236
304	mmu-miR-6237	0.000	1	mmu-miR-6237	chr9	9894484	9894506	+	miRNA	MIMAT0024858	MI0021584	UUAAGGAUUGGGUCUUGGAAU	mmu-miR-6237
305	mmu-miR-6238	6.600	97	mmu-miR-6238	chr7	53891796	53891817	-	miRNA	MIMAT0024859	MI0021585	UUAUUAGUCAGUGGAGGAAAU	mmu-miR-6238
306	mmu-miR-6239	9.773	875	mmu-miR-6239	chr14	117953768	117953787	-	miRNA	MIMAT0024860	MI0021586	UAGCGUUGGAUCACUCGGUG	mmu-miR-6239
307	mmu-miR-6240	4.170	18	mmu-miR-6240	chr5	114851973	114851998	+	miRNA	MIMAT0024861	MI0021587	CCAAAGCAUCGCGAAGGCCCA	mmu-miR-6240
308	mmu-miR-6241	0.000	1	mmu-miR-6241	chr14	118258659	118258677	+	miRNA	MIMAT0024862	MI0021588	CACGGCGGCUGGAAUUCCC	mmu-miR-6241
309	mmu-miR-133c	0.000	1	mmu-miR-133c	chr2	29475217	29475239	-	miRNA	MIMAT0025078	MI0021863	UUUGGUCCCCUUCAAGGAGUC	mmu-miR-133c
310	mmu-miR-6347	1.585	3	mmu-miR-6347	chr1	167290125	167290146	+	miRNA	MIMAT0025090	MI0021875	CAGUGCGGCUGGCUGGAGAUG	mmu-miR-6347
311	mmu-miR-6349	3.585	12	mmu-miR-6349	chr1	39186908	39186929	-	miRNA	MIMAT0025092	MI0021877	UGGAAUGUGGGAGGGGCAUGC	mmu-miR-6349
312	mmu-miR-6359	1.000	2	mmu-miR-6359	chr18	88972087	88972107	+	miRNA	MIMAT0025102	MI0021887	CGAUGUUGCCCAGGGUCAGAA	mmu-miR-6359
313	mmu-miR-6360	0.000	1	mmu-miR-6360	chr18	23774938	23774959	-	miRNA	MIMAT0025103	MI0021888	UAGUGUUGCUCAGGCAGCAGG	mmu-miR-6360

314	mmu-miR-6366	0.000	1	mmu-miR-6366	chr16	18165093	18165114	-	miRNA	MIMAT0025110	MI0021895	AGCUAAGGGGCCCGGGGAGCCA	mmu-miR-6366
315	mmu-miR-6369	1.585	3	mmu-miR-6369	chr13	58313119	58313141	-	miRNA	MIMAT0025113	MI0021898	UGGCUCAGUUCAGUGUUGGUG	mmu-miR-6369
316	mmu-miR-6375	0.000	1	mmu-miR-6375	chr6	85790203	85790225	-	miRNA	MIMAT0025119	MI0021904	GAAUUGGCUGACUGUAUUCCA	mmu-miR-6375
317	mmu-miR-21b	2.322	5	mmu-miR-21b	chr3	29636768	29636790	+	miRNA	MIMAT0025121	MI0021906	UAGUUUAUCAGACUGAUAUUU	mmu-miR-21b
318	mmu-let-7j	16.785	112951	mmu-let-7j	chr3	140028281	140028304	+	miRNA	MIMAT0025123	MI0021908	UGAGGUAUUAGUUUGUGCUGU	mmu-let-7j
319	mmu-miR-6380	1.000	2	mmu-miR-6380	chr3	137783536	137783559	-	miRNA	MIMAT0025126	MI0021911	UGUAAGUGCUUUUAACUGCUG	mmu-miR-6380
320	mmu-miR-6385	0.000	1	mmu-miR-6385	chr9	58221155	58221175	+	miRNA	MIMAT0025131	MI0021916	GCAAGGAUGACAGAGAGGAAG	mmu-miR-6385
321	mmu-miR-6388	1.000	2	mmu-miR-6388	chr12	115695773	115695793	-	miRNA	MIMAT0025135	MI0021920	UGCAGAUGGCACAGUGAGGCC	mmu-miR-6388
322	mmu-miR-378c	9.055	532	mmu-miR-378c	chr14	46954905	46954925	-	miRNA	MIMAT0025138	MI0021923	ACUGGACUUGGAGUCAGAAGC	mmu-miR-378c
323	mmu-miR-6395	6.492	90	mmu-miR-6395	chr8	34166689	34166710	-	miRNA	MIMAT0025146	MI0021930	CUGGCCCUCUCUGCCCUGUUUA	mmu-miR-6395
324	mmu-miR-21c	5.000	32	mmu-miR-21c	chr8	128278188	128278208	-	miRNA	MIMAT0025148	MI0021932	UAGCUUAUCAGACUGGUACAA	mmu-miR-21c
325	mmu-miR-6405	0.000	1	mmu-miR-6405	chr19	44808501	44808523	+	miRNA	MIMAT0025157	MI0021941	GUGGGAAGCAGUGGGAAGCAG	mmu-miR-6405
326	mmu-miR-6406	4.585	24	mmu-miR-6406	chr11	68220917	68220938	+	miRNA	MIMAT0025159	MI0021943	CGCGACCUCAGGCUGACUCAU	mmu-miR-6406
327	mmu-miR-6412	7.570	190	mmu-miR-6412	chr10	122648412	122648433	+	miRNA	MIMAT0025165	MI0021949	UCGAAACCAUCCUCAGCUACUA	mmu-miR-6412
328	mmu-miR-378d	7.229	150	mmu-miR-378d	chr10	126710355	126710376	-	miRNA	MIMAT0025167	MI0021951	ACUGGCCUUGGAGUCAGAAGG	mmu-miR-378d
329	mmu-miR-30f	5.644	50	mmu-miR-30f	chr4	120772621	120772642	+	miRNA	MIMAT0025179	MI0021961	GUAAACAUCCGACUGAAAGCU	mmu-miR-30f
330	mmu-miR-6516-5p	0.000	1	mmu-miR-6516-5p	chr11	117077370	117077391	+	miRNA	MIMAT0027343	MI0022266	UUUGCAGUAACAGGUGUGGAC	mmu-miR-6516-5p
331	mmu-let-7k	6.459	88	mmu-let-7k	chr5	147283257	147283275	-	miRNA	MIMAT0025580	MI0022352	UGAGGUAGGAGGUUGUGUG	mmu-let-7k
332	mmu-miR-6538	4.807	28	mmu-miR-6538	chr12	26414970	26414988	-	miRNA	MIMAT0025583	MI0022354	CGCGGGCUCCGGGGCGGCG	mmu-miR-6538
333	mmu-miR-6540-5p	0.000	1	mmu-miR-6540-5p	chr16	42303425	42303446	-	miRNA	MIMAT0025585	MI0022356	CUAAGGCAGGCAGACUUCAGU	mmu-miR-6540-5p
334	mmu-miR-3473e	9.814	900	mmu-miR-3473e	chr5	31667296	31667316	-	miRNA	MIMAT0025587	MI0022357	GGGCUGGAGAGAUGGCUCGUA	mmu-miR-3473e
335	mmu-miR-6896-5p	1.585	3	mmu-miR-6896-5p	chr1	34117364	34117385	+	miRNA	MIMAT0027692	MI0022743	UUUGUAGGCGACGAGAACCCG	mmu-miR-6896-5p
336	mmu-miR-6900-5p	0.000	1	mmu-miR-6900-5p	chr1	92464514	92464537	-	miRNA	MIMAT0027700	MI0022747	UGCCAGGAGAAGCCUAGAGCC	mmu-miR-6900-5p
337	mmu-miR-6901-5p	0.000	1	mmu-miR-6901-5p	chr1	93274554	93274573	+	miRNA	MIMAT0027702	MI0022748	UGCAGGAAUGCAGAGGACCU	mmu-miR-6901-5p
338	mmu-miR-6906-5p	1.000	2	mmu-miR-6906-5p	chr10	60126777	60126800	+	miRNA	MIMAT0027712	MI0022753	AUGGAUGGGCAGACGGACAGA	mmu-miR-6906-5p
339	mmu-miR-6910-5p	3.700	13	mmu-miR-6910-5p	chr10	79862592	79862614	+	miRNA	MIMAT0027720	MI0022757	UGGGGGUAGGGCACCAGUGGG	mmu-miR-6910-5p
340	mmu-miR-6914-5p	0.000	1	mmu-miR-6914-5p	chr10	128382843	128382866	+	miRNA	MIMAT0027728	MI0022761	UCCUGGGGUGGUGGUGGCCAC	mmu-miR-6914-5p
341	mmu-miR-6915-5p	2.000	4	mmu-miR-6915-5p	chr10	128388837	128388860	+	miRNA	MIMAT0027730	MI0022762	AGGUCAGAGCAGAUGGAGGUU	mmu-miR-6915-5p
342	mmu-miR-6919-5p	1.585	3	mmu-miR-6919-5p	chr11	50232250	50232272	+	miRNA	MIMAT0027738	MI0022766	UAGGCCACUGGAGGUGGAAUU	mmu-miR-6919-5p

343	mmu-miR-6922-5p	0.000	1	mmu-miR-6922-5p	chr11	60202300	60202322	-	miRNA	MIMAT0027744	MI0022769	UGUGGGAGGGGACUGUAGAGA	mmu-miR-6922-5p
344	mmu-miR-6923-5p	0.000	1	mmu-miR-6923-5p	chr11	67099804	67099827	+	miRNA	MIMAT0027746	MI0022770	GUGAGGGCAGGAGGAUUGGGG	mmu-miR-6923-5p
345	mmu-miR-6926-5p	0.000	1	mmu-miR-6926-5p	chr11	74868168	74868189	-	miRNA	MIMAT0027752	MI0022773	UCAGUGGGGUGAGGGAUGGUG	mmu-miR-6926-5p
346	mmu-miR-6927-5p	1.000	2	mmu-miR-6927-5p	chr11	97677060	97677081	+	miRNA	MIMAT0027754	MI0022774	GUGAGGGGAUCCAGCCCAGGC	mmu-miR-6927-5p
347	mmu-miR-6934-5p	2.585	6	mmu-miR-6934-5p	chr11	119154111	119154132	-	miRNA	MIMAT0027768	MI0022781	UUGGUGGAGACAGCAAGGGUG	mmu-miR-6934-5p
348	mmu-miR-6937-5p	3.459	11	mmu-miR-6937-5p	chr12	28679325	28679348	+	miRNA	MIMAT0027774	MI0022784	UAGCUGUAAGGGCUGGGUCUG	mmu-miR-6937-5p
349	mmu-miR-6942-5p	2.322	5	mmu-miR-6942-5p	chr13	21396613	21396633	-	miRNA	MIMAT0027784	MI0022789	UAGGAGGAGGGGAACAUUUGC	mmu-miR-6942-5p
350	mmu-miR-6944-5p	0.000	1	mmu-miR-6944-5p	chr13	55477754	55477776	-	miRNA	MIMAT0027788	MI0022791	GUGAGAGCGGGGGGAGUGGCA	mmu-miR-6944-5p
351	mmu-miR-6953-5p	2.322	5	mmu-miR-6953-5p	chr15	76248196	76248221	+	miRNA	MIMAT0027806	MI0022800	AAGGGGCAGGGGCAGGGAUUC	mmu-miR-6953-5p
352	mmu-miR-6954-5p	1.000	2	mmu-miR-6954-5p	chr15	76433219	76433240	+	miRNA	MIMAT0027808	MI0022801	UGGGGCAGUUCUGGGGGCAGA	mmu-miR-6954-5p
353	mmu-miR-6958-5p	1.000	2	mmu-miR-6958-5p	chr15	89185516	89185537	-	miRNA	MIMAT0027816	MI0022805	GUGGGAGAAGAGGCUGCUGUG	mmu-miR-6958-5p
354	mmu-miR-6959-5p	0.000	1	mmu-miR-6959-5p	chr15	89305705	89305727	-	miRNA	MIMAT0027818	MI0022806	UGGGAAACCUGUGUCGGGCUG	mmu-miR-6959-5p
355	mmu-miR-6963-5p	2.000	4	mmu-miR-6963-5p	chr15	103350497	103350520	-	miRNA	MIMAT0027826	MI0022810	GGUGGGAUGGAUGGCAGAACC	mmu-miR-6963-5p
356	mmu-miR-3547-5p	0.000	1	mmu-miR-3547-5p	chr17	25245614	25245638	-	miRNA	MIMAT0027832	MI0022813	GUGGGAAGAGGGGUGGGGCCC	mmu-miR-3547-5p
357	mmu-miR-6970-5p	4.644	25	mmu-miR-6970-5p	chr17	34845143	34845167	-	miRNA	MIMAT0027842	MI0022818	GUAAGUUCAGGGCUGGGAGCA	mmu-miR-6970-5p
358	mmu-miR-6973a-5p	1.585	3	mmu-miR-6973a-5p	chr17	35194977	35194998	+	miRNA	MIMAT0027848	MI0022821	UACGGUGGGAGGGGUGGAGUU	mmu-miR-6973a-5p
359	mmu-miR-6974-5p	2.322	5	mmu-miR-6974-5p	chr17	35204534	35204557	-	miRNA	MIMAT0027850	MI0022822	GUGAGGCAGCAAGAGAUUGGG	mmu-miR-6974-5p
360	mmu-miR-6976-5p	2.585	6	mmu-miR-6976-5p	chr17	46553862	46553884	-	miRNA	MIMAT0027854	MI0022824	CAGGGAAGUUGAGAGGAAAAU	mmu-miR-6976-5p
361	mmu-miR-6977-5p	1.000	2	mmu-miR-6977-5p	chr17	56418885	56418909	-	miRNA	MIMAT0027856	MI0022825	GUGAGGCGCUGUGGGCACGGC	mmu-miR-6977-5p
362	mmu-miR-6978-5p	1.000	2	mmu-miR-6978-5p	chr17	57217201	57217223	-	miRNA	MIMAT0027858	MI0022826	AAGGGGUGAGAGAGAAGCUGG	mmu-miR-6978-5p
363	mmu-miR-6981-5p	1.000	2	mmu-miR-6981-5p	chr18	37974592	37974617	-	miRNA	MIMAT0027864	MI0022829	GUGAGGAGAAGGAAGAGGCUG	mmu-miR-6981-5p
364	mmu-miR-6985-5p	4.087	17	mmu-miR-6985-5p	chr19	4263852	4263873	-	miRNA	MIMAT0027872	MI0022833	UACUGAGGGGUGGCUGCUAUG	mmu-miR-6985-5p
365	mmu-miR-6987-5p	1.000	2	mmu-miR-6987-5p	chr19	5679050	5679071	-	miRNA	MIMAT0027876	MI0022835	UGGGACAGGGUGACAGGGUGA	mmu-miR-6987-5p
366	mmu-miR-6990-5p	0.000	1	mmu-miR-6990-5p	chr19	6914261	6914281	-	miRNA	MIMAT0027882	MI0022838	CCCAGGGUGAGUCAGGGCUCU	mmu-miR-6990-5p
367	mmu-miR-6995-5p	1.585	3	mmu-miR-6995-5p	chr19	47273562	47273584	-	miRNA	MIMAT0027892	MI0022843	CUGGGAGUAGAAGGGGGAAAC	mmu-miR-6995-5p
368	mmu-miR-6996-5p	0.000	1	mmu-miR-6996-5p	chr2	26470094	26470114	-	miRNA	MIMAT0027894	MI0022844	UGCACAGGACAGAGCACAGUC	mmu-miR-6996-5p
369	mmu-miR-6998-5p	0.000	1	mmu-miR-6998-5p	chr2	31612426	31612447	+	miRNA	MIMAT0027898	MI0022846	CUGGGCAGAGGGCAAAGUGAC	mmu-miR-6998-5p
370	mmu-miR-7003-5p	1.000	2	mmu-miR-7003-5p	chr2	163068095	163068115	+	miRNA	MIMAT0027910	MI0022852	UGUGGGGAGAAGCUGCGGCAG	mmu-miR-7003-5p
371	mmu-miR-7007-5p	2.322	5	mmu-miR-7007-5p	chr3	20222332	20222354	-	miRNA	MIMAT0027918	MI0022856	UCAGAAGAGGCAGUGGAGGAG	mmu-miR-7007-5p

372	mmu-miR-7010-5p	2.585	6	mmu-miR-7010-5p	chr3	82106284	82106305	-	miRNA	MIMAT0027924	MI0022859	CGGAAAGGAUUGGAAACUUG	mmu-miR-7010-5p
373	mmu-miR-7012-5p	3.170	9	mmu-miR-7012-5p	chr3	90270154	90270176	+	miRNA	MIMAT0027928	MI0022861	AAGGAGAGGAGUUGGCAGGGA	mmu-miR-7012-5p
374	mmu-miR-7014-5p	0.000	1	mmu-miR-7014-5p	chr3	95734947	95734968	-	miRNA	MIMAT0027932	MI0022863	UUGGGUGCUGUGGAAGGGACA	mmu-miR-7014-5p
375	mmu-miR-7015-5p	0.000	1	mmu-miR-7015-5p	chr4	120973295	120973317	-	miRNA	MIMAT0027934	MI0022864	UCUGUGCAGUAGGCUGUGGGU	mmu-miR-7015-5p
376	mmu-miR-7016-5p	0.000	1	mmu-miR-7016-5p	chr4	129684546	129684566	-	miRNA	MIMAT0027936	MI0022865	CAGGGAGGGGAGCGAGAGUAG	mmu-miR-7016-5p
377	mmu-miR-7024-5p	0.000	1	mmu-miR-7024-5p	chr5	33898946	33898966	-	miRNA	MIMAT0027952	MI0022873	UUGGGGGAUGGGUUGCUUGGC	mmu-miR-7024-5p
378	mmu-miR-7028-5p	1.585	3	mmu-miR-7028-5p	chr5	114719515	114719537	+	miRNA	MIMAT0027960	MI0022877	UGGGCUGAGGCUUGGGUCAGG	mmu-miR-7028-5p
379	mmu-miR-7031-5p	3.000	8	mmu-miR-7031-5p	chr5	121819246	121819268	-	miRNA	MIMAT0027966	MI0022880	CCUGAGAGGCCUGAAGGGUGG	mmu-miR-7031-5p
380	mmu-miR-7033-5p	4.700	26	mmu-miR-7033-5p	chr5	135383783	135383805	-	miRNA	MIMAT0027970	MI0022882	UCUCCAGGAGUCUGAGGGGCA	mmu-miR-7033-5p
381	mmu-miR-7034-5p	0.000	1	mmu-miR-7034-5p	chr5	135735864	135735884	-	miRNA	MIMAT0027972	MI0022883	UCCGGGAGGGAUGGAUGUGCU	mmu-miR-7034-5p
382	mmu-miR-7036a-5p	0.000	1	mmu-miR-7036a-5p	chr5	137296661	137296683	-	miRNA	MIMAT0027976	MI0022885	AGCGGGGUUCGGUGGGGAAGA	mmu-miR-7036a-5p
383	mmu-miR-7038-5p	1.000	2	mmu-miR-7038-5p	chr5	140427344	140427365	+	miRNA	MIMAT0027980	MI0022887	UGUAGAAGGAAGGGCUGCUGU	mmu-miR-7038-5p
384	mmu-miR-7043-5p	1.585	3	mmu-miR-7043-5p	chr6	116646020	116646042	-	miRNA	MIMAT0027990	MI0022892	UGUGAAAGCAGAGAGGCAUUU	mmu-miR-7043-5p
385	mmu-miR-7045-5p	0.000	1	mmu-miR-7045-5p	chr6	125097029	125097049	+	miRNA	MIMAT0027994	MI0022894	AGCGGGUGGGGGAGGGGGACU	mmu-miR-7045-5p
386	mmu-miR-7046-5p	1.000	2	mmu-miR-7046-5p	chr7	24970047	24970070	-	miRNA	MIMAT0027996	MI0022895	UGUAGGGUGAGGCUGGGAGCC	mmu-miR-7046-5p
387	mmu-miR-7048-5p	1.585	3	mmu-miR-7048-5p	chr7	25217699	25217720	-	miRNA	MIMAT0028000	MI0022897	CGGGGCUGAGAGGUGAGGAAG	mmu-miR-7048-5p
388	mmu-miR-7050-5p	0.000	1	mmu-miR-7050-5p	chr7	31040292	31040312	-	miRNA	MIMAT0028004	MI0022899	ACAGGAGAAGGGGGUGAGAGA	mmu-miR-7050-5p
389	mmu-miR-7053-5p	0.000	1	mmu-miR-7053-5p	chr7	44538153	44538173	-	miRNA	MIMAT0028010	MI0022902	UGGGGAAAGGCAGGCUACUGG	mmu-miR-7053-5p
390	mmu-miR-7060-5p	0.000	1	mmu-miR-7060-5p	chr7	127489251	127489272	+	miRNA	MIMAT0028024	MI0022909	GUGAGUGCUGGGUAGAAUGGG	mmu-miR-7060-5p
391	mmu-miR-7063-5p	1.585	3	mmu-miR-7063-5p	chr7	141620706	141620728	+	miRNA	MIMAT0028030	MI0022912	AUGAGAGGUGCAGGCUGAGCA	mmu-miR-7063-5p
392	mmu-miR-7068-5p	0.000	1	mmu-miR-7068-5p	chr8	72470069	72470089	-	miRNA	MIMAT0028042	MI0022918	GUGAGGCUCAGUAUGGGGUGG	mmu-miR-7068-5p
393	mmu-miR-7070-5p	2.322	5	mmu-miR-7070-5p	chr8	85062086	85062106	+	miRNA	MIMAT0028046	MI0022920	GUGAGGGGAGCUGAGGCAGGA	mmu-miR-7070-5p
394	mmu-miR-7071-5p	3.000	8	mmu-miR-7071-5p	chr8	88138211	88138233	+	miRNA	MIMAT0028048	MI0022921	CCCGGGAGCGAGGUGCUGCAG	mmu-miR-7071-5p
395	mmu-miR-7078-5p	2.000	4	mmu-miR-7078-5p	chr8	117459270	117459289	+	miRNA	MIMAT0028062	MI0022928	UGUGGGUGGUAGGAGACGCU	mmu-miR-7078-5p
396	mmu-miR-7079-5p	0.000	1	mmu-miR-7079-5p	chr8	123104980	123105001	+	miRNA	MIMAT0028064	MI0022929	AGGGCUGAGGCAGUGAGUCCU	mmu-miR-7079-5p
397	mmu-miR-7080-5p	0.000	1	mmu-miR-7080-5p	chr8	123130106	123130128	+	miRNA	MIMAT0028066	MI0022930	GUAGGAGCUGGAGGUGGGUUU	mmu-miR-7080-5p
398	mmu-miR-7081-5p	2.000	4	mmu-miR-7081-5p	chr9	20914134	20914159	-	miRNA	MIMAT0028068	MI0022931	AGAGGAGGGUGCUCGGCCGGG	mmu-miR-7081-5p
399	mmu-miR-7082-5p	1.000	2	mmu-miR-7082-5p	chr9	21075563	21075583	-	miRNA	MIMAT0028070	MI0022932	UACGGGCAGGAGGAGGGGAGG	mmu-miR-7082-5p
400	mmu-miR-7084-5p	1.000	2	mmu-miR-7084-5p	chr9	22113988	22114008	-	miRNA	MIMAT0028074	MI0022934	UAGAGGAUAGAGGUAGAGAGU	mmu-miR-7084-5p

401	mmu-miR-7086-5p	2.585	6	mmu-miR-7086-5p	chr9	45266682	45266702	-	miRNA	MIMAT0028078	MI0022936	AAGAGGAGAAAGGUUUGGGCA	mmu-miR-7086-5p
402	mmu-miR-7087-5p	0.000	1	mmu-miR-7087-5p	chr9	45939565	45939585	-	miRNA	MIMAT0028080	MI0022937	AGGCAGGUGUGGAGCUGGUCU	mmu-miR-7087-5p
403	mmu-miR-7089-5p	1.000	2	mmu-miR-7089-5p	chr9	109889648	109889671	+	miRNA	MIMAT0028084	MI0022939	CCGUGGGACCUACAGACUCCU	mmu-miR-7089-5p
404	mmu-miR-7093-5p	2.585	6	mmu-miR-7093-5p	chrX	134757636	134757657	+	miRNA	MIMAT0028092	MI0022943	CAGGAUGACAGAAGGAAAACC	mmu-miR-7093-5p
405	mmu-miR-7115-5p	1.585	3	mmu-miR-7115-5p	chr11	70438256	70438279	+	miRNA	MIMAT0028127	MI0022966	UCGGGGGUUGUGGUGCCGAAU	mmu-miR-7115-5p
406	mmu-miR-7116-5p	1.585	3	mmu-miR-7116-5p	chr11	100478455	100478475	-	miRNA	MIMAT0028129	MI0022967	UGAAGACAUCAGGAAAAAAAA	mmu-miR-7116-5p
407	mmu-miR-7118-5p	1.585	3	mmu-miR-7118-5p	chr15	89162882	89162903	-	miRNA	MIMAT0028133	MI0022969	UGGGGAAGGCGGAGAGGGAA	mmu-miR-7118-5p
408	mmu-miR-7210-5p	2.807	7	mmu-miR-7210-5p	chr14	24088877	24088899	+	miRNA	MIMAT0028388	MI0023705	UAACAUUGUAGACAGGCACAA	mmu-miR-7210-5p
409	mmu-miR-7212-5p	0.000	1	mmu-miR-7212-5p	chr15	25948227	25948247	+	miRNA	MIMAT0028392	MI0023707	UCUGGGGGCUUGUGUGGUAGG	mmu-miR-7212-5p
410	mmu-miR-7214-5p	1.000	2	mmu-miR-7214-5p	chr17	27317073	27317095	-	miRNA	MIMAT0028396	MI0023709	UGUUUUCUGGGUUGGAAUGAG	mmu-miR-7214-5p
411	mmu-miR-7216-5p	2.000	4	mmu-miR-7216-5p	chr17	27328385	27328408	+	miRNA	MIMAT0028400	MI0023711	UGGAGAGCUGGCAGAGGACCC	mmu-miR-7216-5p
412	mmu-miR-7229-5p	0.000	1	mmu-miR-7229-5p	chr5	113324525	113324545	-	miRNA	MIMAT0028426	MI0023724	UAGUAGACAUCCCUGGAUAGC	mmu-miR-7229-5p
413	mmu-miR-7230-5p	0.000	1	mmu-miR-7230-5p	chr5	113337623	113337645	-	miRNA	MIMAT0028428	MI0023725	AGACUGCUGUUUCUCUGAGUG	mmu-miR-7230-5p
414	mmu-miR-7233-5p	0.000	1	mmu-miR-7233-5p	chr6	127788125	127788143	-	miRNA	MIMAT0028434	MI0023728	AGUUAGGGACAGAUAGAUG	mmu-miR-7233-5p
415	mmu-miR-219b-5p	0.000	1	mmu-miR-219b-5p	chr2	29845647	29845668	+	miRNA	MIMAT0029806	MI0024990	AGAUGUCCAGCCACAAUUCUC	mmu-miR-219b-5p
416	mmu-miR-7652-5p	2.585	6	mmu-miR-7652-5p	chr11	55501226	55501247	+	miRNA	MIMAT0029810	MI0024992	UAAGGGCACAGGGAUUUCAGG	mmu-miR-7652-5p
417	mmu-miR-7658-5p	0.000	1	mmu-miR-7658-5p	chr4	156230096	156230115	-	miRNA	MIMAT0029822	MI0024998	UGUGGGCGUGGCGGUGCUGG	mmu-miR-7658-5p
418	mmu-miR-7662-5p	0.000	1	mmu-miR-7662-5p	chr10	62194206	62194227	-	miRNA	MIMAT0029830	MI0025002	AGGGCUGAGGCCUGGAUCCAG	mmu-miR-7662-5p
419	mmu-miR-7667-5p	5.129	35	mmu-miR-7667-5p	chr17	29595538	29595559	+	miRNA	MIMAT0029840	MI0025007	GAGCCAUCUCUCUAGCCCCUG	mmu-miR-7667-5p
420	mmu-miR-7669-5p	0.000	1	mmu-miR-7669-5p	chr3	90011188	90011210	-	miRNA	MIMAT0029844	MI0025009	AGUACCACCAUACACAGCUUU	mmu-miR-7669-5p
421	mmu-miR-129b-5p	1.585	3	mmu-miR-129b-5p	chr2	94241378	94241399	+	miRNA	MIMAT0029862	MI0025019	GCUUUUUGGGGUAAGGGCUUC	mmu-miR-129b-5p
422	mmu-miR-7677-5p	1.585	3	mmu-miR-7677-5p	chr17	27091245	27091269	+	miRNA	MIMAT0029868	MI0025022	AGUGGUGAGCAGAAAGCAGCC	mmu-miR-7677-5p
423	mmu-miR-7679-5p	1.000	2	mmu-miR-7679-5p	chr11	82985046	82985065	-	miRNA	MIMAT0029872	MI0025024	AGGCUGGCACCAGGAUCCCU	mmu-miR-7679-5p
424	mmu-miR-3620-5p	3.322	10	mmu-miR-3620-5p	chrX	150547424	150547445	-	miRNA	MIMAT0029878	MI0025027	CUGUGGGCUGGGCUGGGAAGC	mmu-miR-3620-5p
425	mmu-miR-7683-5p	1.000	2	mmu-miR-7683-5p	chr1	171641879	171641900	-	miRNA	MIMAT0029886	MI0025031	UUCCGUGUUUCGUCUGACCAC	mmu-miR-7683-5p
426	mmu-miR-7684-5p	0.000	1	mmu-miR-7684-5p	chr15	82393958	82393978	-	miRNA	MIMAT0029890	MI0025033	UCUGGGAAGCCUGGGCAGCAG	mmu-miR-7684-5p
427	mmu-miR-219c-5p	1.585	3	mmu-miR-219c-5p	chr17	34025012	34025033	+	miRNA	MIMAT0029892	MI0025034	GGACGUCCAGACGCAACUCUC	mmu-miR-219c-5p
428	mmu-miR-126b-5p	6.644	100	mmu-miR-126b-5p	chr2	26591399	26591420	-	miRNA	MIMAT0029894	MI0025035	AUUAUUACUCACGGUACGAGU	mmu-miR-126b-5p
429	mmu-miR-7685-5p	4.087	17	mmu-miR-7685-5p	chr2	158243542	158243565	-	miRNA	MIMAT0029896	MI0025036	ACCUUCCGGUUUCUUCAAGUC	mmu-miR-7685-5p

430	mmu-miR-7687-5p	4.459	22	mmu-miR-7687-5p	chr8	120538696	120538720	+	miRNA	MIMAT0029902	MI0025039	AGGCGGGGAACCUGAGGCGCA	mmu-miR-7687-5p
431	mmu-miR-1258-5p	3.700	13	mmu-miR-1258-5p	chr18	56538139	56538160	+	miRNA	MIMAT0029904	MI0025040	UGCUGAGCUAAUUCCCUAACU	mmu-miR-1258-5p
432	mmu-miR-7688-5p	6.358	82	mmu-miR-7688-5p	chr10	93433107	93433129	+	miRNA	MIMAT0029906	MI0025041	UAGCUGGGCAUGAUCUGAUGA	mmu-miR-7688-5p
433	mmu-miR-3473f	2.000	4	mmu-miR-3473f	chr1	106546536	106546555	-	miRNA	MIMAT0031390	MI0026016	CAAAUAGGACUGGAGAGAUG	mmu-miR-3473f
434	mmu-miR-8095	1.000	2	mmu-miR-8095	chr16	22532128	22532149	-	miRNA	MIMAT0031396	MI0026022	AAAGGAUUCUGCUGUCUGUCC	mmu-miR-8095
435	mmu-miR-1291	3.807	14	mmu-miR-1291	chr15	98519828	98519853	-	miRNA	MIMAT0031397	MI0026023	AUGGCUCUUACUGAAGACUAG	mmu-miR-1291
436	mmu-miR-142b	0.000	1	mmu-miR-142b	chr11	87756906	87756925	-	miRNA	MIMAT0031402	MI0026028	UCCAUAAAGUAGGAAACACU	mmu-miR-142b
437	mmu-miR-8100	0.000	1	mmu-miR-8100	chr11	46102300	46102322	+	miRNA	MIMAT0031403	MI0026029	AGGAGGAAAGGGAGCAAGCAG	mmu-miR-8100
438	mmu-miR-497b	0.000	1	mmu-miR-497b	chr11	70234763	70234786	-	miRNA	MIMAT0031404	MI0026030	CACCACAGUGUGGUUUGGACG	mmu-miR-497b
439	mmu-miR-8102	2.000	4	mmu-miR-8102	chr11	97744974	97744995	+	miRNA	MIMAT0031406	MI0026032	UCACGCGGGGGAACGAGGAAG	mmu-miR-8102
440	mmu-miR-8103	2.322	5	mmu-miR-8103	chr11	97063829	97063849	+	miRNA	MIMAT0031407	MI0026033	UCUCCUGUUCUCUGUUCUCCC	mmu-miR-8103
441	mmu-miR-3535	7.267	154	mmu-miR-3535	chr1	86352062	86352087	-	miRNA	MIMAT0031410	MI0026036	UGGAUAUGAUGACUGAUUACC	mmu-miR-3535
442	mmu-miR-8109	1.000	2	mmu-miR-8109	chr8	85700935	85700955	+	miRNA	MIMAT0031415	MI0026041	GCGCCGCGUGCCGGCCGCGGG	mmu-miR-8109
443	mmu-miR-8110	1.585	3	mmu-miR-8110	chr8	89024791	89024811	+	miRNA	MIMAT0031416	MI0026042	AAGCGUGGAUUGGGGGGGGG	mmu-miR-8110
444	mmu-miR-8112	0.000	1	mmu-miR-8112	chr6	71271745	71271764	+	miRNA	MIMAT0031418	MI0026044	UCUCCGCCACCUCCACCGCA	mmu-miR-8112
445	mmu-miR-8113	5.907	60	mmu-miR-8113	chr6	125234756	125234777	-	miRNA	MIMAT0031419	MI0026045	CAGGAGAGUCAGGGGCAAGUA	mmu-miR-8113
446	mmu-miR-8117	2.000	4	mmu-miR-8117	chr5	50252823	50252843	+	miRNA	MIMAT0031423	MI0026049	GCUCGUGUGGAACAGAAGGGG	mmu-miR-8117
447	mmu-miR-3473g	4.000	16	mmu-miR-3473g	chr2	126902290	126902309	-	miRNA	MIMAT0031427	MI0026054	CAAAAGUGAGGCUGGGGAGA	mmu-miR-3473g
448	mmu-miR-3154	3.585	12	mmu-miR-3154	chr2	32318317	32318338	+	miRNA	MIMAT0035714	MI0030343	CAGAAGGGGAGUCGGGAGCGG	mmu-miR-3154
449	mmu-miR-1271-5p	0.000	1	mmu-miR-1271-5p	chr13	54578152	54578173	+	miRNA	MIMAT0048641	MI0039503	CUUGGCACCUGGUAAGCACUC	mmu-miR-1271-5p
450	mmu-miR-12179-5p	0.000	1	mmu-miR-12179-5p	chr1	177579511	177579529	-	miRNA	MIMAT0049824	MI0040617	UCUCUGUCCUCCAGUUCUG	mmu-miR-12179-5p
451	mmu-miR-203b-5p	8.313	318	mmu-miR-203b-5p	chr12	112130927	112130946	-	miRNA	MIMAT0049831	MI0040622	AGUGGUCCUAAACAUUUCAC	mmu-miR-203b-5p
452	mmu-miR-9b-5p	2.585	6	mmu-miR-9b-5p	chr13	83738856	83738876	-	miRNA	MIMAT0049835	MI0040624	UUCGGUUAUCUAGCUUUAUGA	mmu-miR-9b-5p
453	mmu-miR-12190-3p	0.000	1	mmu-miR-12190-3p	chr17	87220142	87220159	-	miRNA	MIMAT0049849	MI0040632	UGGAGAACUCUGGGGGAG	mmu-miR-12190-3p
454	mmu-miR-12194-3p	0.000	1	mmu-miR-12194-3p	chr3	55887284	55887305	-	miRNA	MIMAT0049858	MI0040638	UCUGUGGGUCUGUUUGUCCGU	mmu-miR-12194-3p
455	mmu-miR-3473h-5p	1.585	3	mmu-miR-3473h-5p	chr3	116667598	116667617	-	miRNA	MIMAT0049861	MI0040641	UAGGGGCUGGAAAGGUGACU	mmu-miR-3473h-5p
456	mmu-miR-12200-5p	0.000	1	mmu-miR-12200-5p	chr5	125781683	125781704	+	miRNA	MIMAT0049867	MI0040646	AAACAAACCAGAGGCUCACACU	mmu-miR-12200-5p
457	mmu-miR-12202-5p	3.170	9	mmu-miR-12202-5p	chr7	4917965	4917988	+	miRNA	MIMAT0049871	MI0040648	UGGUGCCUGGAUUGGAGGAUG	mmu-miR-12202-5p
458	mmu-miR-1970c-5p	4.322	20	mmu-miR-1970c-5p	chr9	74966743	74966765	+	miRNA	MIMAT0049876	MI0040652	UGUGUCACUGGGGUUAUGCUU	mmu-miR-1970c-5p

459	mmu-let-7g-3p	1.585	3	mmu-let-7g-3p	chr9	106178902	106178923	+	miRNA	MIMAT0004519	MI0000137	ACUGUACAGGCCACUGCCUUG	mmu-let-7g-3p
460	mmu-let-7i-3p	2.000	4	mmu-let-7i-3p	chr10	122985642	122985663	-	miRNA	MIMAT0004520	MI0000138	CUGCGCAAGCUACUGCCUUGC	mmu-let-7i-3p
461	mmu-miR-1a-3p	5.000	32	mmu-miR-1a-3p	chr18	10785485	10785506	-	miRNA	MIMAT0000123	MI0000652	UGGAAUGUAAAGAAGUAUGUA	mmu-miR-1a-3p
462	mmu-miR-15b-3p	7.170	144	mmu-miR-15b-3p	chr3	69009813	69009834	+	miRNA	MIMAT0004521	MI0000140	CGAAUCAUUAUUUGCUGCUCU	mmu-miR-15b-3p
463	mmu-miR-23b-3p	8.224	299	mmu-miR-23b-3p	chr13	63300529	63300549	+	miRNA	MIMAT0000125	MI0000141	AUCACAUUGCCAGGGAUUACC	mmu-miR-23b-3p
464	mmu-miR-27b-3p	10.444	1393	mmu-miR-27b-3p	chr13	63300760	63300780	+	miRNA	MIMAT0000126	MI0000142	UUCACAGUGGCUAAGUUCUGC	mmu-miR-27b-3p
465	mmu-miR-29b-3p	5.044	33	mmu-miR-29b-3p	chr1	195037091	195037113	+	miRNA	MIMAT0000127	MI0000712	UAGCACCAUUUGAAAUCAGUG	mmu-miR-29b-3p
466	mmu-miR-30a-3p	11.046	2114	mmu-miR-30a-3p	chr1	23272315	23272336	+	miRNA	MIMAT0000129	MI0000144	CUUUCAGUCGGAUGUUUGCAG	mmu-miR-30a-3p
467	mmu-miR-30b-3p	6.585	96	mmu-miR-30b-3p	chr15	68337431	68337452	-	miRNA	MIMAT0004524	MI0000145	CUGGGAUGUGGAUGUUUACGU	mmu-miR-30b-3p
468	mmu-miR-99b-3p	6.768	109	mmu-miR-99b-3p	chr17	17830232	17830253	+	miRNA	MIMAT0004525	MI0000147	CAAGCUCGUGUCUGUGGGUCC	mmu-miR-99b-3p
469	mmu-miR-101a-3p	5.858	58	mmu-miR-101a-3p	chr4	101346957	101346977	-	miRNA	MIMAT0000133	MI0000148	UACAGUACUGUGAUAACUGAA	mmu-miR-101a-3p
470	mmu-miR-124-3p	3.170	9	mmu-miR-124-3p	chr14	64590709	64590728	+	miRNA	MIMAT0000134	MI0000716	UAAGGCACGCGGUGAAUGCC	mmu-miR-124-3p
471	mmu-miR-125a-3p	6.248	76	mmu-miR-125a-3p	chr17	17830855	17830876	+	miRNA	MIMAT0004528	MI0000151	ACAGGUGAGGUUCUUGGGAGC	mmu-miR-125a-3p
472	mmu-miR-125b-2-3p	5.000	32	mmu-miR-125b-2-3p	chr16	77646318	77646339	+	miRNA	MIMAT0004529	MI0000152	ACAAGUCAGGUUCUUGGGACC	mmu-miR-125b-2-3p
473	mmu-miR-126a-3p	10.594	1546	mmu-miR-126a-3p	chr2	26591402	26591423	+	miRNA	MIMAT0000138	MI0000153	UCGUACCGUGAGUAAUAAUGC	mmu-miR-126a-3p
474	mmu-miR-127-3p	8.916	483	mmu-miR-127-3p	chr12	109592888	109592909	+	miRNA	MIMAT0000139	MI0000154	UCGGAUCCGUCUGAGCUUGGC	mmu-miR-127-3p
475	mmu-miR-128-3p	11.292	2507	mmu-miR-128-3p	chr1	128202404	128202424	+	miRNA	MIMAT0000140	MI0000155	UCACAGUGAACCGGUCUCUUU	mmu-miR-128-3p
476	mmu-miR-130a-3p	5.807	56	mmu-miR-130a-3p	chr2	84741116	84741137	-	miRNA	MIMAT0000141	MI0000156	CAGUGCAAUGUUAAAAGGGCA	mmu-miR-130a-3p
477	mmu-miR-9-3p	6.209	74	mmu-miR-9-3p	chr13	83738858	83738879	+	miRNA	MIMAT0000143	MI0000157	AUAAAGCUAGAUAACCGAAAG	mmu-miR-9-3p
478	mmu-miR-132-3p	3.907	15	mmu-miR-132-3p	chr11	75173723	75173744	+	miRNA	MIMAT0000144	MI0000158	UAACAGUCUACAGCCAUGGUC	mmu-miR-132-3p
479	mmu-miR-136-3p	0.000	1	mmu-miR-136-3p	chr12	109595366	109595387	+	miRNA	MIMAT0004532	MI0000162	AUCAUCGUCUCAAAUGAGUCU	mmu-miR-136-3p
480	mmu-miR-140-3p	6.150	71	mmu-miR-140-3p	chr8	107551288	107551308	+	miRNA	MIMAT0000152	MI0000165	UACCACAGGGUAGAACCACGG	mmu-miR-140-3p
481	mmu-miR-141-3p	0.000	1	mmu-miR-141-3p	chr6	124717917	124717938	-	miRNA	MIMAT0000153	MI0000166	UAACACUGUCUGGUAAAGAUG	mmu-miR-141-3p
482	mmu-miR-142a-3p	5.358	41	mmu-miR-142a-3p	chr11	87756903	87756925	+	miRNA	MIMAT0000155	MI0000167	UGUAGUGUUUCCUACUUUAUG	mmu-miR-142a-3p
483	mmu-miR-144-3p	2.322	5	mmu-miR-144-3p	chr11	78073047	78073066	+	miRNA	MIMAT0000156	MI0000168	UACAGUAUAGAUGAUGUACU	mmu-miR-144-3p
484	mmu-miR-145a-3p	6.644	100	mmu-miR-145a-3p	chr18	61647827	61647848	-	miRNA	MIMAT0004534	MI0000169	AUUCCUGGAAAUACUGUUCUU	mmu-miR-145a-3p
485	mmu-miR-149-3p	5.644	50	mmu-miR-149-3p	chr1	92850421	92850442	+	miRNA	MIMAT0016990	MI0000171	GAGGGAGGGACGGGGGCGGUG	mmu-miR-149-3p
486	mmu-miR-150-3p	1.000	2	mmu-miR-150-3p	chr7	45121798	45121819	+	miRNA	MIMAT0004535	MI0000172	CUGGUACAGGCCUGGGGGAUA	mmu-miR-150-3p
487	mmu-miR-151-3p	10.873	1876	mmu-miR-151-3p	chr15	73254820	73254840	-	miRNA	MIMAT0000161	MI0000173	CUAGACUGAGGCUCCUUGAGG	mmu-miR-151-3p

488	mmu-miR-152-3p	6.459	88	mmu-miR-152-3p	chr11	96850439	96850459	+	miRNA	MIMAT0000162	MI0000174	UCAGUGCAUGACAGAACUUGG	mmu-miR-152-3p
489	mmu-miR-10b-3p	5.700	52	mmu-miR-10b-3p	chr2	74726114	74726135	+	miRNA	MIMAT0004538	MI0000221	CAGAUUCGAUUCUAGGGGAAU	mmu-miR-10b-3p
490	mmu-miR-129-1-3p	3.700	13	mmu-miR-129-1-3p	chr6	29022667	29022688	+	miRNA	MIMAT0016994	MI0000222	AAGCCCUUACCCCAAAAAGUA	mmu-miR-129-1-3p
491	mmu-miR-181a-2-3p	1.585	3	mmu-miR-181a-2-3p	chr2	38852785	38852806	+	miRNA	MIMAT0005443	MI0000223	ACCACCGACCGUUGACUGUAC	mmu-miR-181a-2-3p
492	mmu-miR-184-3p	9.386	669	mmu-miR-184-3p	chr9	89802263	89802284	-	miRNA	MIMAT0000213	MI0000226	UGGACGGAGAACUGAUAAGGG	mmu-miR-184-3p
493	mmu-miR-185-3p	2.322	5	mmu-miR-185-3p	chr16	18327405	18327425	-	miRNA	MIMAT0016996	MI0000227	AGGGGCUGGCUUUCCUCUGGU	mmu-miR-185-3p
494	mmu-miR-186-3p	2.000	4	mmu-miR-186-3p	chr3	157544324	157544345	+	miRNA	MIMAT0004540	MI0000228	GCCCUAAGGUGAAUUUUUUGG	mmu-miR-186-3p
495	mmu-miR-187-3p	5.170	36	mmu-miR-187-3p	chr18	24429110	24429131	-	miRNA	MIMAT0000216	MI0000229	UCGUGUCUUGUGUUGCAGCCG	mmu-miR-187-3p
496	mmu-miR-24-3p	12.161	4581	mmu-miR-24-3p	chr13	63301251	63301272	+	miRNA	MIMAT0000219	MI0000231	UGGCUCAGUUCAGCAGGAACA	mmu-miR-24-3p
497	mmu-miR-191-3p	4.170	18	mmu-miR-191-3p	chr9	108568367	108568388	+	miRNA	MIMAT0004542	MI0000233	GCUGCACUUGGAUUUCGUUCC	mmu-miR-191-3p
498	mmu-miR-195a-3p	3.000	8	mmu-miR-195a-3p	chr11	70235100	70235121	+	miRNA	MIMAT0017000	MI0000237	CCAAUAUUGGCUGUGCUGCUC	mmu-miR-195a-3p
499	mmu-miR-199a-3p	9.229	600	mmu-miR-199a-3p	chr1	162217883	162217904	+	miRNA	MIMAT0000230	MI0000713	ACAGUAGUCUGCACAUUGGUU	mmu-miR-199a-3p
500	mmu-miR-200b-3p	5.807	56	mmu-miR-200b-3p	chr4	156055685	156055706	-	miRNA	MIMAT0000233	MI0000243	UAAUACUGCCUGGUAAUGAUG	mmu-miR-200b-3p
501	mmu-miR-203-3p	8.728	424	mmu-miR-203-3p	chr12	112130927	112130948	+	miRNA	MIMAT0000236	MI0000246	GUGAAAUGUUUAGGACCACUA	mmu-miR-203-3p
502	mmu-miR-206-3p	5.858	58	mmu-miR-206-3p	chr1	20679055	20679076	+	miRNA	MIMAT0000239	MI0000249	UGGAAUGUAAGGAAGUGUGUG	mmu-miR-206-3p
503	mmu-miR-143-3p	11.333	2579	mmu-miR-143-3p	chr18	61649199	61649219	-	miRNA	MIMAT0000247	MI0000257	UGAGAUGAAGCACUGUAGCUC	mmu-miR-143-3p
504	mmu-miR-30e-3p	8.798	445	mmu-miR-30e-3p	chr4	120772618	120772639	-	miRNA	MIMAT0000249	MI0000259	CUUUCAGUCGGAUGUUUACAG	mmu-miR-30e-3p
505	mmu-miR-296-3p	1.585	3	mmu-miR-296-3p	chr2	174267058	174267079	-	miRNA	MIMAT0004576	MI0000394	GAGGGUUGGGUGGAGGCUCUC	mmu-miR-296-3p
506	mmu-miR-300-3p	3.170	9	mmu-miR-300-3p	chr12	109724363	109724384	+	miRNA	MIMAT0000378	MI0000400	UAUGCAAGGGCAAGCUCUCUU	mmu-miR-300-3p
507	mmu-miR-301a-3p	2.322	5	mmu-miR-301a-3p	chr11	87113054	87113076	+	miRNA	MIMAT0000379	MI0000401	CAGUGCAAUAGUAUUGUCAAA	mmu-miR-301a-3p
508	mmu-miR-34c-3p	5.044	33	mmu-miR-34c-3p	chr9	51103044	51103065	-	miRNA	MIMAT0004580	MI0000403	AAUCACUAACCACACAGCCAG	mmu-miR-34c-3p
509	mmu-miR-34b-3p	6.000	64	mmu-miR-34b-3p	chr9	51103574	51103595	-	miRNA	MIMAT0004581	MI0000404	AAUCACUAACUCCACUGCCAU	mmu-miR-34b-3p
510	mmu-let-7d-3p	9.276	620	mmu-let-7d-3p	chr13	48536024	48536045	-	miRNA	MIMAT0000384	MI0000405	CUAUACGACCUGCUGCCUUUC	mmu-let-7d-3p
511	mmu-miR-106b-3p	9.433	691	mmu-miR-106b-3p	chr5	138165746	138165767	-	miRNA	MIMAT0004582	MI0000407	CCGCACUGUGGGUACUUGCUG	mmu-miR-106b-3p
512	mmu-miR-130b-3p	2.322	5	mmu-miR-130b-3p	chr16	17124071	17124092	-	miRNA	MIMAT0000387	MI0000408	CAGUGCAAUGAUGAAAGGGCA	mmu-miR-130b-3p
513	mmu-miR-19b-3p	3.807	14	mmu-miR-19b-3p	chr14	115044358	115044380	+	miRNA	MIMAT0000513	MI0000718	UGUGCAAAUCCAUGCAAAACU	mmu-miR-19b-3p
514	mmu-miR-30c-1-3p	3.322	10	mmu-miR-30c-1-3p	chr4	120769546	120769567	-	miRNA	MIMAT0004616	MI0000547	CUGGGAGAGGGUUGUUUACUC	mmu-miR-30c-1-3p
515	mmu-miR-30c-2-3p	6.375	83	mmu-miR-30c-2-3p	chr1	23291754	23291775	+	miRNA	MIMAT0005438	MI0000548	CUGGGAGAAGGCUGUUUACUC	mmu-miR-30c-2-3p
516	mmu-miR-30d-3p	2.585	6	mmu-miR-30d-3p	chr15	68341217	68341238	-	miRNA	MIMAT0017011	MI0000549	CUUUCAGUCAGAUGUUUGCUG	mmu-miR-30d-3p

517	mmu-miR-148a-3p	10.131	1121	mmu-miR-148a-3p	chr6	51269829	51269850	-	miRNA	MIMAT0000516	MI0000550	UCAGUGCACUACAGAACUUUG	mmu-miR-148a-3p
518	mmu-miR-200a-3p	5.700	52	mmu-miR-200a-3p	chr4	156054911	156054932	-	miRNA	MIMAT0000519	MI0000554	UAACACUGUCUGGUAACGAUG	mmu-miR-200a-3p
519	mmu-let-7a-1-3p	4.755	27	mmu-let-7a-1-3p	chr13	48538188	48538209	-	miRNA	MIMAT0004620	MI0000556	CUAUACAAUCUACUGUCUUUC	mmu-let-7a-1-3p
520	mmu-let-7b-3p	6.285	78	mmu-let-7b-3p	chr15	85707379	85707400	+	miRNA	MIMAT0004621	MI0000558	CUAUACAACCUACUGCCUUCC	mmu-let-7b-3p
521	mmu-let-7c-1-3p	3.700	13	mmu-let-7c-1-3p	chr16	77599717	77599738	+	miRNA	MIMAT0004622	MI0000559	CUGUACAACCUUCUAGCUUUC	mmu-let-7c-1-3p
522	mmu-let-7c-2-3p	5.170	36	mmu-let-7c-2-3p	chr15	85706664	85706685	+	miRNA	MIMAT0005439	MI0000560	CUAUACAAUCUACUGUCUUUC	mmu-let-7c-2-3p
523	mmu-let-7e-3p	1.000	2	mmu-let-7e-3p	chr17	17830411	17830432	+	miRNA	MIMAT0017016	MI0000561	CUAUACGGCCUCCUAGCUUUC	mmu-let-7e-3p
524	mmu-let-7f-1-3p	2.585	6	mmu-let-7f-1-3p	chr13	48537833	48537854	-	miRNA	MIMAT0004623	MI0000562	CUAUACAAUCUAUUGCCUUCC	mmu-let-7f-1-3p
525	mmu-let-7f-2-3p	0.000	1	mmu-let-7f-2-3p	chrX	151912403	151912423	+	miRNA	MIMAT0017017	MI0000563	CUAUACAGUCUACUGUCUUUC	mmu-let-7f-2-3p
526	mmu-miR-16-1-3p	1.000	2	mmu-miR-16-1-3p	chr14	61631894	61631915	-	miRNA	MIMAT0004625	MI0000565	CCAGUAUUGACUGUGCUGCUG	mmu-miR-16-1-3p
527	mmu-miR-16-2-3p	1.000	2	mmu-miR-16-2-3p	chr3	69009960	69009981	+	miRNA	MIMAT0017018	MI0000566	ACCAAUAUUAUUGUGCUGCUU	mmu-miR-16-2-3p
528	mmu-miR-18a-3p	4.248	19	mmu-miR-18a-3p	chr14	115043908	115043929	+	miRNA	MIMAT0004626	MI0000567	ACUGCCCUAAGUGCUCCUUCU	mmu-miR-18a-3p
529	mmu-miR-20a-3p	1.585	3	mmu-miR-20a-3p	chr14	115044219	115044240	+	miRNA	MIMAT0004627	MI0000568	ACUGCAUUACGAGCACUUAAA	mmu-miR-20a-3p
530	mmu-miR-22-3p	9.522	735	mmu-miR-22-3p	chr11	75463772	75463793	+	miRNA	MIMAT0000531	MI0000570	AAGCUGCCAGUUGAAGAACUG	mmu-miR-22-3p
531	mmu-miR-23a-3p	7.775	219	mmu-miR-23a-3p	chr8	84208563	84208583	+	miRNA	MIMAT0000532	MI0000571	AUCACAUUGCCAGGGAUUUCC	mmu-miR-23a-3p
532	mmu-miR-26b-3p	1.000	2	mmu-miR-26b-3p	chr1	74394360	74394381	+	miRNA	MIMAT0004630	MI0000575	CCUGUUCUCCAUUACUUGGCU	mmu-miR-26b-3p
533	mmu-miR-29a-3p	10.338	1294	mmu-miR-29a-3p	chr6	31062673	31062694	-	miRNA	MIMAT0000535	MI0000576	UAGCACCAUCUGAAAUCGGUU	mmu-miR-29a-3p
534	mmu-miR-29c-3p	0.000	1	mmu-miR-29c-3p	chr1	195037600	195037621	+	miRNA	MIMAT0000536	MI0000577	UAGCACCAUUUGAAAUCGGUU	mmu-miR-29c-3p
535	mmu-miR-27a-3p	10.102	1099	mmu-miR-27a-3p	chr8	84208727	84208747	+	miRNA	MIMAT0000537	MI0000578	UUCACAGUGGCUAAGUUCCGC	mmu-miR-27a-3p
536	mmu-miR-31-3p	3.322	10	mmu-miR-31-3p	chr4	88910577	88910599	-	miRNA	MIMAT0004634	MI0000579	UGCUAUGCCAACAUAUUGCCA	mmu-miR-31-3p
537	mmu-miR-92a-3p	11.523	2943	mmu-miR-92a-3p	chr14	115044476	115044496	+	miRNA	MIMAT0000539	MI0000719	UAUUGCACUUGUCCCGGCCUG	mmu-miR-92a-3p
538	mmu-miR-129-2-3p	2.807	7	mmu-miR-129-2-3p	chr2	94241376	94241397	-	miRNA	MIMAT0000544	MI0000585	AAGCCCUUACCCCAAAAAGCA	mmu-miR-129-2-3p
539	mmu-miR-98-3p	2.000	4	mmu-miR-98-3p	chrX	151913287	151913308	+	miRNA	MIMAT0017023	MI0000586	CUAUACAACUUACUACUUUCC	mmu-miR-98-3p
540	mmu-miR-103-3p	8.077	270	mmu-miR-103-3p	chr11	35782447	35782469	+	miRNA	MIMAT0000546	MI0000587	AGCAGCAUUGUACAGGGCUAU	mmu-miR-103-3p
541	mmu-miR-322-3p	6.129	70	mmu-miR-322-3p	chrX	53054269	53054289	-	miRNA	MIMAT0000549	MI0000590	AAACAUGAAGCGCUGCAACAC	mmu-miR-322-3p
542	mmu-miR-323-3p	3.000	8	mmu-miR-323-3p	chr12	109712558	109712578	+	miRNA	MIMAT0000551	MI0000592	CACAUUACACGGUCGACCUCU	mmu-miR-323-3p
543	mmu-miR-324-3p	0.000	1	mmu-miR-324-3p	chr11	70012095	70012114	+	miRNA	MIMAT0000556	MI0000595	CCACUGCCCCAGGUGCUGCU	mmu-miR-324-3p
544	mmu-miR-326-3p	1.585	3	mmu-miR-326-3p	chr7	99552328	99552348	+	miRNA	MIMAT0000559	MI0000598	CCUCUGGGCCCUUCCUCCAGU	mmu-miR-326-3p
545	mmu-miR-328-3p	10.237	1207	mmu-miR-328-3p	chr8	105308379	105308400	-	miRNA	MIMAT0000565	MI0000603	CUGGCCCUCUCUGCCCUUCCG	mmu-miR-328-3p

546	mmu-miR-330-3p	5.000	32	mmu-miR-330-3p	chr7	19181525	19181547	+	miRNA	MIMAT0000569	MI0000607	GCAAAGCACAGGGCCUGCAGA	mmu-miR-330-3p
547	mmu-miR-148b-3p	7.426	172	mmu-miR-148b-3p	chr15	103285185	103285206	+	miRNA	MIMAT0000580	MI0000617	UCAGUGCAUCACAGAACUUUG	mmu-miR-148b-3p
548	mmu-miR-339-3p	4.170	18	mmu-miR-339-3p	chr5	139369673	139369695	-	miRNA	MIMAT0004649	MI0000621	UGAGCGCCUCGGCGACAGAGC	mmu-miR-339-3p
549	mmu-miR-340-3p	4.322	20	mmu-miR-340-3p	chr11	50069762	50069783	+	miRNA	MIMAT0000586	MI0000623	UCCGUCUCAGUUACUUUAUAG	mmu-miR-340-3p
550	mmu-miR-342-3p	6.492	90	mmu-miR-342-3p	chr12	108658680	108658702	+	miRNA	MIMAT0000590	MI0000627	UCUCACACAGAAAUCGCACCC	mmu-miR-342-3p
551	mmu-miR-344-3p	4.000	16	mmu-miR-344-3p	chr7	61877782	61877804	-	miRNA	MIMAT0000593	MI0000630	UGAUCUAGCCAAAGCCUGACU	mmu-miR-344-3p
552	mmu-miR-345-3p	7.484	179	mmu-miR-345-3p	chr12	108837027	108837048	+	miRNA	MIMAT0004656	MI0000632	CCUGAACUAGGGGUCUGGAGA	mmu-miR-345-3p
553	mmu-miR-350-3p	0.000	1	mmu-miR-350-3p	chr1	176772342	176772363	-	miRNA	MIMAT0000605	MI0000640	UUCACAAAGCCCAUACACUUU	mmu-miR-350-3p
554	mmu-miR-351-3p	4.858	29	mmu-miR-351-3p	chrX	53053276	53053296	-	miRNA	MIMAT0017042	MI0000643	GGUCAAGAGGCGCCUGGGAAC	mmu-miR-351-3p
555	mmu-miR-135b-3p	0.000	1	mmu-miR-135b-3p	chr1	132198142	132198163	+	miRNA	MIMAT0017044	MI0000646	AUGUAGGGCUAAAAGCCAUGG	mmu-miR-135b-3p
556	mmu-miR-101b-3p	4.000	16	mmu-miR-101b-3p	chr19	29135338	29135356	+	miRNA	MIMAT0000616	MI0000649	GUACAGUACUGUGAUAGCU	mmu-miR-101b-3p
557	mmu-miR-107-3p	6.170	72	mmu-miR-107-3p	chr19	34820700	34820722	-	miRNA	MIMAT0000647	MI0000684	AGCAGCAUUGUACAGGGCUAU	mmu-miR-107-3p
558	mmu-miR-10a-3p	4.392	21	mmu-miR-10a-3p	chr11	96317227	96317248	+	miRNA	MIMAT0004659	MI0000685	CAAAUUCGUAUCUAGGGGAAU	mmu-miR-10a-3p
559	mmu-miR-17-3p	4.524	23	mmu-miR-17-3p	chr14	115043721	115043742	+	miRNA	MIMAT0000650	MI0000687	ACUGCAGUGAGGGCACUUGUA	mmu-miR-17-3p
560	mmu-miR-19a-3p	4.392	21	mmu-miR-19a-3p	chr14	115044048	115044070	+	miRNA	MIMAT0000651	MI0000688	UGUGCAAAUCUAUGCAAAACU	mmu-miR-19a-3p
561	mmu-miR-25-3p	12.032	4189	mmu-miR-25-3p	chr5	138165332	138165353	-	miRNA	MIMAT0000652	MI0000689	CAUUGCACUUGUCUCGGUCUG	mmu-miR-25-3p
562	mmu-miR-28a-3p	6.907	120	mmu-miR-28a-3p	chr16	24827908	24827929	+	miRNA	MIMAT0004661	MI0000690	CACUAGAUUGUGAGCUGCUGG	mmu-miR-28a-3p
563	mmu-miR-32-3p	3.170	9	mmu-miR-32-3p	chr4	56895232	56895252	-	miRNA	MIMAT0017050	MI0000691	CAAUUUAGUGUGUGUGAUAUU	mmu-miR-32-3p
564	mmu-miR-139-3p	0.000	1	mmu-miR-139-3p	chr7	101475418	101475439	+	miRNA	MIMAT0004662	MI0000693	UGGAGACGCGGCCCUGUUGGA	mmu-miR-139-3p
565	mmu-miR-200c-3p	7.140	141	mmu-miR-200c-3p	chr6	124718324	124718346	-	miRNA	MIMAT0000657	MI0000694	UAAUACUGCCGGGUAAUGAUG	mmu-miR-200c-3p
566	mmu-miR-210-3p	2.000	4	mmu-miR-210-3p	chr7	141221407	141221428	-	miRNA	MIMAT0000658	MI0000695	CUGUGCGUGUGACAGCGGCUG	mmu-miR-210-3p
567	mmu-miR-181a-1-3p	3.585	12	mmu-miR-181a-1-3p	chr1	137966508	137966529	+	miRNA	MIMAT0000660	MI0000697	ACCAUCGACCGUUGAUUGUAC	mmu-miR-181a-1-3p
568	mmu-miR-214-3p	0.000	1	mmu-miR-214-3p	chr1	162223438	162223459	+	miRNA	MIMAT0000661	MI0000698	ACAGCAGGCACAGACAGGCAG	mmu-miR-214-3p
569	mmu-miR-216a-3p	4.858	29	mmu-miR-216a-3p	chr11	28757058	28757079	+	miRNA	MIMAT0017054	MI0000699	CACAGUGGUCUCUGGGAUUAU	mmu-miR-216a-3p
570	mmu-miR-218-2-3p	0.000	1	mmu-miR-218-2-3p	chr11	35616882	35616903	+	miRNA	MIMAT0005444	MI0000701	CAUGGUUCUGUCAAGCACCGC	mmu-miR-218-2-3p
571	mmu-miR-219a-1-3p	3.700	13	mmu-miR-219a-1-3p	chr17	34025010	34025031	-	miRNA	MIMAT0017055	MI0000702	AGAGUUGCGUCUGGACGUCCC	mmu-miR-219a-1-3p
572	mmu-miR-223-3p	4.248	19	mmu-miR-223-3p	chrX	96242884	96242905	+	miRNA	MIMAT0000665	MI0000703	UGUCAGUUUGUCAAAUACCCC	mmu-miR-223-3p
573	mmu-miR-320-3p	11.055	2127	mmu-miR-320-3p	chr14	70443557	70443578	+	miRNA	MIMAT0000666	MI0000704	AAAAGCUGGGUUGAGAGGGCG	mmu-miR-320-3p
574	mmu-miR-26a-2-3p	2.000	4	mmu-miR-26a-2-3p	chr10	126995581	126995602	+	miRNA	MIMAT0017058	MI0000706	CCUGUUCUUGAUUACUUGUUU	mmu-miR-26a-2-3p

575	mmu-miR-211-3p	7.989	254	mmu-miR-211-3p	chr7	64205868	64205888	+	miRNA	MIMAT0017059	MI0000708	GCAAGGACAGCAAAGGGGGGC	mmu-miR-211-3p
576	mmu-miR-221-3p	9.994	1020	mmu-miR-221-3p	chrX	19146307	19146329	-	miRNA	MIMAT0000669	MI0000709	AGCUACAUUGUCUGCUGGGUU	mmu-miR-221-3p
577	mmu-miR-222-3p	7.948	247	mmu-miR-222-3p	chrX	19146901	19146923	-	miRNA	MIMAT0000670	MI0000710	AGCUACAUCUGGCUACUGGGU	mmu-miR-222-3p
578	mmu-miR-199b-3p	8.195	293	mmu-miR-199b-3p	chr2	32318524	32318545	+	miRNA	MIMAT0004667	MI0000714	ACAGUAGUCUGCACAUUGGUU	mmu-miR-199b-3p
579	mmu-miR-181b-1-3p	2.000	4	mmu-miR-181b-1-3p	chr1	137966690	137966710	+	miRNA	MIMAT0017067	MI0000723	CUCACUGAACAAUGAAUGCAA	mmu-miR-181b-1-3p
580	mmu-miR-181c-3p	1.000	2	mmu-miR-181c-3p	chr8	84178885	84178906	-	miRNA	MIMAT0017068	MI0000724	ACCAUCGACCGUUGAGUGGAC	mmu-miR-181c-3p
581	mmu-miR-125b-1-3p	2.807	7	mmu-miR-125b-1-3p	chr9	41581980	41582001	+	miRNA	MIMAT0004669	MI0000725	ACGGGUUAGGCUCUUGGGAGC	mmu-miR-125b-1-3p
582	mmu-miR-7a-1-3p	2.322	5	mmu-miR-7a-1-3p	chr13	58392800	58392821	-	miRNA	MIMAT0004670	MI0000728	CAACAAAUCACAGUCUGCCAU	mmu-miR-7a-1-3p
583	mmu-miR-219a-2-3p	3.807	14	mmu-miR-219a-2-3p	chr2	29845645	29845666	-	miRNA	MIMAT0022841	MI0000741	AGAAUUGUGGCUGGACAUCUG	mmu-miR-219a-2-3p
584	mmu-miR-361-3p	7.248	152	mmu-miR-361-3p	chrX	113074827	113074851	-	miRNA	MIMAT0017075	MI0000761	UCCCCCAGGUGUGAUUCUGAU	mmu-miR-361-3p
585	mmu-miR-362-3p	4.459	22	mmu-miR-362-3p	chrX	7241984	7242005	-	miRNA	MIMAT0004684	MI0000763	AACACACCUGUUCAAGGAUUC	mmu-miR-362-3p
586	mmu-miR-363-3p	3.700	13	mmu-miR-363-3p	chrX	52741697	52741718	-	miRNA	MIMAT0000708	MI0000765	AAUUGCACGGUAUCCAUCUGU	mmu-miR-363-3p
587	mmu-miR-365-3p	1.000	2	mmu-miR-365-3p	chr11	79726467	79726488	+	miRNA	MIMAT0000711	MI0001645	UAAUGCCCCUAAAAAUCCUUA	mmu-miR-365-3p
588	mmu-miR-375-3p	3.170	9	mmu-miR-375-3p	chr1	74900661	74900682	-	miRNA	MIMAT0000739	MI0000792	UUUGUUCGUUCGGCUCGCGUG	mmu-miR-375-3p
589	mmu-miR-378a-3p	9.844	919	mmu-miR-378a-3p	chr18	61397838	61397858	-	miRNA	MIMAT0003151	MI0000795	ACUGGACUUGGAGUCAGAAGG	mmu-miR-378a-3p
590	mmu-miR-379-3p	5.129	35	mmu-miR-379-3p	chr12	109709102	109709123	+	miRNA	MIMAT0017080	MI0000796	UAUGUAACAUGGUCCACUAAC	mmu-miR-379-3p
591	mmu-miR-381-3p	4.322	20	mmu-miR-381-3p	chr12	109726870	109726891	+	miRNA	MIMAT0000746	MI0000798	UAUACAAGGGCAAGCUCUCUG	mmu-miR-381-3p
592	mmu-miR-382-3p	2.000	4	mmu-miR-382-3p	chr12	109733819	109733840	+	miRNA	MIMAT0004691	MI0000799	UCAUUCACGGACAACACUUUU	mmu-miR-382-3p
593	mmu-miR-335-3p	6.672	102	mmu-miR-335-3p	chr6	30741354	30741375	+	miRNA	MIMAT0004704	MI0000817	UUUUUCAUUAUUGCUCCUGAC	mmu-miR-335-3p
594	mmu-miR-409-3p	7.257	153	mmu-miR-409-3p	chr12	109743204	109743225	+	miRNA	MIMAT0001090	MI0001160	GAAUGUUGCUCGGUGAACCCC	mmu-miR-409-3p
595	mmu-miR-410-3p	0.000	1	mmu-miR-410-3p	chr12	109743764	109743784	+	miRNA	MIMAT0001091	MI0001161	AAUAUAACACAGAUGGCCUGU	mmu-miR-410-3p
596	mmu-miR-411-3p	5.322	40	mmu-miR-411-3p	chr12	109710225	109710246	+	miRNA	MIMAT0001093	MI0001163	UAUGUAACACGGUCCACUAAC	mmu-miR-411-3p
597	mmu-miR-370-3p	2.585	6	mmu-miR-370-3p	chr12	109618305	109618326	+	miRNA	MIMAT0001095	MI0001165	GCCUGCUGGGGUGGAACCUGG	mmu-miR-370-3p
598	mmu-miR-433-3p	6.209	74	mmu-miR-433-3p	chr12	109591781	109591802	+	miRNA	MIMAT0001420	MI0001525	AUCAUGAUGGGCUCCUCGGUG	mmu-miR-433-3p
599	mmu-miR-448-3p	1.000	2	mmu-miR-448-3p	chrX	147158281	147158302	+	miRNA	MIMAT0001533	MI0001638	UUGCAUAUGUAGGAUGUCCCA	mmu-miR-448-3p
600	mmu-miR-429-3p	0.000	1	mmu-miR-429-3p	chr4	156053916	156053937	-	miRNA	MIMAT0001537	MI0001642	UAAUACUGUCUGGUAAUGCCG	mmu-miR-429-3p
601	mmu-miR-450a-1-3p	1.000	2	mmu-miR-450a-1-3p	chrX	53048171	53048192	-	miRNA	MIMAT0017182	MI0001653	AUUGGGAACAUUUUGCAUAAA	mmu-miR-450a-1-3p
602	mmu-miR-468-3p	1.000	2	mmu-miR-468-3p	chr6	81896602	81896624	-	miRNA	MIMAT0002109	MI0002403	UAUGACUGAUGUGCGUGUGUC	mmu-miR-468-3p
603	mmu-miR-532-3p	3.907	15	mmu-miR-532-3p	chrX	7248419	7248440	-	miRNA	MIMAT0004781	MI0003206	CCUCCCACACCCAAGGCUUGC	mmu-miR-532-3p

604	mmu-miR-483-3p	1.000	2	mmu-miR-483-3p	chr7	142654932	142654952	-	miRNA	MIMAT0003120	MI0003484	UCACUCCUCCCCUCCCGUCUU	mmu-miR-483-3p
605	mmu-miR-485-3p	1.585	3	mmu-miR-485-3p	chr12	109734946	109734967	+	miRNA	MIMAT0003129	MI0003492	AGUCAUACACGGCUCUCCUCU	mmu-miR-485-3p
606	mmu-miR-486a-3p	7.180	145	mmu-miR-486a-3p	chr8	23142629	23142649	+	miRNA	MIMAT0017206	MI0003493	CGGGGCAGCUCAGUACAGGAU	mmu-miR-486a-3p
607	mmu-miR-543-3p	6.322	80	mmu-miR-543-3p	chr12	109717304	109717325	+	miRNA	MIMAT0003168	MI0003519	AAACAUUCGCGGUGCACUUCU	mmu-miR-543-3p
608	mmu-miR-542-3p	4.392	21	mmu-miR-542-3p	chrX	53049415	53049436	-	miRNA	MIMAT0003172	MI0003522	UGUGACAGAUUGAUAACUGAA	mmu-miR-542-3p
609	mmu-miR-494-3p	3.000	8	mmu-miR-494-3p	chr12	109715367	109715388	+	miRNA	MIMAT0003182	MI0003532	UGAAACAUACACGGGAAACCU	mmu-miR-494-3p
610	mmu-miR-487b-3p	4.000	16	mmu-miR-487b-3p	chr12	109727382	109727403	+	miRNA	MIMAT0003184	MI0003534	AAUCGUACAGGGUCAUCCACU	mmu-miR-487b-3p
611	mmu-miR-369-3p	3.170	9	mmu-miR-369-3p	chr12	109743466	109743486	+	miRNA	MIMAT0003186	MI0003535	AAUAAUACAUGGUUGAUCUUU	mmu-miR-369-3p
612	mmu-miR-503-3p	3.170	9	mmu-miR-503-3p	chrX	53053987	53054008	-	miRNA	MIMAT0004790	MI0003538	GAGUAUUGUUUCCACUGCCUG	mmu-miR-503-3p
613	mmu-miR-1224-3p	0.000	1	mmu-miR-1224-3p	chr16	20604516	20604536	+	miRNA	MIMAT0017231	MI0004118	CCCCACCUCUUCUCUCCUCAG	mmu-miR-1224-3p
614	mmu-miR-744-3p	0.000	1	mmu-miR-744-3p	chr11	65734742	65734763	-	miRNA	MIMAT0004820	MI0004124	CUGUUGCCACUAACCUCAACC	mmu-miR-744-3p
615	mmu-miR-1249-3p	6.741	107	mmu-miR-1249-3p	chr15	84951545	84951566	-	miRNA	MIMAT0010560	MI0004132	ACGCCCUUCCCCCCCUUCUUCA	mmu-miR-1249-3p
616	mmu-miR-671-3p	7.119	139	mmu-miR-671-3p	chr5	24592171	24592191	+	miRNA	MIMAT0004821	MI0004133	UCCGGUUCUCAGGGCUCCACC	mmu-miR-671-3p
617	mmu-miR-1843a-3p	4.322	20	mmu-miR-1843a-3p	chr12	80391615	80391636	-	miRNA	MIMAT0014806	MI0004155	UCUGAUCGUUCACCUCCAUACA	mmu-miR-1843a-3p
618	mmu-miR-665-3p	0.000	1	mmu-miR-665-3p	chr12	109586368	109586387	+	miRNA	MIMAT0003733	MI0004171	ACCAGGAGGCUGAGGUCCCU	mmu-miR-665-3p
619	mmu-miR-770-3p	3.807	14	mmu-miR-770-3p	chr12	109563744	109563765	+	miRNA	MIMAT0003891	MI0004203	CGUGGGCCUGACGUGGAGCUG	mmu-miR-770-3p
620	mmu-miR-344d-3p	5.170	36	mmu-miR-344d-3p	chr7	61683127	61683148	-	miRNA	MIMAT0014808	MI0004524	GAUAUAACCACUGCCAGACUGA	mmu-miR-344d-3p
621	mmu-miR-670-3p	1.585	3	mmu-miR-670-3p	chr2	94261316	94261340	-	miRNA	MIMAT0017242	MI0004295	UUUCCUCAUAUCCAUUCAGGA	mmu-miR-670-3p
622	mmu-miR-669a-3p	0.000	1	mmu-miR-669a-3p	chr2	10476908	10476930	+	miRNA	MIMAT0017243	MI0004523	ACAUAACAUACACACACACGUA	mmu-miR-669a-3p
623	mmu-miR-496a-3p	1.000	2	mmu-miR-496a-3p	chr12	109739165	109739186	+	miRNA	MIMAT0003738	MI0004589	UGAGUAUUACAUGGCCAAUCU	mmu-miR-496a-3p
624	mmu-miR-760-3p	4.459	22	mmu-miR-760-3p	chr3	122293615	122293634	-	miRNA	MIMAT0003898	MI0004605	CGGCUCUGGGUCUGUGGGGA	mmu-miR-760-3p
625	mmu-miR-674-3p	9.733	851	mmu-miR-674-3p	chr2	117185186	117185207	+	miRNA	MIMAT0003741	MI0004611	CACAGCUCCCAUCUCAGAACAA	mmu-miR-674-3p
626	mmu-miR-423-3p	12.047	4233	mmu-miR-423-3p	chr11	77078086	77078108	-	miRNA	MIMAT0003454	MI0004637	AGCUCGGUCUGAGGCCCCUCA	mmu-miR-423-3p
627	mmu-miR-495-3p	5.555	47	mmu-miR-495-3p	chr12	109718795	109718816	+	miRNA	MIMAT0003456	MI0004639	AAACAAACAUGGUGCACUUCU	mmu-miR-495-3p
628	mmu-miR-669a-3-3p	0.000	1	mmu-miR-669a-3-3p	chr2	10474496	10474518	+	miRNA	MIMAT0017251	MI0004668	ACAUAACAUACACACACAUGUA	mmu-miR-669a-3-3p
629	mmu-miR-669c-3p	2.585	6	mmu-miR-669c-3p	chr2	10509359	10509380	+	miRNA	MIMAT0017253	MI0004673	UACACACACACACACAAGUAAA	mmu-miR-669c-3p
630	mmu-miR-455-3p	2.322	5	mmu-miR-455-3p	chr4	63256904	63256924	+	miRNA	MIMAT0003742	MI0004679	GCAGUCCACGGGCAUAUACAC	mmu-miR-455-3p
631	mmu-miR-698-3p	0.000	1	mmu-miR-698-3p	chr4	124743852	124743870	+	miRNA	MIMAT0003488	MI0004682	CAUUCUCGUUUCCUUCCCU	mmu-miR-698-3p
632	mmu-miR-700-3p	6.555	94	mmu-miR-700-3p	chr4	135416561	135416581	-	miRNA	MIMAT0003490	MI0004684	CACGCGGGAACCGAGUCCACC	mmu-miR-700-3p

633	mmu-miR-702-3p	0.000	1	mmu-miR-702-3p	chr5	136991520	136991541	+	miRNA	MIMAT0003492	MI0004686	UGCCCACCCUUUACCCCGCUC	mmu-miR-702-3p
634	mmu-miR-708-3p	4.644	25	mmu-miR-708-3p	chr7	96249496	96249517	+	miRNA	MIMAT0003498	MI0004692	CAACUAGACUGUGAGCUUCUA	mmu-miR-708-3p
635	mmu-miR-500-3p	2.585	6	mmu-miR-500-3p	chrX	7237695	7237716	-	miRNA	MIMAT0003507	MI0004702	AAUGCACCUGGGCAAGGUUC	mmu-miR-500-3p
636	mmu-miR-501-3p	8.422	343	mmu-miR-501-3p	chrX	7241270	7241291	-	miRNA	MIMAT0003509	MI0004703	AAUGCACCCGGGCAAGGAUUU	mmu-miR-501-3p
637	mmu-miR-450b-3p	4.322	20	mmu-miR-450b-3p	chrX	53048008	53048029	-	miRNA	MIMAT0003512	MI0004705	AUUGGGAACAUUUUGCAUGCA	mmu-miR-450b-3p
638	mmu-miR-652-3p	3.807	14	mmu-miR-652-3p	chrX	142739060	142739080	+	miRNA	MIMAT0003711	MI0004965	AAUGGCGCCACUAGGGUUGUG	mmu-miR-652-3p
639	mmu-miR-676-3p	1.000	2	mmu-miR-676-3p	chrX	100381151	100381171	+	miRNA	MIMAT0003782	MI0005003	CCGUCCUGAGGUUGUUGAGCU	mmu-miR-676-3p
640	mmu-miR-615-3p	9.119	556	mmu-miR-615-3p	chr15	103014969	103014990	+	miRNA	MIMAT0003783	MI0005004	UCCGAGCCUGGGUCUCCCUCU	mmu-miR-615-3p
641	mmu-miR-871-3p	1.585	3	mmu-miR-871-3p	chrX	66810438	66810460	-	miRNA	MIMAT0017265	MI0005471	UGACUGGCACCAUUCUGGAUA	mmu-miR-871-3p
642	mmu-miR-879-3p	1.000	2	mmu-miR-879-3p	chr5	9375748	9375769	+	miRNA	MIMAT0004843	MI0005472	GCUUAUGGCUUCAAGCUUUCG	mmu-miR-879-3p
643	mmu-miR-881-3p	3.459	11	mmu-miR-881-3p	chrX	66801954	66801975	-	miRNA	MIMAT0004846	MI0005474	AACUGUGUCUUUUCUGAAUAG	mmu-miR-881-3p
644	mmu-miR-883a-3p	1.000	2	mmu-miR-883a-3p	chrX	66780768	66780789	-	miRNA	MIMAT0004849	MI0005476	UAACUGCAACAGCUCUCAGUA	mmu-miR-883a-3p
645	mmu-miR-874-3p	3.322	10	mmu-miR-874-3p	chr13	58023135	58023156	-	miRNA	MIMAT0004853	MI0005479	CUGCCCUGGCCCGAGGGACCG	mmu-miR-874-3p
646	mmu-miR-193b-3p	0.000	1	mmu-miR-193b-3p	chr16	13449570	13449591	+	miRNA	MIMAT0004859	MI0005484	AACUGGCCCACAAAGUCCCGC	mmu-miR-193b-3p
647	mmu-miR-297a-3p	0.000	1	mmu-miR-297a-3p	chr2	10515869	10515890	+	miRNA	MIMAT0004864	MI0005488	UAUACAUACACACAUACCCAU	mmu-miR-297a-3p
648	mmu-miR-421-3p	6.087	68	mmu-miR-421-3p	chrX	103572930	103572952	-	miRNA	MIMAT0004869	MI0005496	AUCAACAGACAUUAAUUGGGC	mmu-miR-421-3p
649	mmu-miR-466f-3p	2.322	5	mmu-miR-466f-3p	chr2	10466998	10467018	+	miRNA	MIMAT0004882	MI0005507	CAUACACACACACAUACACAC	mmu-miR-466f-3p
650	mmu-miR-466h-3p	0.000	1	mmu-miR-466h-3p	chr2	10514940	10514958	+	miRNA	MIMAT0017274	MI0005511	UACGCACGCACACACACAC	mmu-miR-466h-3p
651	mmu-miR-504-3p	0.000	1	mmu-miR-504-3p	chrX	59097669	59097690	-	miRNA	MIMAT0017277	MI0005515	AGGGAGAGCAGGGCAGGGUUU	mmu-miR-504-3p
652	mmu-miR-574-3p	6.392	84	mmu-miR-574-3p	chr5	64970364	64970385	+	miRNA	MIMAT0004894	MI0005518	CACGCUCAUGCACACACCCAC	mmu-miR-574-3p
653	mmu-miR-654-3p	1.000	2	mmu-miR-654-3p	chr12	109723270	109723291	+	miRNA	MIMAT0004898	MI0005520	UAUGUCUGCUGACCAUCACCU	mmu-miR-654-3p
654	mmu-miR-92b-3p	6.340	81	mmu-miR-92b-3p	chr3	89227126	89227147	-	miRNA	MIMAT0004899	MI0005521	UAUUGCACUCGUCCCGGCCUC	mmu-miR-92b-3p
655	mmu-miR-872-3p	0.000	1	mmu-miR-872-3p	chr4	94665206	94665227	+	miRNA	MIMAT0004935	MI0005549	UGAACUAUUGCAGUAGCCUCC	mmu-miR-872-3p
656	mmu-miR-873a-3p	0.000	1	mmu-miR-873a-3p	chr4	36668520	36668540	-	miRNA	MIMAT0017279	MI0005550	GAGACUGACAAGUUCCCGGGA	mmu-miR-873a-3p
657	mmu-miR-208b-3p	0.000	1	mmu-miR-208b-3p	chr14	54975710	54975731	-	miRNA	MIMAT0004939	MI0005552	AUAAGACGAACAAAAGGUUUG	mmu-miR-208b-3p
658	mmu-miR-877-3p	3.807	14	mmu-miR-877-3p	chr17	35960731	35960752	-	miRNA	MIMAT0004862	MI0005553	UGUCCUCUUCUCCCUCCUCCC	mmu-miR-877-3p
659	mmu-miR-653-3p	0.000	1	mmu-miR-653-3p	chr6	3721315	3721335	-	miRNA	MIMAT0017284	MI0005557	UUCACUGGAGUUUGUUUCAGU	mmu-miR-653-3p
660	mmu-miR-582-3p	2.000	4	mmu-miR-582-3p	chr13	109324790	109324810	+	miRNA	MIMAT0005292	MI0006127	UAACCUGUUGAACAACUGAAC	mmu-miR-582-3p
661	mmu-miR-669e-3p	0.000	1	mmu-miR-669e-3p	chr2	10467574	10467594	+	miRNA	MIMAT0017330	MI0006300	UGAAUAUACACACACUUACAC	mmu-miR-669e-3p

662	mmu-miR-1941-3p	2.000	4	mmu-miR-1941-3p	chr15	101369401	101369421	+	miRNA	MIMAT0009406	MI0009930	CAUCUUAGCAGUAUCUCCCAU	mmu-miR-1941-3p
663	mmu-miR-1306-3p	3.170	9	mmu-miR-1306-3p	chr16	18284248	18284268	-	miRNA	MIMAT0009411	MI0009935	ACGUUGGCUCUGGUGGUGAUG	mmu-miR-1306-3p
664	mmu-miR-1964-3p	7.814	225	mmu-miR-1964-3p	chr7	29773344	29773365	+	miRNA	MIMAT0009437	MI0009961	CCGACUUCUGGGCUCCGGCUU	mmu-miR-1964-3p
665	mmu-miR-1968-3p	1.000	2	mmu-miR-1968-3p	chr8	13189035	13189055	-	miRNA	MIMAT0017350	MI0009965	ACCACCUCUGCAGCUGUUAAG	mmu-miR-1968-3p
666	mmu-miR-1839-3p	1.585	3	mmu-miR-1839-3p	chr7	81529958	81529980	+	miRNA	MIMAT0009457	MI0009991	AGACCUACUUAUCUACCAACA	mmu-miR-1839-3p
667	mmu-miR-1981-3p	2.322	5	mmu-miR-1981-3p	chr1	184822409	184822429	-	miRNA	MIMAT0017351	MI0009992	CAUCUAACCCUGGCCUUUGAC	mmu-miR-1981-3p
668	mmu-miR-664-3p	1.000	2	mmu-miR-664-3p	chr1	185243012	185243033	+	miRNA	MIMAT0012774	MI0012531	UAUUCAUUUACUCCCCAGCCU	mmu-miR-664-3p
669	mmu-miR-3057-3p	0.000	1	mmu-miR-3057-3p	chr10	81271654	81271675	+	miRNA	MIMAT0014823	MI0014020	UCCCACAGGCCCAGCUCAUAG	mmu-miR-3057-3p
670	mmu-miR-3060-3p	3.170	9	mmu-miR-3060-3p	chr11	4139413	4139434	+	miRNA	MIMAT0014827	MI0014022	CCAUAGCACAGAAGCACUCCC	mmu-miR-3060-3p
671	mmu-miR-3061-3p	3.322	10	mmu-miR-3061-3p	chr11	52126804	52126825	+	miRNA	MIMAT0014829	MI0014023	CUACCUUUGAUAGUCCACUGC	mmu-miR-3061-3p
672	mmu-miR-3065-3p	1.585	3	mmu-miR-3065-3p	chr11	120014819	120014841	+	miRNA	MIMAT0014837	MI0014027	UCAGCACCAGGAUAUUGUUGG	mmu-miR-3065-3p
673	mmu-miR-3066-3p	2.322	5	mmu-miR-3066-3p	chr12	17355441	17355462	+	miRNA	MIMAT0014839	MI0014028	CACUUAAUAGACCGCAACCUG	mmu-miR-3066-3p
674	mmu-miR-3067-3p	0.000	1	mmu-miR-3067-3p	chr12	81166201	81166223	+	miRNA	MIMAT0014841	MI0014029	CCAAGCGGCUGCCCUGGGAGA	mmu-miR-3067-3p
675	mmu-miR-3068-3p	3.700	13	mmu-miR-3068-3p	chr12	87437685	87437706	-	miRNA	MIMAT0014843	MI0014030	GGUGAAUUGCAGUACUCCAAC	mmu-miR-3068-3p
676	mmu-miR-3071-3p	0.000	1	mmu-miR-3071-3p	chr12	109595329	109595350	-	miRNA	MIMAT0014851	MI0014034	AUCAUCAAAACAAAUGGAGUC	mmu-miR-3071-3p
677	mmu-miR-3074-1-3p	3.459	11	mmu-miR-3074-1-3p	chr13	63301211	63301232	-	miRNA	MIMAT0014857	MI0014037	GAUAUCAGCUCAGUAGGCACC	mmu-miR-3074-1-3p
678	mmu-miR-3081-3p	4.322	20	mmu-miR-3081-3p	chr16	44558058	44558080	-	miRNA	MIMAT0014871	MI0014044	UUGCGCUCCGAUCUCUGAGCU	mmu-miR-3081-3p
679	mmu-miR-3084-3p	2.000	4	mmu-miR-3084-3p	chr19	24942240	24942260	-	miRNA	MIMAT0014877	MI0014047	UUCUGCCAGUCUCCUUCAGAC	mmu-miR-3084-3p
680	mmu-miR-466m-3p	2.322	5	mmu-miR-466m-3p	chr2	10466713	10466734	+	miRNA	MIMAT0014883	MI0014050	UACAUACACACAUACACACGC	mmu-miR-466m-3p
681	mmu-miR-669p-3p	3.170	9	mmu-miR-669p-3p	chr2	10489167	10489190	+	miRNA	MIMAT0014890	MI0014064	CAUAACAUACACACACACACG	mmu-miR-669p-3p
682	mmu-miR-466n-3p	0.000	1	mmu-miR-466n-3p	chr2	10513798	10513819	+	miRNA	MIMAT0014894	MI0014079	UAUACAUGAGAGCAUACAUAG	mmu-miR-466n-3p
683	mmu-miR-3095-3p	2.585	6	mmu-miR-3095-3p	chr4	58441023	58441045	-	miRNA	MIMAT0014912	MI0014088	UGGACACUGGAGAGAGAGCUU	mmu-miR-3095-3p
684	mmu-miR-3101-3p	0.000	1	mmu-miR-3101-3p	chr7	27176020	27176040	-	miRNA	MIMAT0014922	MI0014093	UAGCUUUGGUGGAUGGUCUUU	mmu-miR-3101-3p
685	mmu-miR-344b-3p	0.000	1	mmu-miR-344b-3p	chr7	61790522	61790543	-	miRNA	MIMAT0014926	MI0014095	CAUUUAGCCAAAGCCUGACUG	mmu-miR-344b-3p
686	mmu-miR-486b-3p	6.919	121	mmu-miR-486b-3p	chr8	23142587	23142606	-	miRNA	MIMAT0014944	MI0014103	CGGGGCAGCUCAGUACAGGA	mmu-miR-486b-3p
687	mmu-miR-3074-2-3p	7.889	237	mmu-miR-3074-2-3p	chr8	84208839	84208858	-	miRNA	MIMAT0014946	MI0014104	UGUUUCAGCUCAGUAGGCAC	mmu-miR-3074-2-3p
688	mmu-miR-1912-3p	0.000	1	mmu-miR-1912-3p	chrX	147009493	147009514	+	miRNA	MIMAT0014958	MI0014110	CACAGAACAUGCAGUGAGAAC	mmu-miR-1912-3p
689	mmu-miR-3572-3p	0.000	1	mmu-miR-3572-3p	chr7	3656010	3656032	+	miRNA	MIMAT0020636	MI0018037	UACACUUGUCCUUCUUUCCCC	mmu-miR-3572-3p
690	mmu-miR-5129-3p	3.907	15	mmu-miR-5129-3p	chr2	45023110	45023131	-	miRNA	MIMAT0022987	MI0018041	AAUGUGCCUGUGCAUCUCUUC	mmu-miR-5129-3p

691	mmu-miR-5134-3p	3.459	11	mmu-miR-5134-3p	chr17	24234535	24234556	-	miRNA	MIMAT0022989	MI0018046	ACGGGUGGCCCUCUUUCUGCA	mmu-miR-5134-3p
692	mmu-miR-1231-3p	3.170	9	mmu-miR-1231-3p	chr1	135454603	135454625	-	miRNA	MIMAT0022358	MI0019183	UGCCCUGUCUGUUCUGCCCAC	mmu-miR-1231-3p
693	mmu-miR-5620-3p	0.000	1	mmu-miR-5620-3p	chr7	7298926	7298946	+	miRNA	MIMAT0022368	MI0019188	ACAGUCAUCCCCCUGCCUCAC	mmu-miR-5620-3p
694	mmu-miR-5624-3p	4.000	16	mmu-miR-5624-3p	chr13	93790816	93790837	+	miRNA	MIMAT0022378	MI0019193	UUAAGGCAGAGUUUACAAUAG	mmu-miR-5624-3p
695	mmu-miR-5626-3p	0.000	1	mmu-miR-5626-3p	chr9	70405694	70405715	-	miRNA	MIMAT0022382	MI0019195	CAGCAGUUGAGUGAUGUGACA	mmu-miR-5626-3p
696	mmu-miR-6899-3p	4.392	21	mmu-miR-6899-3p	chr1	64042438	64042460	-	miRNA	MIMAT0027699	MI0022746	UUGUCCUUCUGUGUCUUCUGC	mmu-miR-6899-3p
697	mmu-miR-6906-3p	0.000	1	mmu-miR-6906-3p	chr10	60126813	60126833	+	miRNA	MIMAT0027713	MI0022753	GAAUUCCGGUCUCCUCUCCAG	mmu-miR-6906-3p
698	mmu-miR-6913-3p	1.000	2	mmu-miR-6913-3p	chr10	81386390	81386412	+	miRNA	MIMAT0027727	MI0022760	UCUCUACUGAUUUGUCUCCUC	mmu-miR-6913-3p
699	mmu-miR-6921-3p	0.000	1	mmu-miR-6921-3p	chr11	60200538	60200558	-	miRNA	MIMAT0027743	MI0022768	UGACUACUCCUUGCCUCUCAG	mmu-miR-6921-3p
700	mmu-miR-6923-3p	0.000	1	mmu-miR-6923-3p	chr11	67099855	67099875	+	miRNA	MIMAT0027747	MI0022770	ACACUCCCUCCUCCUCCCCAG	mmu-miR-6923-3p
701	mmu-miR-6929-3p	4.170	18	mmu-miR-6929-3p	chr11	101419187	101419209	-	miRNA	MIMAT0027759	MI0022776	CAGGUGCUGUCUUCUUCUUCC	mmu-miR-6929-3p
702	mmu-miR-6939-3p	0.000	1	mmu-miR-6939-3p	chr12	112659277	112659296	-	miRNA	MIMAT0027779	MI0022786	CCUCACUUGACCCGCUGCAG	mmu-miR-6939-3p
703	mmu-miR-6941-3p	0.000	1	mmu-miR-6941-3p	chr12	112920483	112920503	-	miRNA	MIMAT0027783	MI0022788	CUAACUCGGCUGCUCCCAAAG	mmu-miR-6941-3p
704	mmu-miR-6944-3p	1.585	3	mmu-miR-6944-3p	chr13	55477681	55477703	-	miRNA	MIMAT0027789	MI0022791	UAACUCUUCCCUUGUGCCCUC	mmu-miR-6944-3p
705	mmu-miR-6945-3p	1.000	2	mmu-miR-6945-3p	chr13	55507627	55507647	-	miRNA	MIMAT0027791	MI0022792	UCUGAGCUCUGCCCUUCCCAU	mmu-miR-6945-3p
706	mmu-miR-6946-3p	2.000	4	mmu-miR-6946-3p	chr14	20690684	20690703	+	miRNA	MIMAT0027793	MI0022793	UUUCUUCUCUUCCCUUUCAG	mmu-miR-6946-3p
707	mmu-miR-6949-3p	2.000	4	mmu-miR-6949-3p	chr14	56082401	56082421	-	miRNA	MIMAT0027799	MI0022796	UUCCCCUUUUUCUAUCCACAG	mmu-miR-6949-3p
708	mmu-miR-6952-3p	1.000	2	mmu-miR-6952-3p	chr15	76064828	76064849	-	miRNA	MIMAT0027805	MI0022799	UCUCUGACUCUGCCUCCCACA	mmu-miR-6952-3p
709	mmu-miR-6954-3p	0.000	1	mmu-miR-6954-3p	chr15	76433253	76433273	+	miRNA	MIMAT0027809	MI0022801	UGCAGCCAGCUCUUCCCCUAG	mmu-miR-6954-3p
710	mmu-miR-6955-3p	0.000	1	mmu-miR-6955-3p	chr15	78891891	78891912	+	miRNA	MIMAT0027811	MI0022802	ACACCUGUCUCCUUUGCCCAC	mmu-miR-6955-3p
711	mmu-miR-6959-3p	1.585	3	mmu-miR-6959-3p	chr15	89305658	89305679	-	miRNA	MIMAT0027819	MI0022806	CUGCACCGACCUGCUCUCCCA	mmu-miR-6959-3p
712	mmu-miR-6962-3p	0.000	1	mmu-miR-6962-3p	chr15	101193908	101193929	+	miRNA	MIMAT0027825	MI0022809	UAUCUGCCCUUCCCUGUCCUA	mmu-miR-6962-3p
713	mmu-miR-6968-3p	1.585	3	mmu-miR-6968-3p	chr17	26935518	26935539	+	miRNA	MIMAT0027839	MI0022816	ACAAGCGCUGUCUCCCCUCCA	mmu-miR-6968-3p
714	mmu-miR-6975-3p	1.585	3	mmu-miR-6975-3p	chr17	35244116	35244135	+	miRNA	MIMAT0027853	MI0022823	UCUCUCCUUUCUCCUCCUAG	mmu-miR-6975-3p
715	mmu-miR-6977-3p	2.807	7	mmu-miR-6977-3p	chr17	56418850	56418871	-	miRNA	MIMAT0027857	MI0022825	AAGGCGUUGCCUGACCCUGAC	mmu-miR-6977-3p
716	mmu-miR-6979-3p	2.585	6	mmu-miR-6979-3p	chr18	37854605	37854625	-	miRNA	MIMAT0027861	MI0022827	UUGUGUCUGUCUGGCUCCCAG	mmu-miR-6979-3p
717	mmu-miR-6983-3p	0.000	1	mmu-miR-6983-3p	chr18	61117509	61117531	+	miRNA	MIMAT0027869	MI0022831	UGAAUCAAAGUCUGUUGUUCC	mmu-miR-6983-3p
718	mmu-miR-6986-3p	2.000	4	mmu-miR-6986-3p	chr19	4623898	4623916	-	miRNA	MIMAT0027875	MI0022834	GUUUUCACCUUCCUCCCAG	mmu-miR-6986-3p
719	mmu-miR-6988-3p	4.858	29	mmu-miR-6988-3p	chr19	6051371	6051392	+	miRNA	MIMAT0027879	MI0022836	UGACCUCUGUAUCUCCUGCCA	mmu-miR-6988-3p

720	mmu-miR-6994-3p	1.000	2	mmu-miR-6994-3p	chr19	11923778	11923798	+	miRNA	MIMAT0027891	MI0022842	AACGAUCUUCUCCGUCUUUGC	mmu-miR-6994-3p
721	mmu-miR-6996-3p	3.807	14	mmu-miR-6996-3p	chr2	26470059	26470081	-	miRNA	MIMAT0027895	MI0022844	CGGUGUCUCUGGUCACUCUGC	mmu-miR-6996-3p
722	mmu-miR-7001-3p	0.000	1	mmu-miR-7001-3p	chr2	93421929	93421948	-	miRNA	MIMAT0027905	MI0022849	CGCUCACACUCCCUCUGCAG	mmu-miR-7001-3p
723	mmu-miR-6973b-3p	0.000	1	mmu-miR-6973b-3p	chr2	131040326	131040345	-	miRNA	MIMAT0027909	MI0022851	UGCUCUCUUACCCUUCCUAG	mmu-miR-6973b-3p
724	mmu-miR-7012-3p	1.000	2	mmu-miR-7012-3p	chr3	90270191	90270212	+	miRNA	MIMAT0027929	MI0022861	UGACCUGUGGCUCCUCUCCCA	mmu-miR-7012-3p
725	mmu-miR-7019-3p	0.000	1	mmu-miR-7019-3p	chr4	138316132	138316154	-	miRNA	MIMAT0027943	MI0022868	UCACCUUGGCCGCCUCUCUGC	mmu-miR-7019-3p
726	mmu-miR-7020-3p	0.000	1	mmu-miR-7020-3p	chr4	139644091	139644111	+	miRNA	MIMAT0027945	MI0022869	AACCCCUCUCUUCUCUCCCAG	mmu-miR-7020-3p
727	mmu-miR-7022-3p	1.000	2	mmu-miR-7022-3p	chr4	148146937	148146958	-	miRNA	MIMAT0027949	MI0022871	ACAAGCCUGACCUCUGCCCCA	mmu-miR-7022-3p
728	mmu-miR-7028-3p	0.000	1	mmu-miR-7028-3p	chr5	114719557	114719578	+	miRNA	MIMAT0027961	MI0022877	CCUUCUCUUCCCCCUCGGCCA	mmu-miR-7028-3p
729	mmu-miR-7036a-3p	0.000	1	mmu-miR-7036a-3p	chr5	137296624	137296646	-	miRNA	MIMAT0027977	MI0022885	CCGUCCUCAUCCGCUCCUCCCA	mmu-miR-7036a-3p
730	mmu-miR-7041-3p	1.000	2	mmu-miR-7041-3p	chr6	94606364	94606383	-	miRNA	MIMAT0027987	MI0022890	UGGUUCUCUUCUUCCCUCAG	mmu-miR-7041-3p
731	mmu-miR-7044-3p	0.000	1	mmu-miR-7044-3p	chr6	118085243	118085263	+	miRNA	MIMAT0027993	MI0022893	AUGCAGCCCCGACCCUCACAG	mmu-miR-7044-3p
732	mmu-miR-7046-3p	1.585	3	mmu-miR-7046-3p	chr7	24970010	24970031	-	miRNA	MIMAT0027997	MI0022895	UGAACCACCCAUCCCCCUACA	mmu-miR-7046-3p
733	mmu-miR-7050-3p	0.000	1	mmu-miR-7050-3p	chr7	31040258	31040278	-	miRNA	MIMAT0028005	MI0022899	UCUCAGCCUUUAUCUCCCCAG	mmu-miR-7050-3p
734	mmu-miR-7058-3p	3.170	9	mmu-miR-7058-3p	chr7	126367971	126367992	-	miRNA	MIMAT0028021	MI0022907	CUCGUUCCUUCCUUUCUUCCA	mmu-miR-7058-3p
735	mmu-miR-7062-3p	1.000	2	mmu-miR-7062-3p	chr7	139986809	139986831	-	miRNA	MIMAT0028029	MI0022911	ACUAACUUCUCCUGGCCCCAC	mmu-miR-7062-3p
736	mmu-miR-7063-3p	2.585	6	mmu-miR-7063-3p	chr7	141620772	141620791	+	miRNA	MIMAT0028031	MI0022912	UGCUCUCUGCCCCUCUUAAG	mmu-miR-7063-3p
737	mmu-miR-6769b-3p	0.000	1	mmu-miR-6769b-3p	chr8	71631047	71631067	-	miRNA	MIMAT0028041	MI0022917	CAUCUUCCCUGUCCCACCCAG	mmu-miR-6769b-3p
738	mmu-miR-7068-3p	0.000	1	mmu-miR-7068-3p	chr8	72470016	72470036	-	miRNA	MIMAT0028043	MI0022918	UCACCCUGGACUGACUCUCAG	mmu-miR-7068-3p
739	mmu-miR-7073-3p	0.000	1	mmu-miR-7073-3p	chr8	95753151	95753172	-	miRNA	MIMAT0028053	MI0022923	AUAGCCUUUUCCUCCCCAACA	mmu-miR-7073-3p
740	mmu-miR-7074-3p	0.000	1	mmu-miR-7074-3p	chr8	105951637	105951659	-	miRNA	MIMAT0028055	MI0022924	UAGGCCUUCUCCUCUCCCUGC	mmu-miR-7074-3p
741	mmu-miR-7079-3p	0.000	1	mmu-miR-7079-3p	chr8	123105023	123105043	+	miRNA	MIMAT0028065	MI0022929	UGACUUCUGUUCUCUUUCCAG	mmu-miR-7079-3p
742	mmu-miR-7083-3p	1.585	3	mmu-miR-7083-3p	chr9	21809941	21809960	-	miRNA	MIMAT0028073	MI0022933	UCUGCUCUCCUGGACCCCAG	mmu-miR-7083-3p
743	mmu-miR-7084-3p	0.000	1	mmu-miR-7084-3p	chr9	22113951	22113972	-	miRNA	MIMAT0028075	MI0022934	GCCUCUGACCCCUGUCCUCUG	mmu-miR-7084-3p
744	mmu-miR-7086-3p	0.000	1	mmu-miR-7086-3p	chr9	45266643	45266663	-	miRNA	MIMAT0028079	MI0022936	UCCGUUCUGAUAUCUCCUCAG	mmu-miR-7086-3p
745	mmu-miR-7087-3p	7.219	149	mmu-miR-7087-3p	chr9	45939521	45939542	-	miRNA	MIMAT0028081	MI0022937	UGCUGGCUCUCUCCCCUGCCA	mmu-miR-7087-3p
746	mmu-miR-7093-3p	2.807	7	mmu-miR-7093-3p	chrX	134757711	134757731	+	miRNA	MIMAT0028093	MI0022943	UUUCCAUCUGUCAUCCUGCAG	mmu-miR-7093-3p
747	mmu-miR-7212-3p	0.000	1	mmu-miR-7212-3p	chr15	25948267	25948287	+	miRNA	MIMAT0028393	MI0023707	UAACACACACGUCUCCAGGUC	mmu-miR-7212-3p
748	mmu-miR-7214-3p	0.000	1	mmu-miR-7214-3p	chr17	27317035	27317058	-	miRNA	MIMAT0028397	MI0023709	CUCAGUCCUGACCCCUUGAGC	mmu-miR-7214-3p

749	mmu-miR-7221-3p	1.000	2	mmu-miR-7221-3p	chr2	92592257	92592277	+	miRNA	MIMAT0028411	MI0023716	UGACUGUGGGCUGGGGACUGG	mmu-miR-7221-3p
750	mmu-miR-7222-3p	0.000	1	mmu-miR-7222-3p	chr2	92594654	92594676	+	miRNA	MIMAT0028413	MI0023717	UCCAGGACAGUGGGCAGGAGC	mmu-miR-7222-3p
751	mmu-miR-7224-3p	5.585	48	mmu-miR-7224-3p	chr2	67675496	67675516	+	miRNA	MIMAT0028417	MI0023719	UCCACUGAGAGGACCACCCAC	mmu-miR-7224-3p
752	mmu-miR-7226-3p	0.000	1	mmu-miR-7226-3p	chr4	118210505	118210527	-	miRNA	MIMAT0028421	MI0023721	UGACACAGCCAUUCUCUGAGC	mmu-miR-7226-3p
753	mmu-miR-7230-3p	1.585	3	mmu-miR-7230-3p	chr5	113337590	113337612	-	miRNA	MIMAT0028429	MI0023725	UUGAGUUGAGACUGUCAGUAG	mmu-miR-7230-3p
754	mmu-miR-7234-3p	0.000	1	mmu-miR-7234-3p	chr7	73819619	73819641	+	miRNA	MIMAT0028437	MI0023729	AAACGUCUUUCUAGGGUAGAA	mmu-miR-7234-3p
755	mmu-miR-7235-3p	1.585	3	mmu-miR-7235-3p	chr11	97146364	97146386	-	miRNA	MIMAT0028439	MI0023730	UCUGACUUCUUGCUUCUCUCC	mmu-miR-7235-3p
756	mmu-miR-7237-3p	3.000	8	mmu-miR-7237-3p	chr8	121978065	121978086	+	miRNA	MIMAT0028443	MI0023732	CAUCCUGUUGAGCUUACCGAG	mmu-miR-7237-3p
757	mmu-miR-7648-3p	1.000	2	mmu-miR-7648-3p	chr15	90224392	90224412	+	miRNA	MIMAT0029799	MI0024986	AGGGCUGGGCCCGGGACGCGG	mmu-miR-7648-3p
758	mmu-miR-7653-3p	5.170	36	mmu-miR-7653-3p	chr11	78178866	78178887	+	miRNA	MIMAT0029813	MI0024993	CCCCUACUGUUCCACCCCGCC	mmu-miR-7653-3p
759	mmu-miR-7655-3p	0.000	1	mmu-miR-7655-3p	chr2	18057882	18057903	+	miRNA	MIMAT0029817	MI0024995	UUUAUGACGCUCCGUGGGCCU	mmu-miR-7655-3p
760	mmu-miR-7664-3p	2.807	7	mmu-miR-7664-3p	chr2	25059851	25059873	+	miRNA	MIMAT0029835	MI0025004	UCUCUUAGGCUGGGUUAACUA	mmu-miR-7664-3p
761	mmu-miR-7666-3p	1.585	3	mmu-miR-7666-3p	chr8	40307875	40307893	-	miRNA	MIMAT0029839	MI0025006	GAUGCAGCGCACGGGCGAG	mmu-miR-7666-3p
762	mmu-miR-7667-3p	2.322	5	mmu-miR-7667-3p	chr17	29595579	29595600	+	miRNA	MIMAT0029841	MI0025007	AGGGACUGCAGAGAUGGCACU	mmu-miR-7667-3p
763	mmu-miR-7669-3p	3.322	10	mmu-miR-7669-3p	chr3	90011153	90011175	-	miRNA	MIMAT0029845	MI0025009	GAGCUGGGUGUGGUGGCACAU	mmu-miR-7669-3p
764	mmu-miR-7671-3p	0.000	1	mmu-miR-7671-3p	chr11	53763597	53763618	-	miRNA	MIMAT0029849	MI0025011	AAGCGGGCAGGCGGAGAACCA	mmu-miR-7671-3p
765	mmu-miR-7673-3p	1.585	3	mmu-miR-7673-3p	chrX	94274615	94274637	+	miRNA	MIMAT0029853	MI0025013	UUCCUUACCUCACCGUCAAAG	mmu-miR-7673-3p
766	mmu-miR-129b-3p	6.585	96	mmu-miR-129b-3p	chr2	94241420	94241442	+	miRNA	MIMAT0029863	MI0025019	CAAGCCCAGACCGCAAAAAGA	mmu-miR-129b-3p
767	mmu-miR-292b-3p	0.000	1	mmu-miR-292b-3p	chr7	3219198	3219218	-	miRNA	MIMAT0029865	MI0025020	AAGAGCCCCCAGUUUGAGUAU	mmu-miR-292b-3p
768	mmu-miR-7677-3p	0.000	1	mmu-miR-7677-3p	chr17	27091289	27091311	+	miRNA	MIMAT0029869	MI0025022	CCGGCUGUGUCCUCUCACCUA	mmu-miR-7677-3p
769	mmu-miR-7681-3p	0.000	1	mmu-miR-7681-3p	chr1	53849474	53849495	+	miRNA	MIMAT0029883	MI0025029	AGAAAGGGCACUGACAGGAUA	mmu-miR-7681-3p
770	mmu-miR-7682-3p	2.585	6	mmu-miR-7682-3p	chr1	151442430	151442450	+	miRNA	MIMAT0029885	MI0025030	CCUGUGGGUUGGGUUGGCUUU	mmu-miR-7682-3p
771	mmu-miR-126b-3p	3.459	11	mmu-miR-126b-3p	chr2	26591363	26591385	-	miRNA	MIMAT0029895	MI0025035	CGCGUACCAAAAGUAAUAAUG	mmu-miR-126b-3p
772	mmu-miR-1258-3p	3.700	13	mmu-miR-1258-3p	chr18	56538177	56538198	+	miRNA	MIMAT0029905	MI0025040	UUAGGGAAUUAGCUCAGCAGU	mmu-miR-1258-3p
773	mmu-miR-203b-3p	0.000	1	mmu-miR-203b-3p	chr12	112130887	112130908	-	miRNA	MIMAT0049832	MI0040622	UUGAACUGUCAAGAACCACUG	mmu-miR-203b-3p
774	mmu-miR-9b-3p	7.443	174	mmu-miR-9b-3p	chr13	83738821	83738841	-	miRNA	MIMAT0049836	MI0040624	AUACAGCUAGAUAACCAAAGA	mmu-miR-9b-3p
775	mmu-miR-12188-3p	0.000	1	mmu-miR-12188-3p	chr17	25160550	25160576	+	miRNA	MIMAT0049845	MI0040629	UGGACAGCAGUACCCCAGCCC	mmu-miR-12188-3p
776	mmu-miR-122b-3p	10.029	1045	mmu-miR-122b-3p	chr18	65248865	65248886	-	miRNA	MIMAT0049853	MI0040634	AAACACCAUUGUCACACUCCA	mmu-miR-122b-3p
777	mmu-miR-12202-3p	0.000	1	mmu-miR-12202-3p	chr7	4918009	4918030	+	miRNA	MIMAT0049872	MI0040648	UCUUCUCUUUCCAGUCAUCAG	mmu-miR-12202-3p
